# Are children in female-headed households at a disadvantage? An analysis of immunization coverage and stunting prevalence: in 95 low- and middle-income countries

**DOI:** 10.1016/j.ssmph.2021.100888

**Published:** 2021-08-05

**Authors:** Andrea Wendt, Franciele Hellwig, Ghada E. Saad, Cheikh Faye, Zitha Mokomane, Ties Boerma, Aluisio J.D. Barros, Cesar Victora

**Affiliations:** a International Center for Equity in Health, Postgraduate Program of Epidemiology, Federal University of Pelotas, Pelotas, Brazil; b Faculty of Health Sciences, Department of Epidemiology and Population Health, American University of Beirut, Beirut, Lebanon; c African Population and Health Research Center, Nairobi, Kenya; d University of Pretoria, Pretoria, South Africa; e University of Manitoba, Winnipeg, Canada; f International Center for Equity in Health, Federal University of Pelotas, Pelotas, Brazil

**Keywords:** Immunization, Stunting, Female-headed households, Woman-headed households

## Abstract

Studies of inequalities in child health have given limited attention to household structure and headship. The few existing reports on child outcomes in male and female-headed households have produced inconsistent results. The aim of our analyses was to provide a global view of the influence of sex of the household head on child health in cross-sectional surveys from up to 95 LMICs. Studied outcomes were full immunization coverage in children aged 12–23 months and stunting prevalence in under-five children. We analyzed the most recent nationally-representative surveys for each country (since 2010) with available data. After initial exploratory analyses, we focused on three types of households: a) male-headed household (MHH) comprised 73.1% of all households in the pooled analyses; b) female Headed Household (FHH) with at least one adult male represented 9.8% of households; and c) FHH without an adult male accounted for 15.0% of households. Our analyses also included the following covariates: wealth index, education of the child’s mother and urban/rural residence. Meta-analytic approaches were used to calculate pooled effects across the countries with MHH as the reference category. Regarding full immunization, the pooled prevalence ratio for FHH (any male) was 0.99 (0.97; 1.01) and that for FHH (no male) was 0.99 (0.97; 1.02). For stunting prevalence, the pooled prevalence ratio for FHH (any male) was 1.00 (0.98; 1.02) and for FHH (no male) was 1.00 (0.98; 1.02). Adjustment for covariates did not lead to any noteworthy change in the results. No particular patterns were found among different world regions. A few countries presented significant inequalities with different directions of association, indicating the diversity of FHH and how complex the meaning and measurement of household headship may be. Further research is warranted to understand context, examine mediating factors, and exploring alternative definitions of household headship in countries with some association.

## Highlights

• Globally, living in a female-headed household showed no effect on child outcomes.
• A small group of countries presented inequalities in stunting and immunization.
• The direction of female headed household effect on child outcomes varied widely.

## 1 Introduction

Child health is strongly influenced by social determinants (such as gender, ethnicity, and their family’s socioeconomic conditions) (Pearce et al., 2019) and within-country socioeconomic inequalities affect the coverage of health interventions, as well as the health and nutrition of young children (Li et al., 2017; Oberg et al., 2016; Victora et al., 2019). While there has been some progress towards reduction of inequalities in child health within-countries, the process remains slow (Countdown to 2030 Collaboration, 2018) and requires further action against the causes of inequality. The Sustainable Development Goals (SDGs)(UN General Assembly, 2015) include explicit objectives focusing on reduction of socioeconomic disparities in general, as well as highlighting the relevance of gender inequality.

A specific issue that has received limited attention in studies of inequalities in child health is household structure and headship, which may act as determinants of health by influencing socioeconomic position, relationships among family members and stability of families (Conger et al., 2010; Fomby & Cherlin, 2007). The early literature on female-headed households (FHH) mostly described such households as vulnerable and in disadvantage because they often present lower incomes and limited access to essential services. The early studies often explored the concept of feminization of poverty, as women comprise an increasing proportion of people in poverty (Buvinić & Gupta, 1997; Buvinj, 1997). Over the years, however, studies have shown that this is not always true (World Bank, 2018; Chant, 2004; Milazzo & van de Walle, 2017), highlighting the need to understand how households headed by females may differ from those headed by males. The reasons for a household to be headed by a female are multiple – including divorce, widowhood, labor migration of husbands, polygyny, matriarchal social structures and nonmarital childbearing – which possibly have varying impacts on household dynamics and resources (Chant, 2004; Milazzo & van de Walle, 2017). Such variability within female-headed households (FHH) may lead to different child health and nutrition outcomes, and to varying coverage with child health interventions.

A review study focusing on the role of women’s empowerment on child health shows how attributes other than family income may contribute to differences in prevalence of child health outcomes (Richards et al., 2013). The review showed that decision making and role of children’s mother or grandmother in household may be crucial in health outcomes (Richards et al., 2013). For example, regardless of family income, an empowered woman who is the head of the household may optimize the allocation of resources for the children (Miranda, 2006; Oraro et al., 2018; Richards et al., 2013). Thus, the relationship between female headship and child health may result in two opposite perspectives. First, a position of women’s empowerment leading to advantages for the health of children in the household. On the other hand, a disadvantaged position, when the woman involuntarily becomes the household head – for example due to divorce or widowhood - resulting in poverty and discrimination that may lead to negative impacts on child health. Therefore, one may either assume that the effect of FHH on child health may be negative due to impoverishment, or else assume that even in spite of fewer economic resources being available, a child growing up in a FHH may achieve equal or even better health conditions compared to MHH because of prioritization of resource allocation and management towards their wellbeing (Richards et al., 2013).

The variability among FHHs in terms of structure, earnings and life trajectories raises the necessity of assessing how child health indicators perform in FHHs compared to male-headed households (MHH) within different contexts, by studying a large number of countries. Because the studies on this topic are usually limited to one or a few countries, and fail to explore the nuances of FHH groups, multi-country analyses that adopt a detailed typology for FHH are much needed to understand the global situation. Data on immunization coverage and stunting prevalence are available in many national surveys that also collect data on household composition. These two indicators reflect dimensions of child health and nutrition where the mother’s role and position within the household may play an important role (Miranda, 2006; Oraro et al., 2018; Richards et al., 2013). Stunting is the most common form of child undernutrition for which socioeconomic inequalities are very marked (de Onis & Branca, 2016), whereas such inequalities are also present for immunization coverage, essential condition to the health and well-being of children (WHO, 2016). In addition, the two outcomes under study are key SDG indicators (goals 2 and 3) (UN General Assembly, 2015).

So far, the limited literature on how household headship may influence immunization coverage or stunting has been based mainly on single country studies. Two studies, one from Punjab (Pakistan) and a national study from Nepal found that children in FHH presented a prevalence of stunting about 25% lower than children in MHH, even after adjusting for economic characteristics (Dorsey et al., 2018; Khalid & Martin, 2017). Regarding vaccination, a national study in Ethiopia found an adjusted prevalence of full immunization 49% lower in FHH, compared to MHH (Geweniger & Abbas, 2020) while a national study from India found the opposite, with children living in a FHH presenting 24% higher coverage of full immunization in comparison to MHH (Basant Kumar Panda & Mishra, 2020). These results exemplify the diversity of associations in different contexts of FHH, and in terms of different health and nutrition outcomes. It should be noted that all of these studies relied upon a simple typology comparing MHH versus FHH.

In order to provide a comprehensive, global view of the potential role of household headship on child health and nutrition, we compared full immunization coverage and stunting prevalence according to sex of head of household in up to 95 low- and middle-income countries (LMICs). Based upon the existing literature, we addressed three hypotheses: a) that FHH would be poorer than MHH; b) that differences in child health and nutrition in the crude analyses would not be marked or systematic; and c) that due to FHH being poorer than MHH, the adjusted analyses would show an advantage for children living in FHH.

## 2 Methods

The International Center for Equity in Health database (www.equidade.org/surveys) includes over 400 surveys carried out in 120 countries since the 1990s, mostly Demographic and Health Survey (DHS) and Multiple Indicator Cluster Survey (MICS). The DHS and MICS are representative national cross-sectional household surveys for monitoring health and nutrition indicators for children and women of reproductive age. DHS and MICS are highly comparable in terms of their multistage sampling procedures, questionnaires, anthropometric protocols, and indicators (Hancioglu & Arnold, 2013). Although MICS includes interviews with the child’s caretaker and DHS with the biological mother, these are very often the same person, and this difference seems not to affect the child indicators (Hancioglu & Arnold, 2013). DHS surveys include in their sample household members who may not be usual residents – that is, visitors who slept in the house in the preceding night – whereas MICS only includes usual residents. To increase the comparability between types of surveys, we excluded visiting children from the DHS sample.

More information about the surveys is available elsewhere (Corsi et al., 2012; Murray & Newby, 2012). We included in these analyses the most recent survey for each country (since 2010) with available data on child immunization and nutritional status in DHS and MICS surveys.

### 2.1 Child health and nutrition indicators

The outcomes included full immunization coverage in children aged 12–23 months and stunting prevalence in under-fives. Full immunization is defined as the proportion of children who had received, at the time of the survey, the following vaccines recommended for the first year of life: three doses of DPT, three doses of polio, one dose of measles and one dose of BCG (World Health Organization, 2019). Full immunization and stunting were defined at the level of individual children; when more than one child was present in a household, all were included. According to World Health Organization (WHO) reccomendations (WHO, 2019), children with missing information on immunizations were treated as unvaccinated. Stunting prevalence is defined as the proportion of children with height-for-age z-scores below -2 Z scores relative to the median of the 2006 WHO Child Growth Standards (WHO, 2006). Children with missing values for height or for age were excluded from the stunting analyses, as were those with extreme Z-score values, as recommended by WHO (WHO, 2006). Published multicountry analyses of missing anthropometric data in national surveys has shown that missingness is unlikely to bias the study of associations with postulated risk factors (Finaret & Hutchinson, 2018).

### 2.2 Household headship

Households were initially classified into female or male headed households, based upon list of household members provided by the questionnaire respondent to the following questions: *“Please tell me the name of each person who usually lives here, starting with the head of the household”.* The main interest is to compare households headed by females with those headed by males. It should be noted that the respondent may or may not be the head of household, and that there is a degree of subjectivity in the answer. Our household composition typology started with a process that identified 16 different FHH groups (Saad et al., submitted), which were divided according to the presence of husbands, other adult males, and children in the household, and also on whether the female head is married but the husband lives elsewhere. Because the present analyses are limited to households with children, after examining the frequencies of different types of households in most countries, we reduced the 16 FHH groups to two, depending on whether or not at least one adult male (18 years or older) lived in the household. Thus, our analyses compared three types of households: a) MHH; b) FHH with at least one adult male; and c) FHH without an adult male. To assess the presence of an adult male in FHH we used the household members list, which provides data on sex and age of all members.

### 2.3 Covariates

Our analyses also included the following additional variables: wealth index, education of the child’s mother (none/primary/secondary or more) and area of residence (urban/rural). The wealth index is calculated from a list of items such as household assets, characteristics of the building materials, availability of electricity, type of water supply and sanitary facilities, among other variables that reflect socioeconomic position of the family (Filmer & Pritchett, 2001; Rutstein & Johnson, 2004). Separate principal component analyses are carried out within urban and rural areas that are later combined into a single score using a scaling procedure to allow comparability between urban and rural households (Rutstein, 2008). For countries with information regarding polygyny, this variable was used in the sensitivity analyses. The child’s mother was classified as being in a polygynous relationship when she reported that her husband had other wives.

### 2.4 Statistical analyses

The distribution of FHH for each country was presented a world map. The distribution of household groups in each region was presented using the median proportions and interquartile ranges (Appendix). To describe how the three household groups varied according to socioeconomic position we present the percentages of MHH, FHH (any male) and FHH (no male) classified in each wealth quintile.

To test the associations between household groups and child indicators we estimated the prevalence ratio (PR) and 95% confidence interval for the two FHH categories using MHH as reference in each country using Poisson regression (Barros & Hirakata, 2003). All analyses presented in the body of the manuscript are adjusted for wealth index, mother’s education, and area of residence. Crude analyses results are presented in supplementary material. Analyses were carried out separately for each country, and later grouped into world regions using the UNICEF classification.

To assess the association of FHH with our outcomes in world regions we used for each FHH group separately meta-analysis with random effects to obtain a pooled PR estimates for region of the world and for all countries combined, with weights reflecting survey sample sizes. The random effects approach allows for heterogeneity in the true effects (Serghiou & Goodman, 2019). To measure heterogeneity, we presented the I^2^, defined as percentage of total variation across studies that is due to heterogeneity rather than chance (Higgins et al., 2003).

In addition, sensitivity analyses restricted to poorest 40% of families and including adjustment for polygynous union of child’s mother were performed to assess whether associations would differ from those observed in the full sample. These are presented in the supplementary material. The rationale for the analyses restricted to 40% poorest families was to investigate whether associations between FHH and child health might be present only among the most disadvantaged groups of the population. Regarding polygyny, sensitivity analyses assessed whether polygynous marriage could affect the allocation of resources due to the existence of other spouses.

We started the analyses with a total of 101 nationally-representative surveys carried out since 2010 that are included in the ICEH database. Of these, 91 had data on stunting, and 92 on immunization. Specific exclusions were carried out due to lack of information on the wealth index, maternal education or absence of children with the outcome in one or more FHH categories (Figure A1).

In the final sample we included 95 countries, 89 of which had information on immunization and 88 on stunting. These countries represent 90% of low income, 73% of lower-middle and 52% of upper-middle income countries in the world. For both outcomes, 40% of countries studied were from Africa.

DHS and MICs adopt two-stage sampling procedures based on strata (usually urban or rural areas) and clusters, each typically including 25–30 households. Sampling weights are calculated to compensate for over- and under-sampling by stratum and for differences in nonresponse. All analyses were carried out using statistical package Stata 16·0 (StataCorp, College Station, TX, USA), taking account the complex sampling using svy command.

Anonymized data from MICS and DHS are publicly available and the institutions responsible for these surveys were responsible for ethical clearance.

## 3 Results

### 3.1 Frequency of female-headed households

**Fig. 1:**
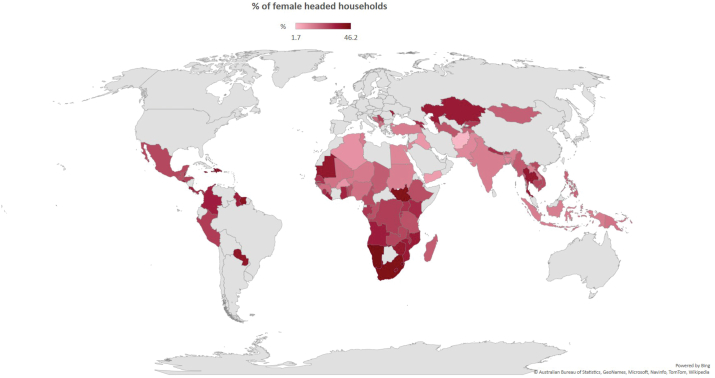
Percent of female headed households by country.

Fig. 1 presents the proportion of FHH by country. Supplementary Table A1 lists the countries, the percentages of households in each household group, and the number of children with data for the immunization (12–23 months) and stunting (0–59 months) outcomes. Countries with the lowest proportions of FHH were Afghanistan, Yemen, Iraq, State of Palestine and Burkina Faso (under 10%), whereas the largest proportions were observed in Lesotho, South Sudan, South Africa, Namibia, Maldives, Haiti, Eswatini and Jamaica (above 40%) (Fig. 1). Regarding FHH categories, Afghanistan presented the lowest proportion of FHH (any male) with 0.8% and Maldives the highest (30.6%). Regarding FHH (no male), again the lowest proportion was in Afghanistan (0.9%) and the highest proportion in Eswatini (29.2%) (Table A1). Pooled results by region are presented in Table A2. For the 95 countries analyzed, the regions with highest proportions of FHH (any male) was Latin America & Caribbean (17.6%). For FHH (no male), the highest proportions were in Eastern & Southern Africa (22.8%) Latin America & Caribbean (17.5%) and West & Central Africa (16.2%). While most regions presented showed similar proportions for both FHH (any male) and FHH (no male), Africa regions presented higher proportions of the latter group.

**Fig. 2:**
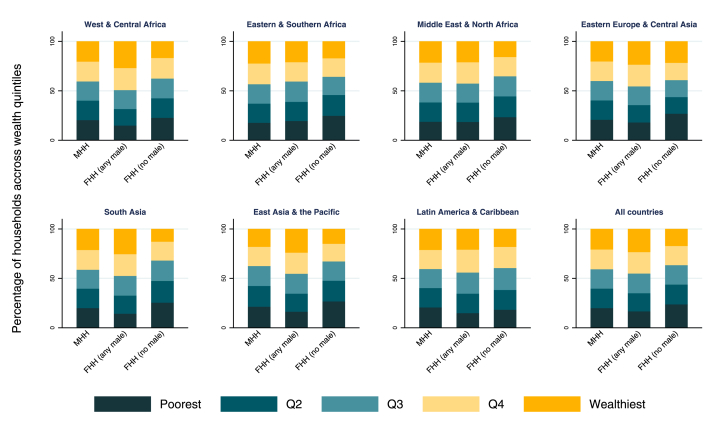
Percent of households in each quintile of wealth index according to according to sex of the head of the household, by world region and for all countries combined.

Fig. 2 shows the percentage of households in each quintile of the wealth index according to household type. Globally, FHH (no male) tended to be poorer than the other two categories: overall, the poorest quintile represented 23.8% of FHH (no male), 16.7% of FHH (any male) and 20.0% of MHH. South Asia was the region with lowest proportion of FHH (no male) in the wealthiest quintile (12.8%). FHH (any male) were generally wealthier than FHH (no male) in all regions. Compared to MHH, FHH (any male) also tended to be slightly wealthier in most regions except for Eastern & Southern Africa and Middle East & North Africa were the distribution of quintiles for FHH (any male) and MHH were very similar. Means and 95%CI for each category are presented in Table A3.

### 3.2 Full immunization

After adjustments for wealth quintiles, maternal education and area of residence, the pooled effect of FHH on full immunization for all countries was 0.99 (0.97; 1.01) for FHH (any male) and 0.99 (0.97; 1.02) for FHH (no male). In all world regions the confidence interval for the pooled PR included the unity (Fig. 3, Fig. 4). Crude and adjusted analyses produced comparable prevalence ratios and the former are presented in the appendix (Figures A2 to A15).

**Fig. 3:**
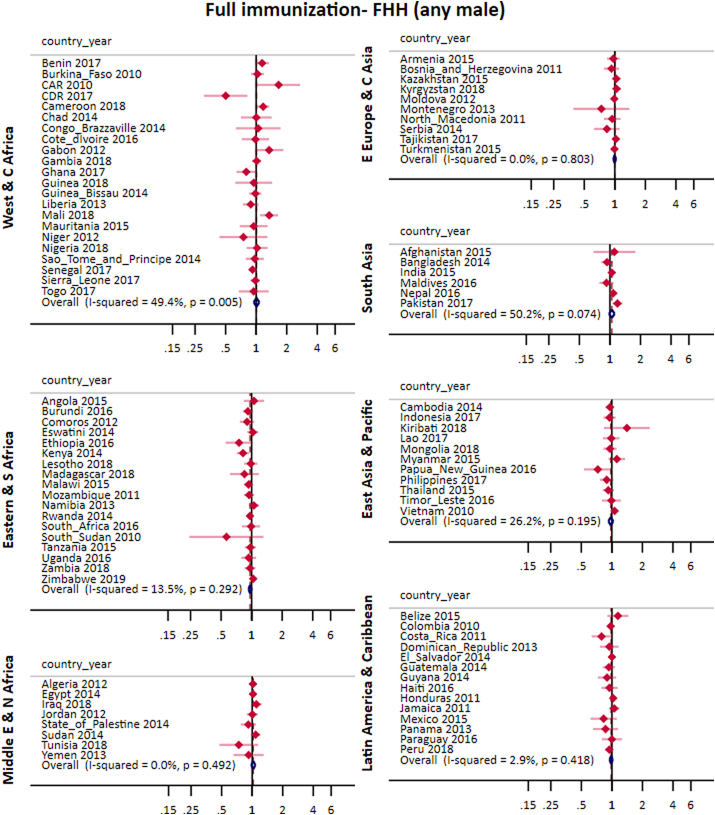
Pooled and country-specific prevalence ratios of full immunization for FHH (any male) group in comparison to MHH for each world region.

**Fig. 4:**
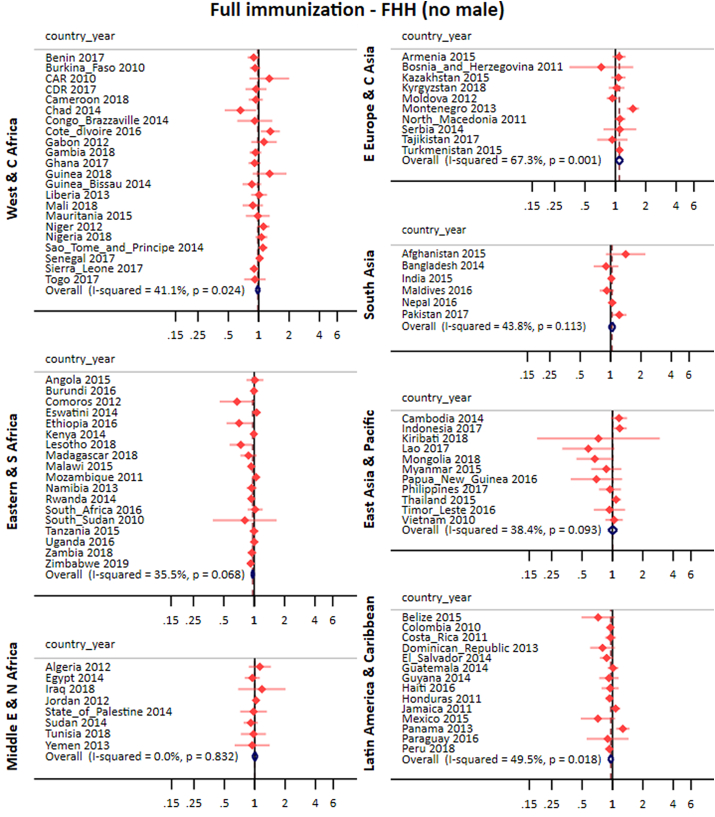
Pooled and country-specific prevalence ratios of full immunization for FHH (no male) group in comparison to MHH for each world region.

In terms of heterogeneity, the total I^2^ for FHH (any male) was 30.1% reaching around 50% in the West Africa and South Asia region. For FHH (no male), the overall I^2^ was higher (52.6%), reaching 67.3% in Easter Europe and Central Asia. The zero value for I^2^ in the Middle East & North Africa is likely due to the small proportion of FHH in this part of the world (Fig. 3, Fig. 4).

Table 1 lists the countries where the confidence intervals did not include the unity. In the Congo Democratic Republic, Costa Rica and Kenya, full immunization coverage was lower in FHH (any male) compared to MHH while the inverse association was found in Mali, Pakistan, India, Cameroon and CAR. For FHH (no male), associations were found in ten countries. Chad, Ethiopia, Lesotho and Rwanda presented lower coverage in FHH (no male) than in MHH, whereas Cote d’Ivore, Indonesia, Montenegro, Pakistan and Panama, presented higher coverage in FHH (no male) than in MHH (Fig. 3, Fig. 4 and Table 1).

**Table 1:** List of countries with statistically significant differences between MHH and the two FHH categories in full immunization and stunting.

		Full immunization		Stunting	
Country	Year	FHH (any male)	FHH (no male)	FHH (any male)	FHH (no male)
CAR	2010	1.66(1.02;1.71)			1.11(1.02;1.21)
Cameroon	2018	1.17(1.02;1.35)			
CDR	2017	0.51(0.31;0.82)			
Chad	2014		0.66(0.46;0.94)		1.16(1.05;1.29)
Costa_Rica	2011	0.79(0.63;0.99)			
Cote_dIvorire	2016		1.30(1.05;1.62)		
Egypt	2014				0.60(0.38;0.95)
Ethiopia	2016		0.73(0.51;0.93)		
Gambia	2018				0.71(0.54;0.93)
India	2015	1.04(1.01;1.08)			
Indonesia	2017		1.18(1.01;1.39)		
Kenya	2014	0.82(0.71;0.95)			
Kosovo	2013				2.69(1.35;5.38)
Lesotho	2018		0.74(0.57;0.95)		
Mali	2018	1.34(1.09;1.65)			
Montenegro	2013		1.49(1.30;1.72)	2.77(1.32;5.79)	
Nigeria	2018				0.86(0.76;0.97)
Pakistan	2017	1.19(1.06;1.33)	1.22(1.03;1.44)		
Panama	2013		1.27(1.09;1.48)		
Peru	2018				0.83(0.72;0.97)
Rwanda	2014		0.93(0.88;0.99)		
Serbia	2014			1.92(1.05;3.51)	
State_of_Palestine	2014				1.92(1.01;3.64)
Thailand	2015				0.55(0.35;0.87)
Tunisia	2018				1.70(1.05;2.78)
Turkey	2013			2.04(1.29;3.24)	
Turkmenistan	2015		1.10(1.05;1.15)		
Zambia	2018			1.16(1.02;1.33)	

### 3.3 Stunting

After adjustments, the pooled effect for FHH (any male) was 1.00 (0.98; 1.02) and for FHH (no male) was 1.00 (0.98; 1.02). Similar to results for full immunization, the crude and adjusted analyses presented similar results. Results from crude analyses are provided in the appendix (Figures A30 to A43).

**Fig. 5:**
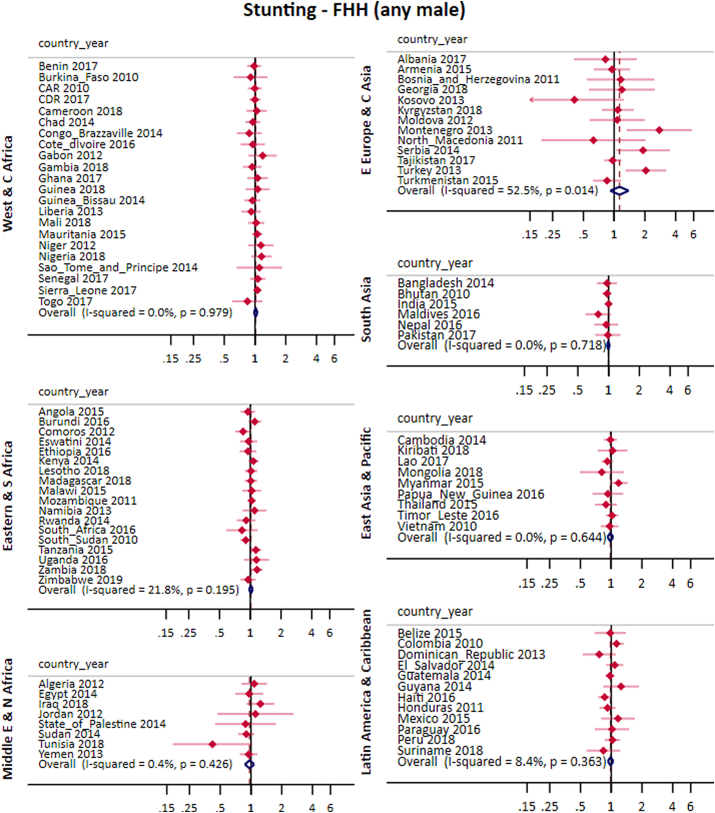
Pooled and country-specific prevalence ratios of stunting for FHH (any male) group in comparison to MHH for each world region.

**Fig. 6:**
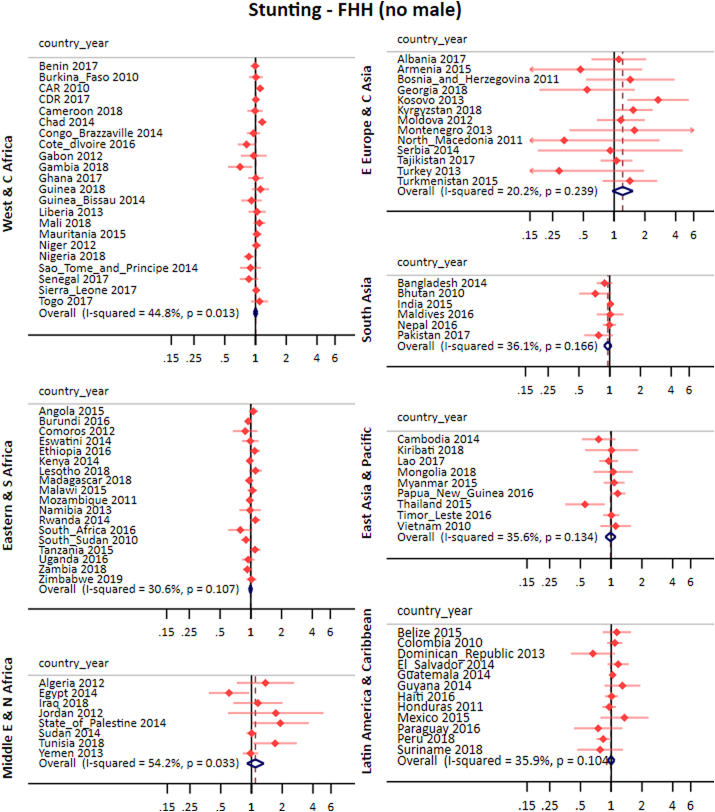
Pooled and country-specific prevalence ratios of stunting for FHH (no male) group in comparison to MHH for each world region.

Fig. 5, Fig. 6, and Table 1, present the adjusted results for stunting for each country and world region. We did not find any pattern across regions for either of the two FHH groups. Regarding heterogeneity, the overall I^2^ for FHH (any male) was 1.7%, reaching 52.5% in Eastern Europe and Central Asia. For FHH (no male), the I^2^ was 36% in the overall pooled effect, reaching 54.2% in the Middle East and North Africa (Fig. 5, Fig. 6).

Country level associations were significant in few countries. In Montenegro, Serbia, Turkey and Zambia stunting prevalence was higher in FHH (any male) compared to MHH. CAR, Chad, Kosovo, State of Palestine and Tunisia showed higher prevalence of stunting in FHH (no male) than in MHH. Another five countries presented results in the opposite direction (Egypt, Gambia, Nigeria, Peru and Thailand).

### 3.4 Sensitivity analyses

The analyses restricted to the two poorest quintiles of wealth index are presented in supplementary figures A16 to A29 for full immunization and figures A44-A57 for stunting. Supplementary Tables A4 and A5 present analyses adjusted for polygynous union of child’s mother for countries with this information.

The results from the sub-analyses restricted to the poorest wealth quintiles did not vary too from those in the main analyses, although confidence intervals tended to be wider due to smaller sample sizes. For full immunization, two countries where no association was present in the main analysis presented higher coverage in FHH (any male) compared to MHH in the sub-analyses (Burkina Faso and Kyrgyzstan). Another three countries without an association in the main analyses presented higher coverage in FHH (no male) in comparison to MHH in the sub-analyses (Uganda, Algeria and Afghanistan). Regarding stunting, only one country without an association in main analysis presented higher prevalence in FHH (any male) than MHH in the sub-analyses (Myanmar). In addition, five countries without differences in the main analysis presented statistically significant associations with FHH (no male) in the sub-analysis, four of which with higher (Yemen, Kyrgyzstan, India, Papua New Guinea) and one with lower (Dominican Republic) stunting prevalence in FHH (no male) than in MHH.

The second set of sensitivity analyses included adjustment for polygyny in 67 countries with such information. Regarding full immunization, results for FHH (any male) did not change for any country with adjustment for polygyny; for FHH (no male) the association disappeared for Rwanda but became significant in Algeria. For stunting, no changes in the associations were observed for FHH (any male). For FHH (no male), the only change was for the State of Palestine where the PR decreased slightly and the confidence interval included the unity in the new model (PR in main analysis = 1.92, 95%CI:1.01-3.64; PR in sensitivity analysis = 2.12, 95%CI:0.97-4.73).

## 4 Discussion

We investigated how two widely-used child health and nutrition indicators - stunting prevalence and full immunization coverage – vary according to the sex of household head in up to 89 countries. After detailed examination of household typologies (Saad et al., submitted) in about 100 LMICs with recent surveys, we opted to compare three types of households, MHH and FHH, the latter divided into those with or without an adult male in the home.

Our first hypothesis was that FHH would be poorer than MHH. When we compared household wealth according to the three types of households, FHH (no male) were slightly poorer than MHH, but this was not so evident for FHH (any male), suggesting that the presence of an adult male in a FHH contributes to wealth. Although there is a general indication that FHH as a whole tend to be poorer than MHH (Katapa, 2005; Muleta & Deressa, 2014; Vo et al., 2019), several authors have pointed out that FHH are not always the poorest of the poor (Bradshaw et al., 2017; Chant, 2004; Milazzo & van de Walle, 2017; Oginni et al., 2013). Women heads of households may have lower wages, but they may also receive additional support and earnings from government, community, other relatives outside the household or friends (Chant, 2004; Horrell & Krishnan, 2007; Kpoor, 2019). In addition, other characteristics such as marital status and family size may influence the wealth of FHHs. (Horrell & Krishnan, 2007; Quisumbing et al., 2001). Our analyses also showed that FHH (any male) tended to be better off than MHH in all regions, except for Eastern & Southern Africa and Middle East and North Africa. One possible explanation is that FHH (any male) may combine the external support for the woman with man’s earnings in a household with generally fewer members than MHH. However, our results should be interpreted with caution due to their cross-sectional design. In particular, empowered women who were acting as household heads and already commanding resources may be more likely to establish more recent relationships with other men. Even though the differences in wealth were not marked, we opted to adjust for household wealth, as well as maternal education and place of residence, in the analyses of child indicators.

Our second hypothesis was that differences in child health and nutrition in the crude analyses would not be marked or systematic. This is because poverty in FHH may be offset by the positive impact of higher decision-making power of women and better within-household resource allocation, giving priority to their children (Richards et al., 2013). In support of this hypothesis, earlier studies did not find any difference between 10.13039/501100005624MHH and FHH for child stunting (Adekanmbi et al., 2013; Atsu et al., 2017; Dorsey et al., 2018; Ersino et al., 2018) and full immunization (Acharya et al., 2018; Oliveira et al., 2014) in their crude analysis. Our study confirmed the absence of systematic differences between MHH and FHH in the two indicators under study, although such differences were observed in a few specific countries. When present, differences were found between MHH and FHH (no male), rather than between MHH and FHH (any male). However, the direction of significant associations varied, sometimes with children in FHH (no male) doing better, and sometimes doing worse than those from MHH.

Our third hypothesis was that, given that FHH would tend to be poorer than MHH, the adjusted analyses would show an advantage for children living in FHH. This hypothesis was rejected by our findings. Although adjustment made small differences in some countries, the overall conclusion of a lack of association was held.

Regardless of the theoretical mechanisms of association, the existing literature is scarce and reported results are as conflicting as our own. The studies identified in the literature treated FHH as a single group, without discrimination of whether or not an adult male was present. For stunting, a study from Nigeria found no association (Adekanmbi et al., 2013), whereas studies in Somalia and Pakistan (Punjab) found 13% and 25% (respectively) lower prevalence in FHH (Khalid & Martin, 2017; Kinyoki et al., 2016) and another in Tanzania found a 16% higher prevalence in such households (Sunguya et al., 2019). The authors from the latter study argue that FHH are becoming more common over time, as is the educational level of mothers; they suggest that mothers who work or study may spend less time caring for their children, which would increase the risk of stunting independently of income level. In comparison with these published results, some of our findings were divergent. For example, for Nigeria, we found lower stunting prevalence in FHH (no male), while for Pakistan and Tanzania we did not find significant differences between MHH and FHH groups. The association in Nigeria was only found in FHH (no male) suggesting that the combined FHH category used in the published study is heterogeneous and our more granular typology revealed differences in comparison to MHH. The divergence with the published Pakistan study could be due to the fact that this study was restricted to the Punjab region while our analyses were national. For Tanzania, when we use the same typology as in the published study (MHH vs. FHH), we found a similar association.

In addition, Somalia was not included in our analysis because we did not have a DHS or MICS for this country with information for the outcome since 2010.

On immunization, a WHO report on DPT immunization and coverage in 10 countries (mostly from Africa) found no difference between MHH and FHH in eight countries, but higher coverage in FHH compared to MHH in Nigeria, and lower coverage in Ethiopia (WHO, 2018) Our analyses show that in Ethiopia the FHH groups tend to be poorer than MHH, whereas in Nigeria the reverse pattern is observed. These differences in wealth distribution as well other contextual variables could explain the divergence between the results from the two countries. Although this study used data from same surveys as our analyses, it was focused on DPT and not on full immunization. Another study in West Africa found lower coverage of full immunization in FHH for five of six countries assessed (Liberia, Mali, Nigeria, Senegal, Sierra Leone, and Togo). The authors also highlight other relevant characteristics for association such as polygyny, mother education and marital status (Akinyemi et al., 2017). This last study used same set of vaccines as in our analyses of full immunization. Their results were similar to ours for five countries, but they failed to find differences between MHH and FHH in Mali, while we found lower coverage in FHH (any male). Our comparison with the literature reinforces the need for using more granular FHH typologies in future studies.

Regarding regional findings, our analyses did not disclose any regional patterns of systematic differences between FHH and MHH. Sensitivity analyses adjusting for polygynous union of the child’s mother and those restricted for 40% poorest households did not lead to any noticeable change in our results. In general, heterogeneity was moderate to high due to variability in results from country to country, possibly linked to contextual, social and cultural characteristics of each country. Another possible reason for heterogeneity is related to the sizes of FHH groups in national samples, which ranged from less than 1%–30%, with small groups in some countries leading to random variability and heterogeneity.

The finding that associations were present in some countries raises the necessity of addressing a key topic in FHH research: the household head definition. This concept may vary according to norms, culture and beliefs in a specific country. In some cultures, the person defined as head of household is not necessarily the same person who has the highest income or decision-making power in the family (Budlender, 2003; Posel, 2001; Rogan, 2016). For example, in some African countries, the oldest resident – such as, the widowed grandmother of the child - is often classified as head of household by other members, but this does not always reflect being in charge of resource allocation or of routine decisions within the household (Rogan, 2016). Other relevant points about cultural and social influences in household head status are relevant. For example, where labor migration is common, the woman may be defined as the head of household although the ties with the husband remain through financial and material provisions. On other hand, absence of the husband and the pressure for women to become the head may result in economic, social, emotional and relational stress for their families (Fleischer, 2007; Yabiku et al., 2012) In addition, in some societies poor women who are single mothers and heads of household may become involved with men married to other women, from whom they receive financial support to provide a safety net for themselves and for their children (Leclerc-Madlala, 2002; Swidler & Watkins, 2007). Differences among countries in conceptualization and definition of household heads may generate misclassification of FHH and lack of consistency in analyses such as ours where results were pooled across several countries. We also need to highlight that these discrepancies may be present within countries with variations according to sub-national regions and ethnicity, for example. Thus, the interpretation of associations of FHH with health outcomes should be done with due caution.

This study presents additional limitations. At first, FHH households were stratified according to presence or absence of a usual resident adult man in household. Although other possible FHH typologies exist (Saad, submitted), we opted for this distinction based on male presence in household because an adult man may not only increase family income but also the affect culturally and gender-defined opportunities for the family (e.g. in some countries woman have difficulty to maintaining rights to land inherited when the family have any man) (Budlender, 2003). Our initial exploratory analyses of FHH types (Saad, submitted) showed that more granular characterization of household headship resulted in low prevalence of some FHH groups in several countries, whereas the present typology provides reasonable sample sizes allowing for the proposed analyses. Last but not least, our typology was derived using information routinely collected by DHS and MICS surveys, which are not tailored specifically for FHH analyses. Headship information in these surveys is subject to the respondent's interpretation and consequent potential misclassification. Furthermore, not all country surveys had data on our outcomes of interest, and, in many surveys, no information was available regarding relevant variables such as whether the woman was married, whether migrant male labor explained the temporary absence of the husband or partner, or on for how long the woman had been the head. For example, the child’s mother who is a head of household her may not have been in this position during the whole period when her children were aged under five years. Generally speaking, misclassification would tend to reduce the magnitude of true associations. More information on contextual variables regarding FHH status in each country may help define more granular household typologies which would reveal associations that we were unable to identify in the present multi-country analyses.

Among the strengths of our study is the fact that 95 countries were included, whereas earlier publications on this issue covered 10 or fewer countries (Adekanmbi et al., 2013; Basant Kumar Panda & Mishra, 2020; Ersino et al., 2018; Geweniger & Abbas, 2020; Akinyemi et al., 2017). We were able to include 90% of all low-income and 70% of lower-middle-income countries of the world. Other strengths include the use of standardized definitions, both the child outcomes as well as for household headship. We tried to go beyond a simple MHH versus FHH comparison by also accounting for the presence of adult males in FHH. This allowed us to investigate heterogeneity in child wellbeing that may have been invisible in earlier studies in which a single FHH group was studied. As we can see in economic analysis FHH (any male) were slightly wealthier than MHH, while FHH (no male) were slightly poorer than MHH. This reinforces the notion that FHH are not homogeneous, a concept that should be considered in future analyses. Further studies are also needed to understand more about the dynamics of FHH – for example, how these households compensate for not having a male head as the main breadwinner, e.g. possibly by taking male adolescents out of school to join the labor market.

In terms of implications for policies, our study suggests that although differences in child outcomes between FHH and MHH were not present in most countries, in some countries sex of the household head may influence child health. Depending on the country and context, living in a FHH may either improve or worsen child health and nutrition conditions. We recommend that existing survey data should be stratified according to FHH and MHH categories, and results should be fed back to decision makers at country level. Interventions with a focus on vulnerable groups should recognize the diversity of FHH rather than treat them as a single, potentially vulnerable category.

## 5 Conclusions

Summing up, in most countries there were no differences either in stunting prevalence or in full immunization according to household headship, even after accounting for family wealth, education and residence. A few countries presented inequalities with different directions of association, indicating the diversity of FHH and how complex the meaning and measurement of this group may be. In these countries, further research is warranted to understand contextual variables, examine mediating factors, and exploring alternative definitions of household headship.

## Funding

This work was funded by grants from the 10.13039/501100000193International Development Research Centre, 10.13039/100000865Bill & Melinda Gates Foundation, 10.13039/100010269Wellcome Trust and 10.13039/501100012418ABRASCO (Associacao Brasileira de Saude Coletiva). The views expressed herein do not necessarily represent those of IDRC or its Board of Governors.

## Ethical statement

Anonymized data from MICS and DHS are publicly available and the institutions responsible for these surveys were responsible for ethical clearance.

## Author statement

Andrea Wendt and Franciele Hellwig: Software, Formal analysis, Data Curation, Writing, Visualization, Cesar Victora and Aluisio J D Barros: Conceptualization, Resources, Writing, Supervision, Project administration, Funding acquisition, Ghada E Saad, Cheikh Faye, Zitha Mokomane and Ties Boerma: Writing, Supervision.

## Declaration of competing interest

The authors state that there are no competing interests.
