# Supplementary material for: Are children in female-headed households at a disadvantage? An analysis of immunization coverage and stunting prevalence: in 95 low- and middle-income countries
Source: SSM Popul Health. 2021 Aug 5;15:100888. doi: 10.1016/j.ssmph.2021.100888 (PMC8369002; doi:10.1016/j.ssmph.2021.100888)
Supplement: Multimedia component 1 [file mmc1.docx]

APPENDIX

Supplementary material


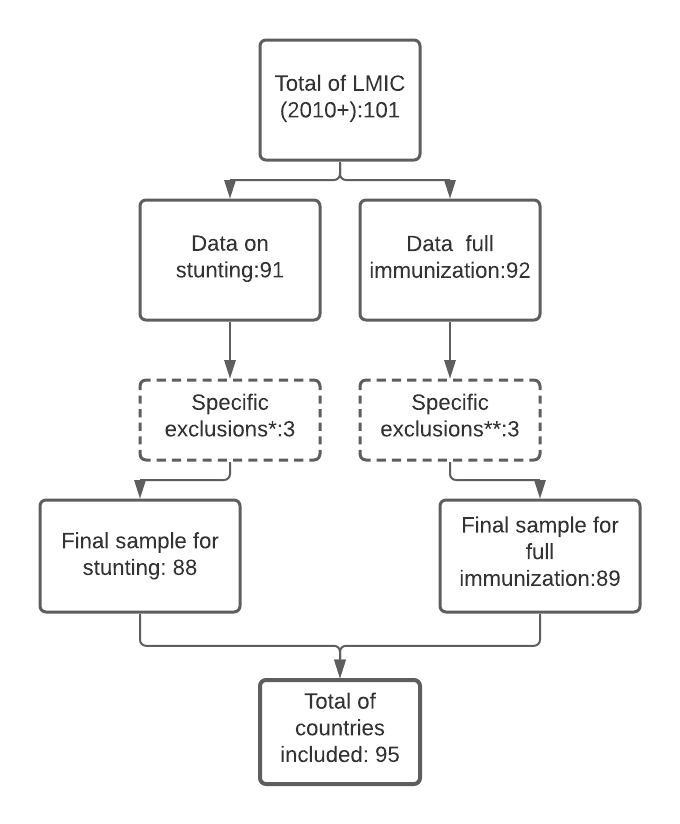


**Figure A1. Survey selection flowchart**

**Exclusions from analyses of stunting: Barbados, Kazakhstan and Saint Lucia*

***Exclusions from analyses of full immunization: Cuba, Kosovo and Ukraine*

**Table A1. Countries included in article, year of survey, percentage of female headed household and number of children with information for stunting and full immunization.**

| **Country** | **Year** | **FHH (any male)** | **FHH (no male)** | **N of children with information for stunting** | **N of children with information for full immunization** |
| --- | --- | --- | --- | --- | --- |
| Afghanistan | 2015 | 0.8% | 0.9% |  | 5772 |
| Albania | 2017 | 6.2% | 11.1% | 2578 |  |
| Algeria | 2012 | 7.6% | 2.9% | 13726 | 2832 |
| Angola | 2015 | 11.4% | 23.1% | 7601 | 2819 |
| Armenia | 2015 | 15.8% | 17.3% | 1585 | 343 |
| Bangladesh | 2014 | 3.9% | 8.6% | 6636 | 1421 |
| Belize | 2015 | 15.5% | 17.5% | 2418 | 503 |
| Benin | 2017 | 6.6% | 18.3% | 13065 | 2477 |
| Bhutan | 2010 | 21.1% | 6.8% | 5838 |  |
| Bosnia_and_Herzegovina | 2011 | 7.9% | 11.0% | 2153 | 516 |
| Burkina_Faso | 2010 | 2.5% | 7.4% | 6891 | 2760 |
| Burundi | 2016 | 6.7% | 22.0% | 6336 | 2578 |
| CAR | 2010 | 5.7% | 16.2% | 10243 | 2109 |
| Cambodia | 2014 | 17.1% | 9.7% | 4869 | 1425 |
| Cameroon | 2018 | 8.4% | 17.6% | 4930 | 1726 |
| Chad | 2014 | 5.9% | 16.2% | 10947 | 2849 |
| Colombia | 2010 | 17.3% | 16.7% | 17824 | 3435 |
| Comoros | 2012 | 25.8% | 13.4% | 2862 | 627 |
| Congo_Brazzaville | 2014 | 6.5% | 18.3% | 8729 | 1773 |
| Congo_Democratic_Republic | 2017 | 8.2% | 20.3% | 20885 | 4251 |
| Costa_Rica | 2011 | 20.5% | 16.0% |  | 449 |
| Cote_dIvoire | 2016 | 4.8% | 12.7% | 8949 | 1784 |
| Dominican_Republic | 2013 | 21.2% | 18.7% | 3573 | 691 |
| Egypt | 2014 | 5.2% | 7.8% | 13956 | 3036 |
| El_Salvador | 2014 | 18.6% | 16.0% | 7225 | 1504 |
| Eswatini | 2014 | 16.4% | 29.2% | 2638 | 540 |
| Ethiopia | 2016 | 8.2% | 17.2% | 9542 | 1896 |
| Gabon | 2012 | 10.0% | 20.0% | 4090 | 1167 |
| Gambia | 2018 | 10.4% | 11.0% | 9718 | 1895 |
| Georgia | 2018 | 15.9% | 16.1% | 2022 |  |
| Ghana | 2017 | 10.2% | 23.2% | 8797 | 1681 |
| Guatemala | 2014 | 9.8% | 15.3% | 12120 | 2371 |
| Guinea | 2018 | 7.0% | 11.7% | 4005 | 1392 |
| Guinea_Bissau | 2014 | 12.6% | 10.1% | 7523 | 1591 |
| Guyana | 2014 | 17.6% | 16.8% | 2997 | 688 |
| Haiti | 2016 | 23.9% | 21.1% | 6680 | 1174 |
| Honduras | 2011 | 13.3% | 14.7% | 10626 | 2206 |
| India | 2015 | 7.5% | 7.1% | 228392 | 46583 |
| Indonesia | 2017 | 6.0% | 8.9% |  | 3407 |
| Iraq | 2018 | 6.0% | 3.0% | 16355 | 3205 |
| Jamaica | 2011 | 23.0% | 23.2% |  | 314 |
| Jordan | 2012 | 6.3% | 6.6% | 6181 | 1940 |
| Kazakhstan | 2015 | 17.9% | 18.1% |  | 1103 |
| Kenya | 2014 | 8.8% | 23.4% | 20394 | 3951 |
| Kiribati | 2018 | 20.2% | 6.3% | 2143 | 453 |
| Kosovo | 2013 | 8.9% | 4.8% | 1510 |  |
| Kyrgyzstan | 2018 | 13.1% | 16.2% | 3436 | 643 |
| Lao | 2017 | 7.6% | 6.4% | 11346 | 2215 |
| Lesotho | 2018 | 18.1% | 23.0% | 3141 | 667 |
| Liberia | 2013 | 19.4% | 15.8% | 3817 | 1402 |
| Madagascar | 2018 | 7.3% | 14.9% | 12444 | 2590 |
| Malawi | 2015 | 9.1% | 21.6% | 5704 | 3227 |
| Maldives | 2016 | 30.6% | 13.7% | 2509 | 585 |
| Mali | 2018 | 6.0% | 11.4% | 9476 | 1940 |
| Mauritania | 2015 | 15.4% | 22.3% | 10058 | 2131 |
| Mexico | 2015 | 13.2% | 13.3% | 7855 | 1536 |
| Moldova | 2012 | 10.7% | 25.3% | 1669 | 386 |
| Mongolia | 2018 | 8.2% | 13.3% | 5932 | 1077 |
| Montenegro | 2013 | 7.7% | 13.6% | 1366 | 266 |
| Mozambique | 2011 | 11.6% | 23.9% | 10400 | 2197 |
| Myanmar | 2015 | 10.3% | 12.2% | 4537 | 891 |
| Namibia | 2013 | 18.5% | 25.5% | 2603 | 963 |
| Nepal | 2016 | 9.3% | 22.0% | 2288 | 952 |
| Niger | 2012 | 2.4% | 13.5% | 5336 | 2145 |
| Nigeria | 2018 | 3.8% | 14.2% | 12198 | 5982 |
| North_Macedonia | 2011 | 7.7% | 8.3% | 1317 | 282 |
| Pakistan | 2017 | 5.5% | 6.9% | 3396 | 1844 |
| Panama | 2013 | 16.9% | 15.0% |  | 1323 |
| Papua_New_Guinea | 2016 | 7.0% | 10.4% | 4094 | 1792 |
| Paraguay | 2016 | 26.2% | 12.5% | 4419 | 1012 |
| Peru | 2018 | 13.2% | 15.5% | 22188 | 4403 |
| Philippines | 2017 | 11.0% | 9.7% |  | 1958 |
| Rwanda | 2014 | 9.1% | 21.9% | 3787 | 1516 |
| Sao_Tome_and_Principe | 2014 | 8.5% | 26.3% | 1944 | 391 |
| Senegal | 2017 | 17.5% | 12.8% | 11696 | 2312 |
| Serbia | 2014 | 13.0% | 15.5% | 2406 | 524 |
| Sierra_Leone | 2017 | 13.6% | 17.7% | 11399 | 2289 |
| South_Africa | 2016 | 17.0% | 25.6% | 1451 | 643 |
| South_Sudan | 2010 | 19.3% | 22.7% | 6073 | 1683 |
| State_of_Palestine | 2014 | 3.4% | 5.8% | 6939 | 1538 |
| Sudan | 2014 | 6.0% | 8.2% | 12422 | 2641 |
| Suriname | 2018 | 25.2% | 15.4% | 3390 |  |
| Tajikistan | 2017 | 13.6% | 7.2% | 5864 | 1259 |
| Tanzania | 2015 | 8.4% | 16.1% | 9820 | 2067 |
| Thailand | 2015 | 22.2% | 15.0% | 11208 | 2500 |
| Timor_Leste | 2016 | 7.3% | 10.2% | 6612 | 1410 |
| Togo | 2017 | 6.8% | 20.5% | 4908 | 973 |
| Tunisia | 2018 | 6.6% | 8.9% | 3287 | 656 |
| Turkey | 2013 | 6.6% | 8.3% | 2688 |  |
| Turkmenistan | 2015 | 16.7% | 7.3% | 3715 | 787 |
| Uganda | 2016 | 8.2% | 22.8% | 5137 | 2826 |
| Vietnam | 2010 | 16.0% | 10.2% | 3563 | 760 |
| Yemen | 2013 | 3.4% | 4.4% | 14137 | 2999 |
| Zambia | 2018 | 8.7% | 18.1% | 9446 | 1880 |
| Zimbabwe | 2019 | 11.5% | 26.3% | 5965 | 1153 |

**Table A2. Distribution of household groups and N of countries with information for child outcomes for each region.**

| **Region** | **MHH**  **% (IQ interval)** | **FHH (any male)**  **% (IQ interval)** | **FHH (no male)**  **% (IQ interval)** | **Number of countries with information** | | |
| --- | --- | --- | --- | --- | --- | --- |
|  |  |  |  | **Immunization** | **Stunting** | |
|  | P<0.001 | P<0.001 | P<0.001 |  | |  |
| **West & Central Africa** | 75.1 (69.7 – 81.3) | 7.6 (5.9 – 10.4) | 16.2 (12.7 – 20.0) | 22 | | 22 |
| **Eastern & Southern Africa** | 66.7 (58.9 – 71.3) | 10.2 (8.4 – 17.0) | 22.8 (18.1 – 23.9) | 18 | | 18 |
| **Middle East & North Africa** | 88.3 (86.4 – 90.9) | 6.0 (4.3 – 6.5) | 6.2 (3.7 – 8.0) | 8 | | 8 |
| **Eastern Europe & Central Asia** | 77.4 (68.0 – 82.7) | 11.8 (7.7 – 15.8) | 12.4 (8.3 – 16.2) | 10 | | 13 |
| **South Asia** | 85.4 (68.7 – 87.5) | 7.5 (3.9 – 21.1) | 7.1 (6.8 – 13.7) | 6 | | 6 |
| **East Asia & the Pacific** | 78.5 (73.5 – 85.5) | 10.3 (7.3 – 17.1) | 10.2 (8.9 – 12.2) | 11 | | 9 |
| **Latin America & Caribbean** | 65.6 (60.1 – 71.3) | 17.6 (13.3 – 23.0) | 17.5 (15.0 – 17.5) | 14 | | 12 |

**Table A3. Quintiles distribution for all countries and world regions according to household groups**

|  | **MHH** | **FHH (any male)** | **FHH (no male)** |
| --- | --- | --- | --- |
| **West Africa** |  |  |  |
| Q1 | 20.4 (19.7; 21.2) | 15.0 (12.9; 17.1) | 22.7 (18.7; 26.8) |
| Q2 | 19.7 (19.1; 20.4) | 16.7 (15.4; 18.0) | 19.7 (18.2; 21.3) |
| Q3 | 19.4 (19.0; 19.9) | 19.2 (17.3; 21.0) | 20.0 (18.0; 22.0) |
| Q4 | 20.0 (19.4; 20.6) | 22.2 (20.4; 22.6) | 20.8 (19.0; 22.6) |
| Q5 | 20.4 (19.4; 21.3) | 26.9 (23.6; 30.2) | 16.7 (14.4; 19.1) |
| **Eastern & Southern Africa** |  |  |  |
| Q1 | 17.8 (16.8; 18.7) | 19.6 (18.3; 20.8) | 24.8 (21.8; 27.9) |
| Q2 | 19.3 (18.5; 20.1) | 19.3 (18.2; 20.5) | 21.1 (19.5; 22.6) |
| Q3 | 19.7 (19.2; 20.1) | 20.6 (19.8; 21.5) | 18.4 (17.2; 19.6) |
| Q4 | 20.9 (19.9; 22.0) | 19.5 (18.4; 20.7) | 18.6 (16.7; 20.6) |
| Q5 | 22.3 (21.0; 23.5) | 21.0 (18.7; 23.2) | 17.0 (14.2; 19.8) |
| **Middle East & North Africa** |  |  |  |
| Q1 | 18.8 (17.5; 20.0) | 18.6 (15.7; 21.5) | 23.4 (19.0; 27.9) |
| Q2 | 19.6 (18.9; 20.3) | 19.6 (16.5; 22.6) | 21.0 (20.1; 21.9) |
| Q3 | 20.0 (19.5; 20.5) | 19.3 (17.0; 21.5) | 20.3 (16.6; 24.0) |
| Q4 | 20.3 (19.4; 21.1) | 21.6 (18.7; 24.5) | 19.5 (16.8; 22.2) |
| Q5 | 21.4 (20.6; 22.2) | 21.0 (17.4; 24.6) | 15.7 (13.0; 18.5) |
| **Easter Europe & Central Asia** |  |  |  |
| Q1 | 20.8 (19.9; 21.7) | 18.1 (16.7; 19.6) | 26.9 (19.9; 33.8) |
| Q2 | 19.6 (19.2; 20.0) | 17.6 (16.0; 19.1) | 16.8 (12.8; 20.9) |
| Q3 | 19.6 (19.3; 20.0) | 18.9 (17.6; 20.2) | 17.2 (12.9; 21.5) |
| Q4 | 19.8 (19.3; 20.2) | 21.9 (20.4; 23.5) | 17.4 (13.2; 21.6) |
| Q5 | 20.2 (19.4; 21.0) | 23.5 (20.4; 26.5) | 21.7 (11.9; 21.4) |
| **South Asia** |  |  |  |
| Q1 | 20.0 (19.2; 20.8) | 14.4 (9.7; 19.0) | 25.5 (18.1; 32.9) |
| Q2 | 19.7 (18.9; 20.5) | 18.3 (15.5; 21.1) | 21.9 (18.9; 25.0) |
| Q3 | 19.1 (18.5; 19.7) | 19.9 (17.0; 22.8) | 20.7(18.5; 22.8) |
| Q4 | 20.0 (19.0; 21.0) | 22.1 (17.6; 26.5) | 19.1 (14.3; 23.9) |
| Q5 | 21.2 (20.4; 22.0) | 25.4 (17.6; 33.1) | 12.8 (9.8; 15.8) |
| **East Asia & the Pacific** |  |  |  |
| Q1 | 21.4 (20.2; 22.7) | 16.4 (13.6; 19.2) | 26.8 (22.3; 31.2) |
| Q2 | 21.0 (20.1; 21.9) | 18.3 (16.6; 20.0) | 21.0 (19.4; 22.5) |
| Q3 | 20.1 (19.7; 20.6) | 20.2 (18.9; 21.5) | 19.5 (17.6; 21.5) |
| Q4 | 19.4 (18.6; 20.2) | 21.5 (20.0; 22.7) | 17.9 (15.7; 20.0) |
| Q5 | 18.0 (16.9; 19.1) | 23.9 (20.8; 26.9) | 14.9 (12.2; 17.6) |
| **Latin America & Caribbean** |  |  |  |
| Q1 | 20.7 (19.2; 22.2) | 14.9 (13.3; 16.6) | 18.4 (16.0; 20.7) |
| Q2 | 19.6 (19.1; 20.2) | 19.7 (18.6; 20.9) | 20.0 (18.9; 21.1) |
| Q3 | 19.2 (18.7; 19.8) | 21.4 (20.6; 22.2) | 22.2 (20.7; 23.7) |
| Q4 | 19.3 (18.8; 19.9) | 23.1 (22.0; 24.2) | 21.4 (19.7; 23.1) |
| Q5 | 21.1(20.3; 22.0) | 20.8 (18.9; 22.8) | 18.1 (16.1; 20.0) |
| **All countries** |  |  |  |
| Q1 | 20.0 (19.5; 20.4) | 16.7 (15.8; 17.6) | 23.8(22.0; 25.5) |
| Q2 | 19.8 (19.5; 20.0) | 18.3 (17.7; 19.0) | 20.0 (19.2; 20.8) |
| Q3 | 19.6 (19.4; 19.8) | 19.9 (19.3; 20.5) | 19.7 (18.7; 20.6) |
| Q4 | 20.0 (19.7; 20.3) | 21.7 (20.9; 22.4) | 19.4 (18.4; 20.4) |
| Q5 | 20.7 (20.2; 21.2) | 23.4 (22.1; 24.6) | 17.1 (15.4; 18.8) |

#
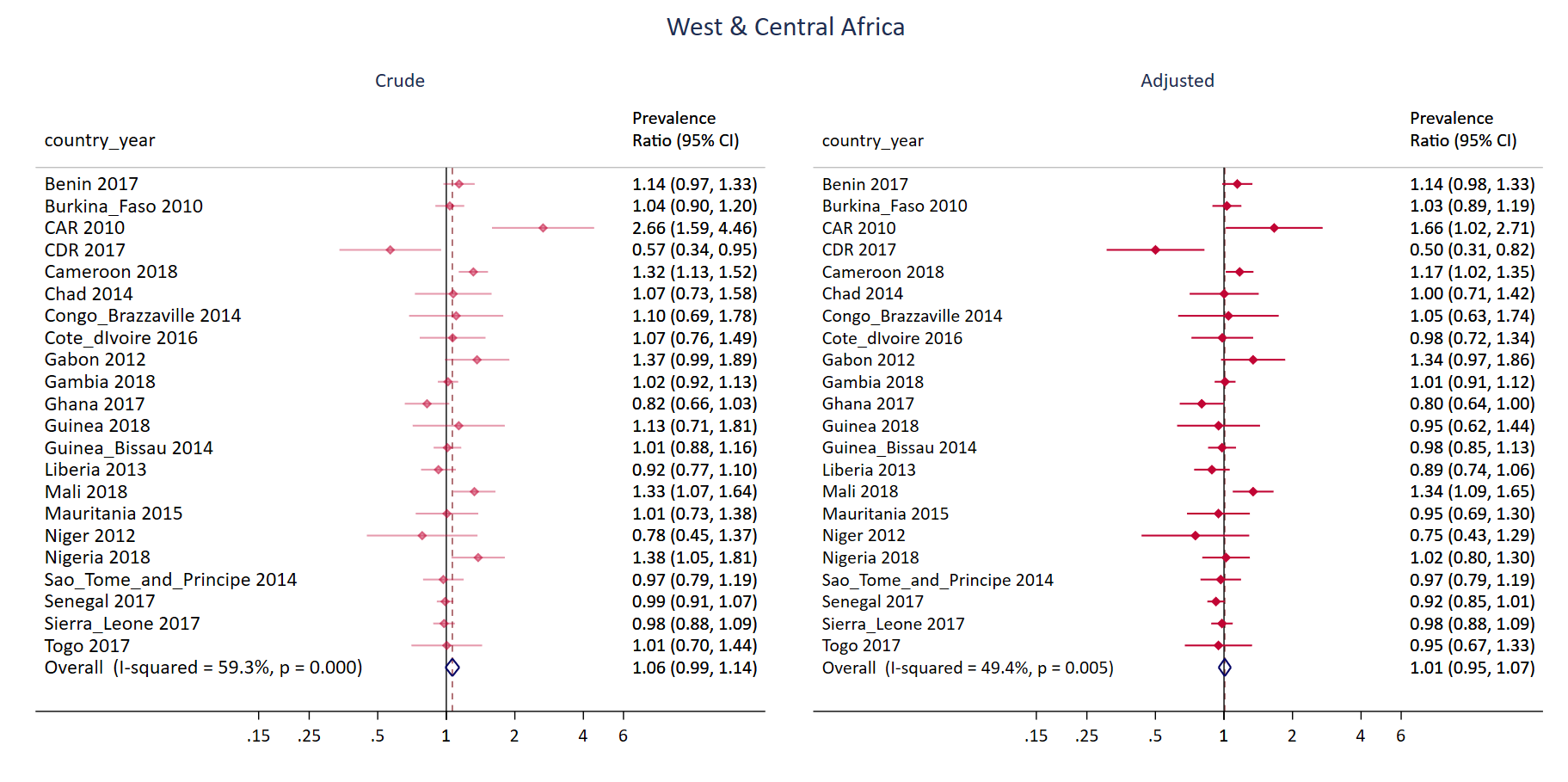


**Figure A2. Crude and adjusted prevalence ratio for full immunization in FHH (any male) in West & Central Africa**


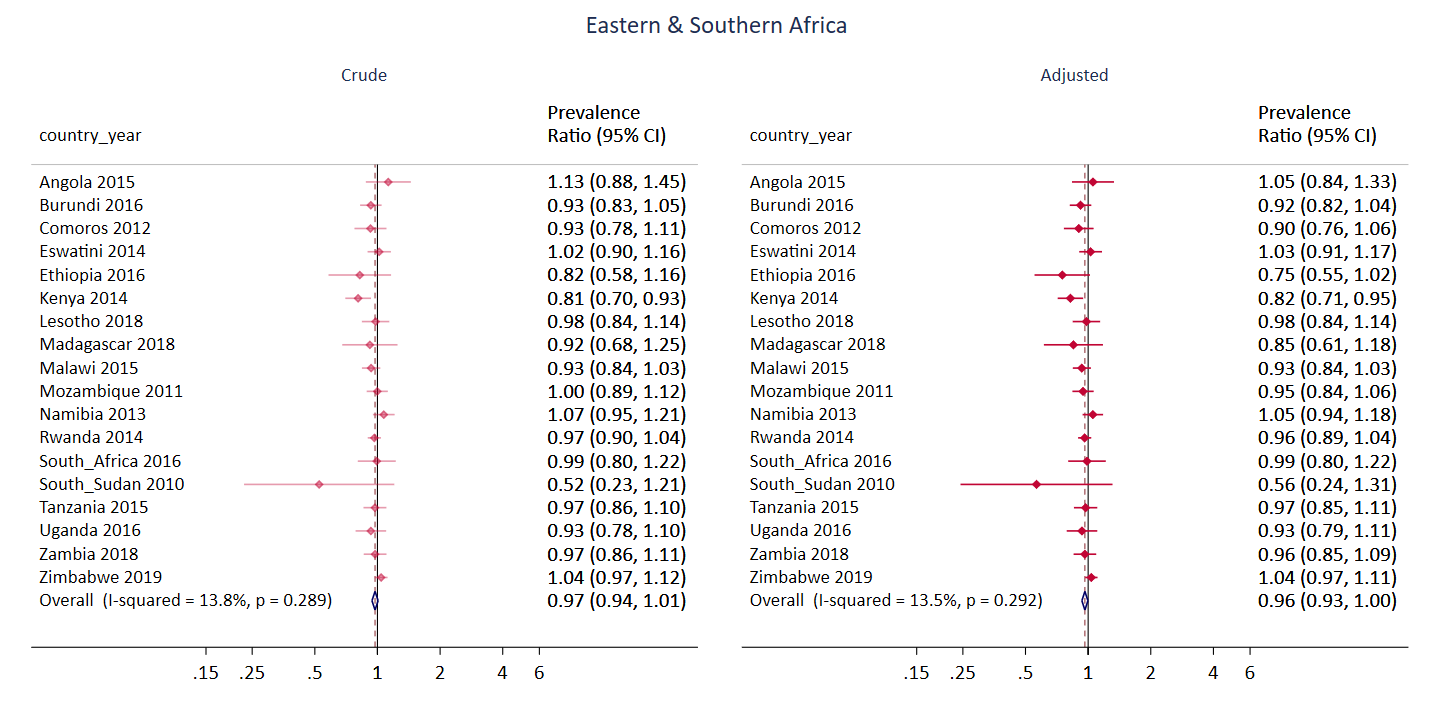


**Figure A3. Crude and adjusted prevalence ratio for full immunization in FHH (any male) in Easter & Southern Africa**


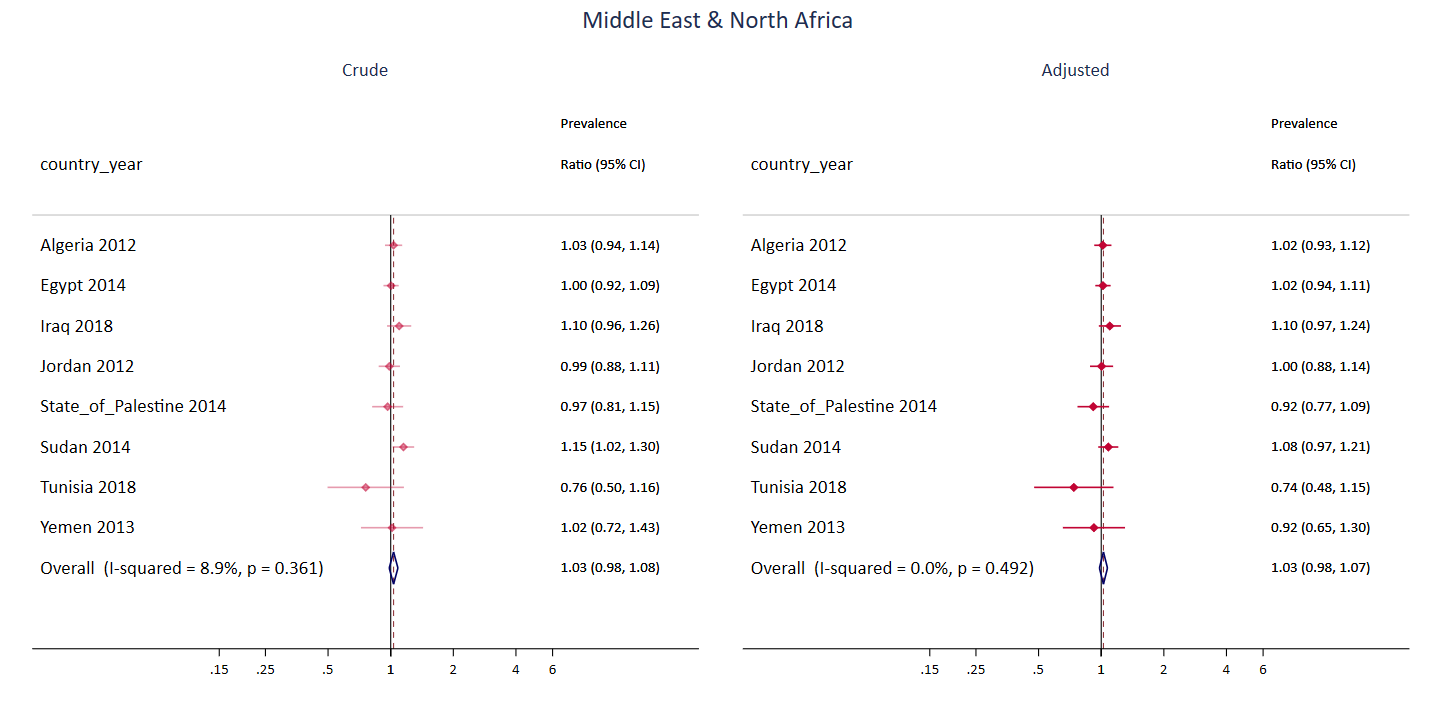


**Figure A4. Crude and adjusted prevalence ratio for full immunization in FHH (any male) in Middle East & North Africa**


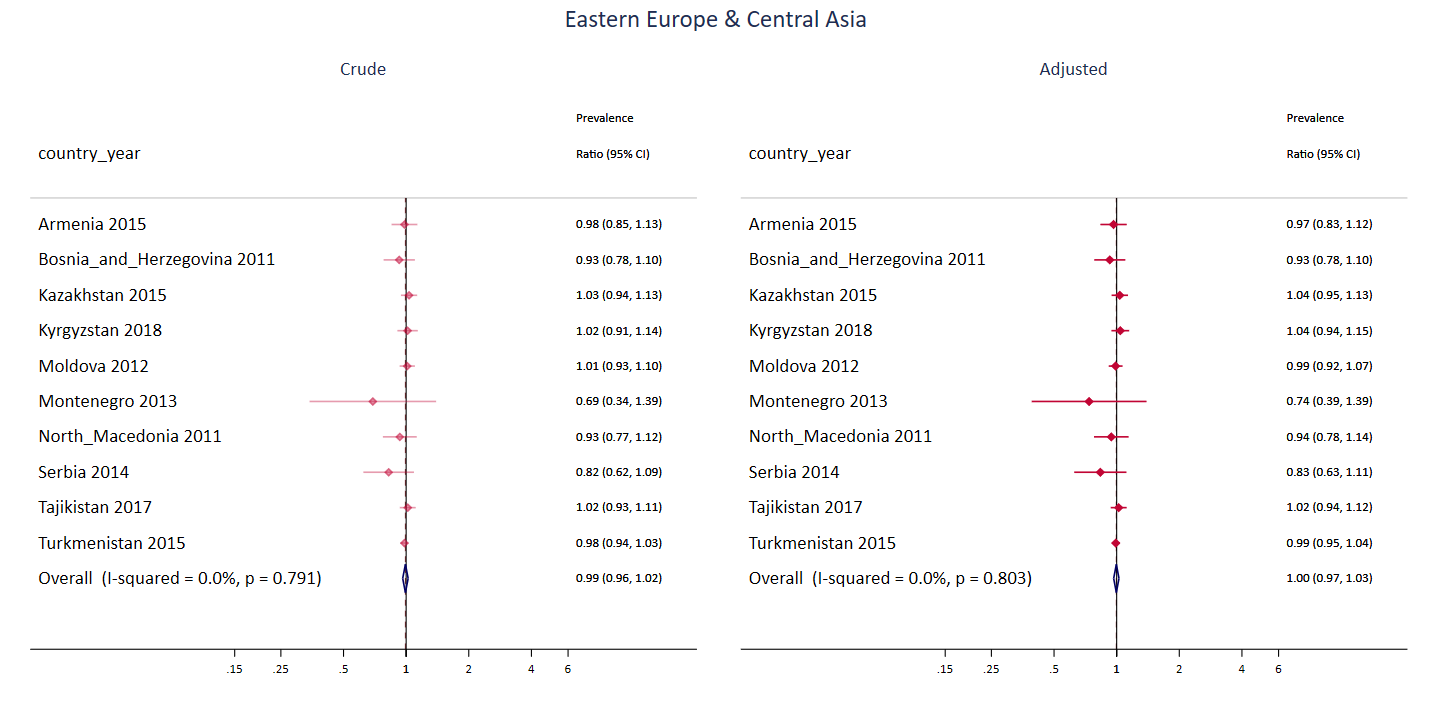


**Figure A5. Crude and adjusted prevalence ratio for full immunization in FHH (any male) in Eastern Europe & Central Asia**


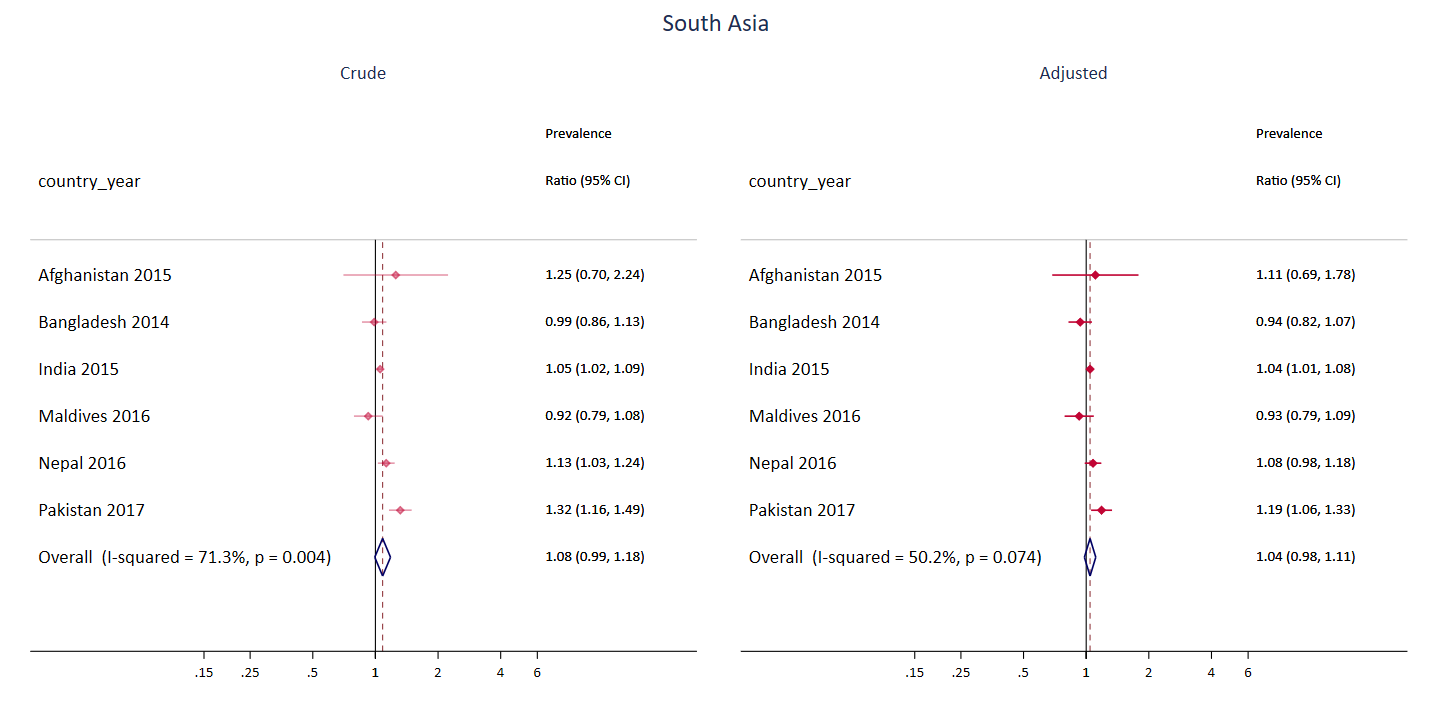


**Figure A6. Crude and adjusted prevalence ratio for full immunization in FHH (any male) in South Asia**


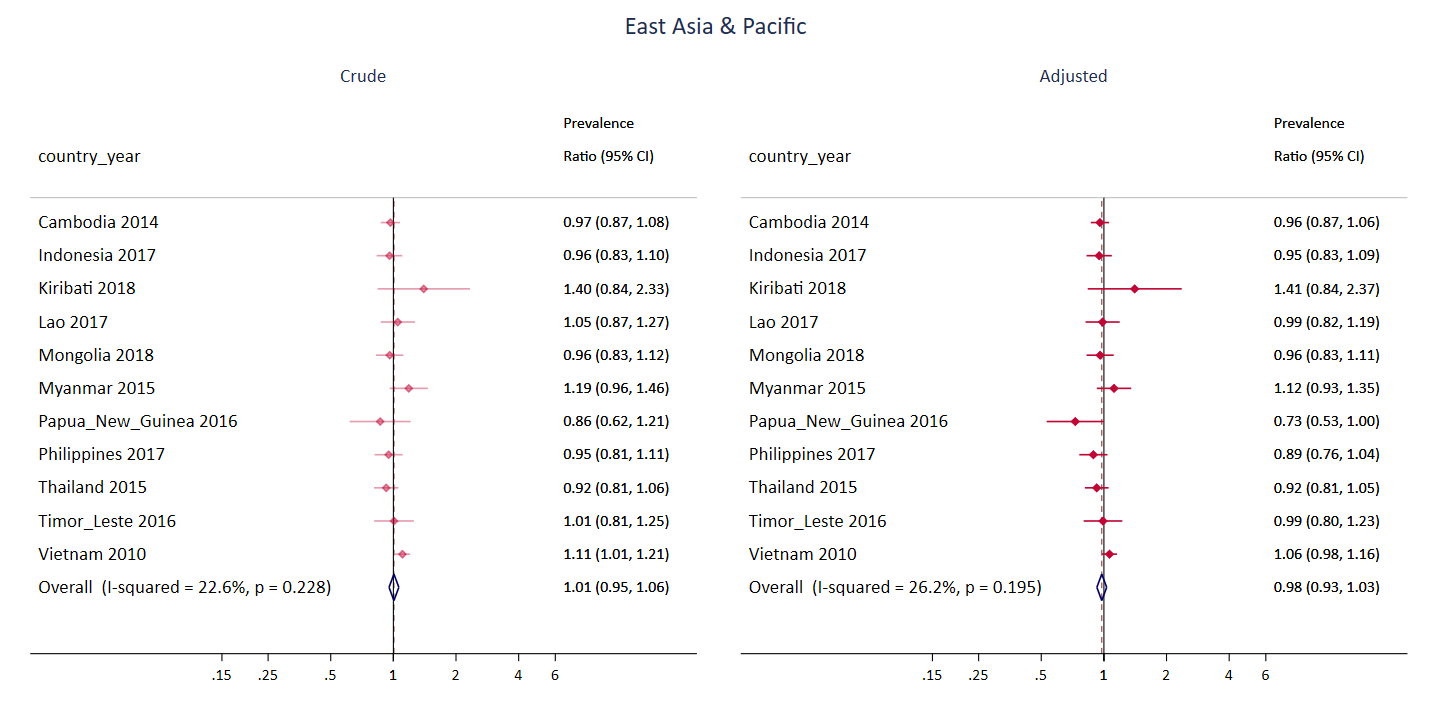


**Figure A7. Crude and adjusted prevalence ratio for full immunization in FHH (any male) in East Asia & Pacific**


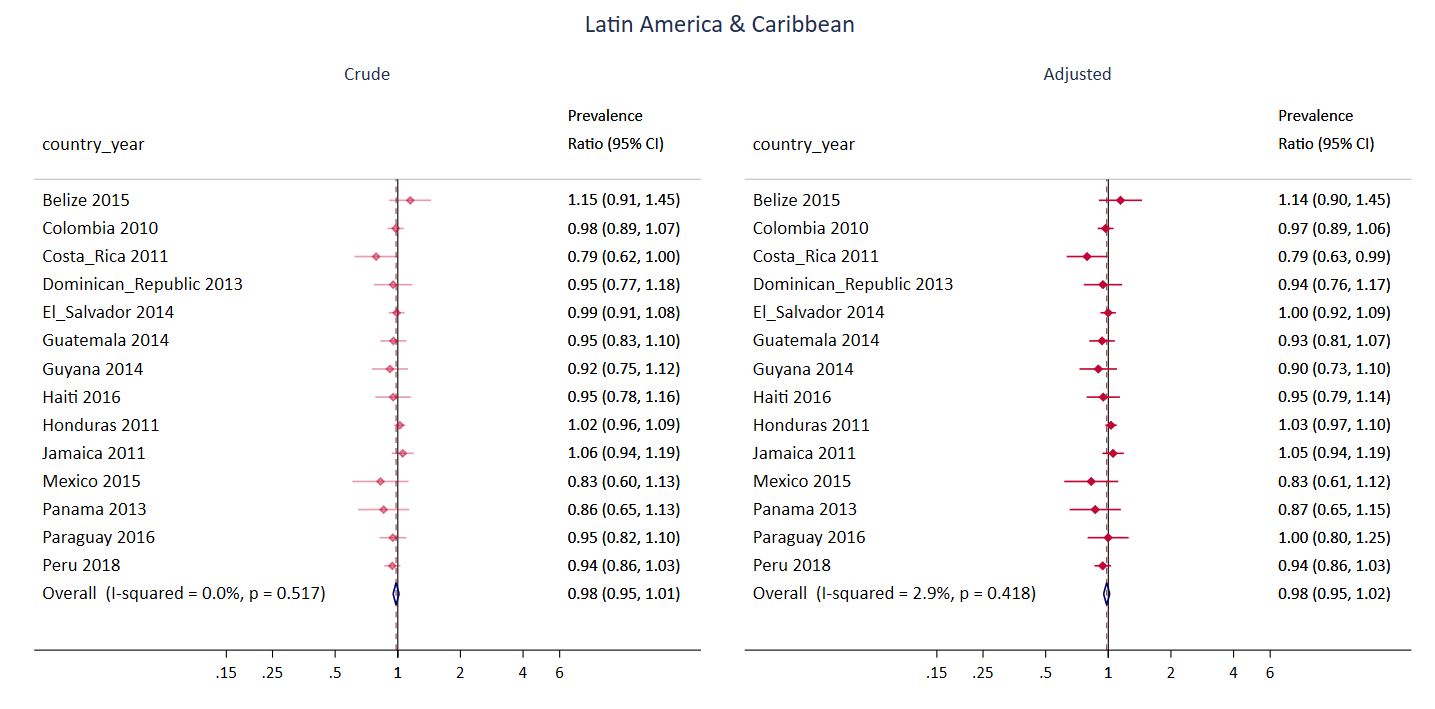


**Figure A8. Crude and adjusted prevalence ratio for full immunization in FHH (any male) in Latin America & Caribbean**


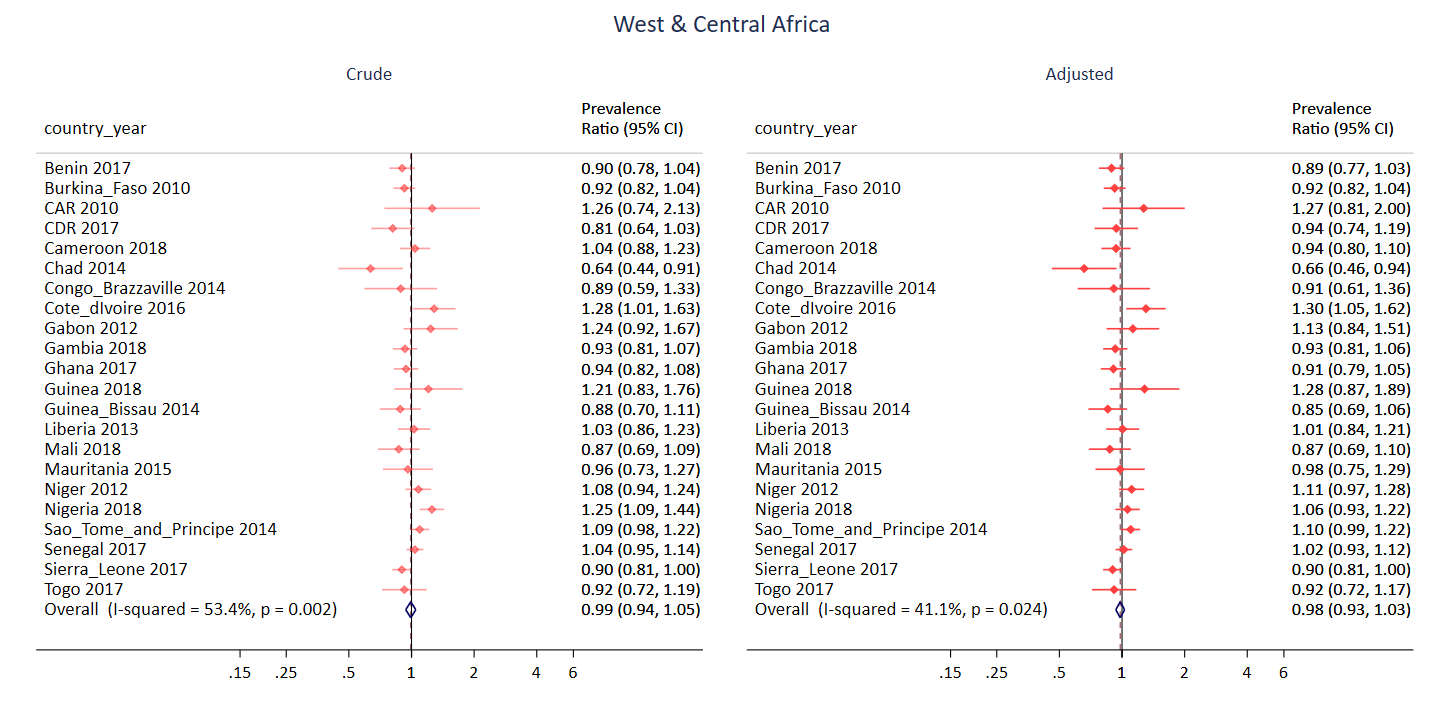


**Figure A9. Crude and adjusted prevalence ratio for full immunization in FHH (no male) in West & Central Africa**


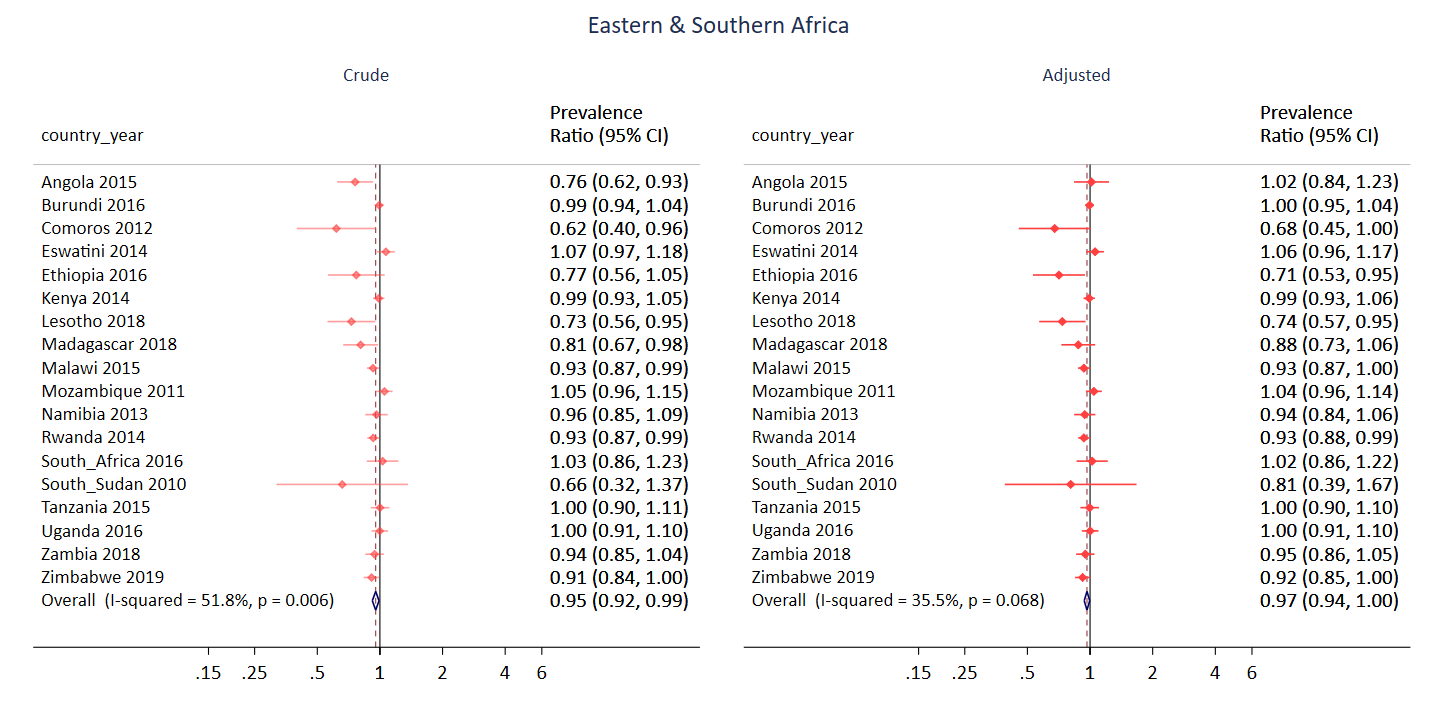


**Figure A10. Crude and adjusted prevalence ratio for full immunization in FHH (no male) in Easter & Southern Africa**


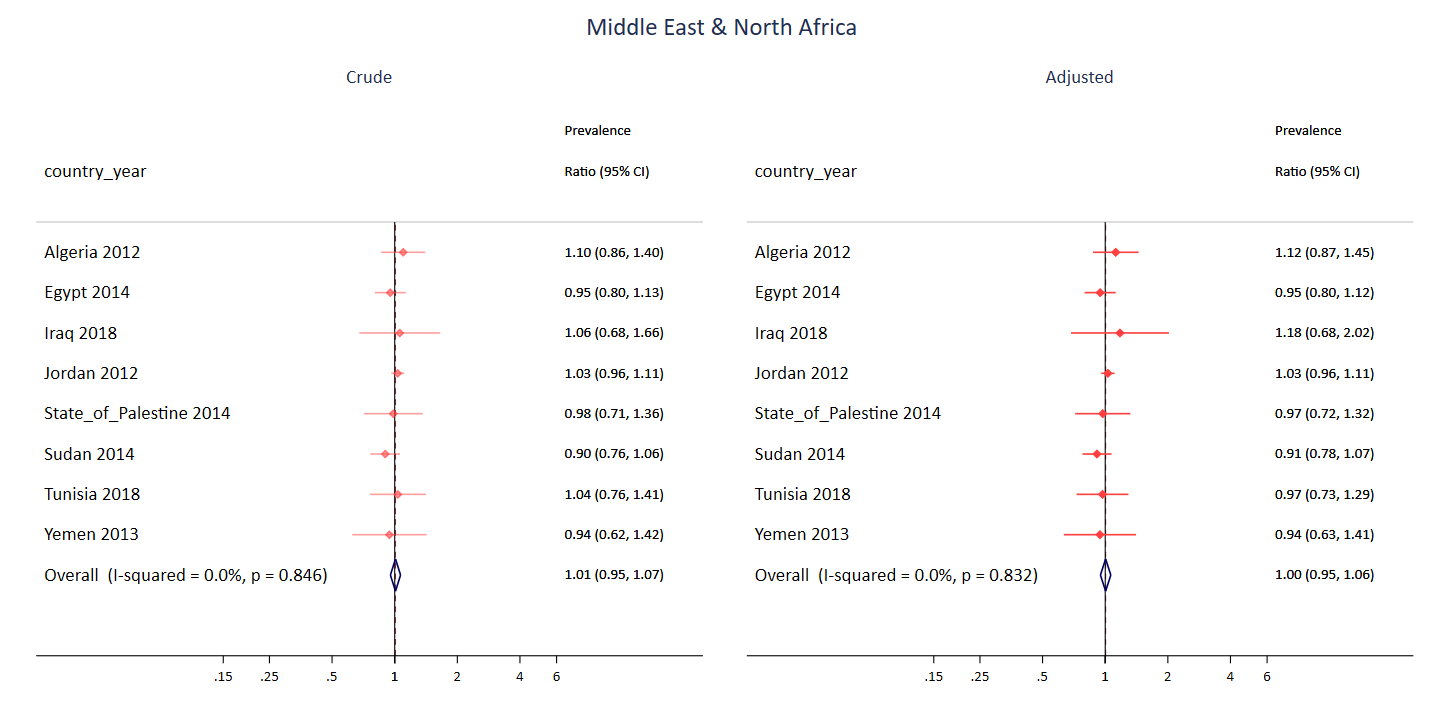


**Figure A11. Crude and adjusted prevalence ratio for full immunization in FHH (no male) in Middle East & North Africa**


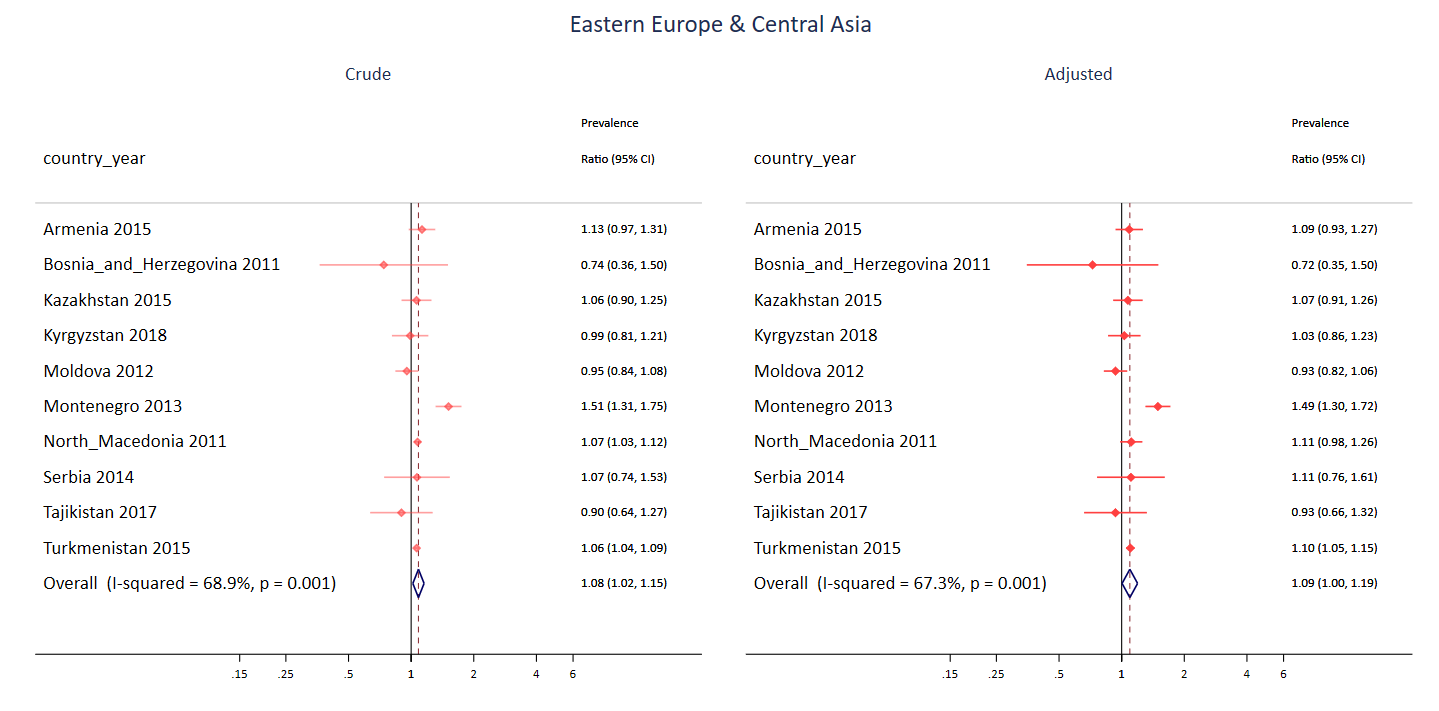


**Figure A12. Crude and adjusted prevalence ratio for full immunization in FHH (no male) in Eastern Europe & Central Asia**


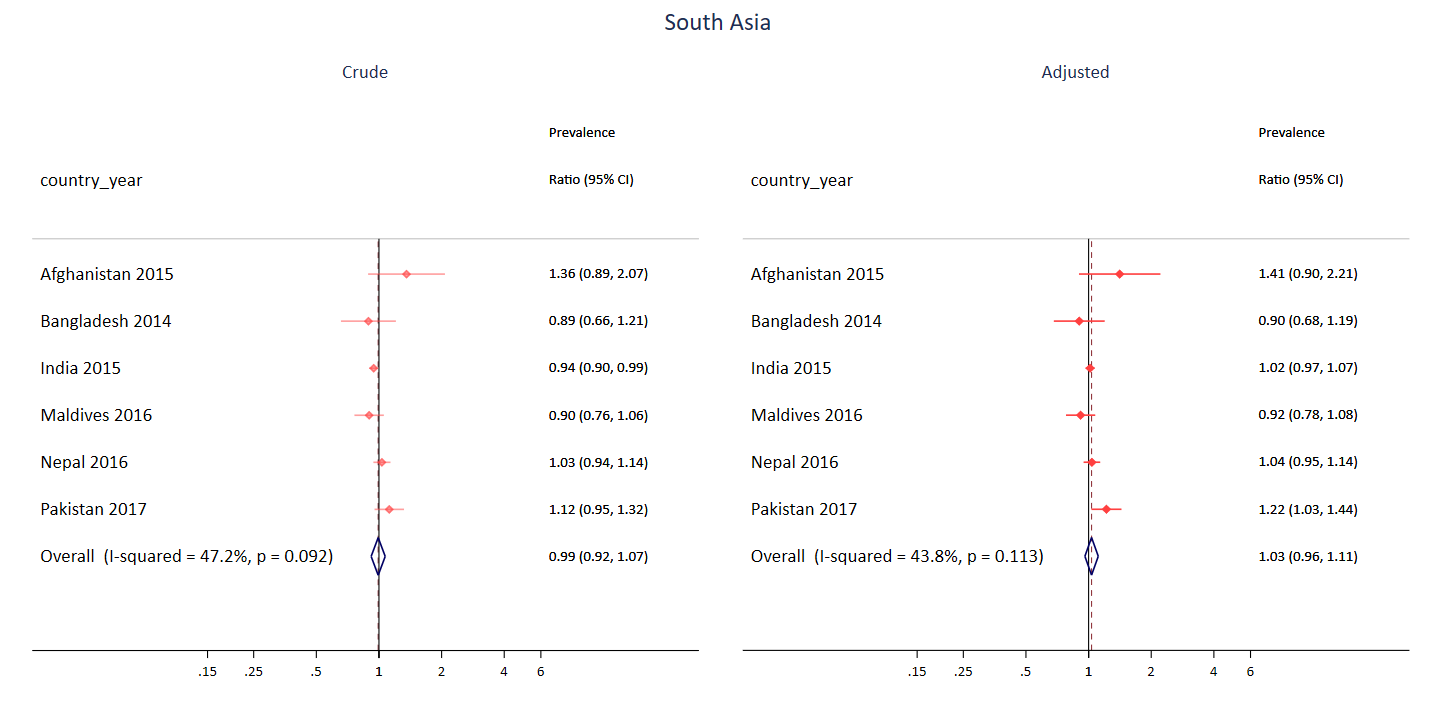


**Figure A13. Crude and adjusted prevalence ratio for full immunization in FHH (no male) in South Asia**


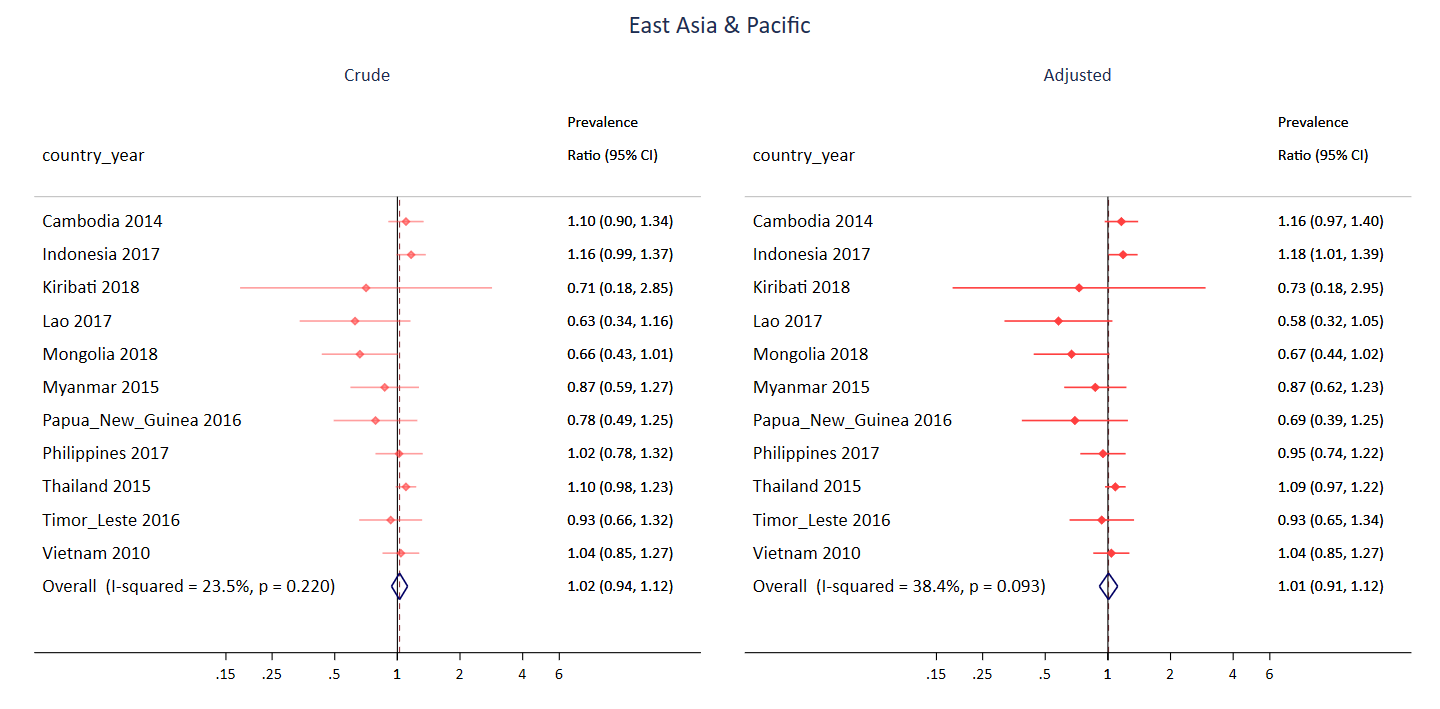


**Figure A14. Crude and adjusted prevalence ratio for full immunization in FHH (no male) in East Asia & Pacific**


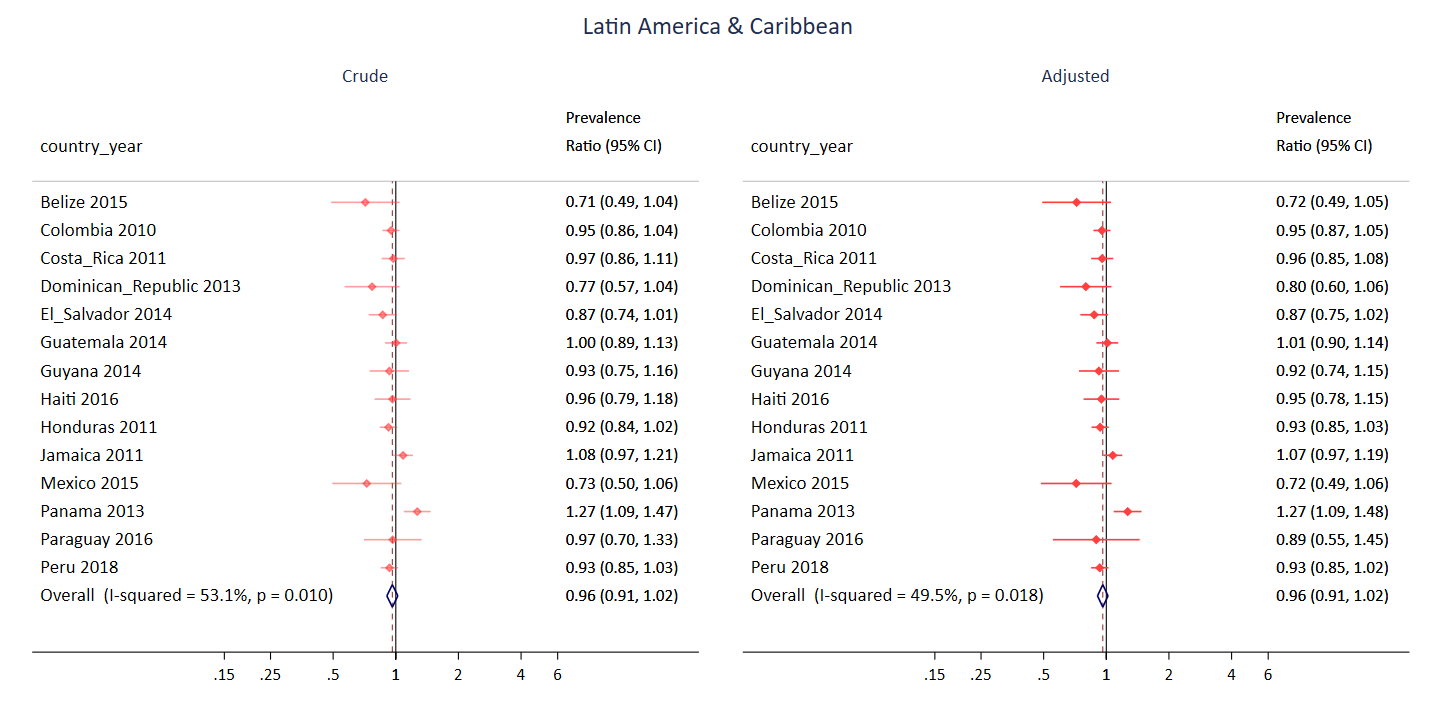


**Figure A15. Crude and adjusted prevalence ratio for full immunization in FHH (no male) in Latin America & Caribbean**


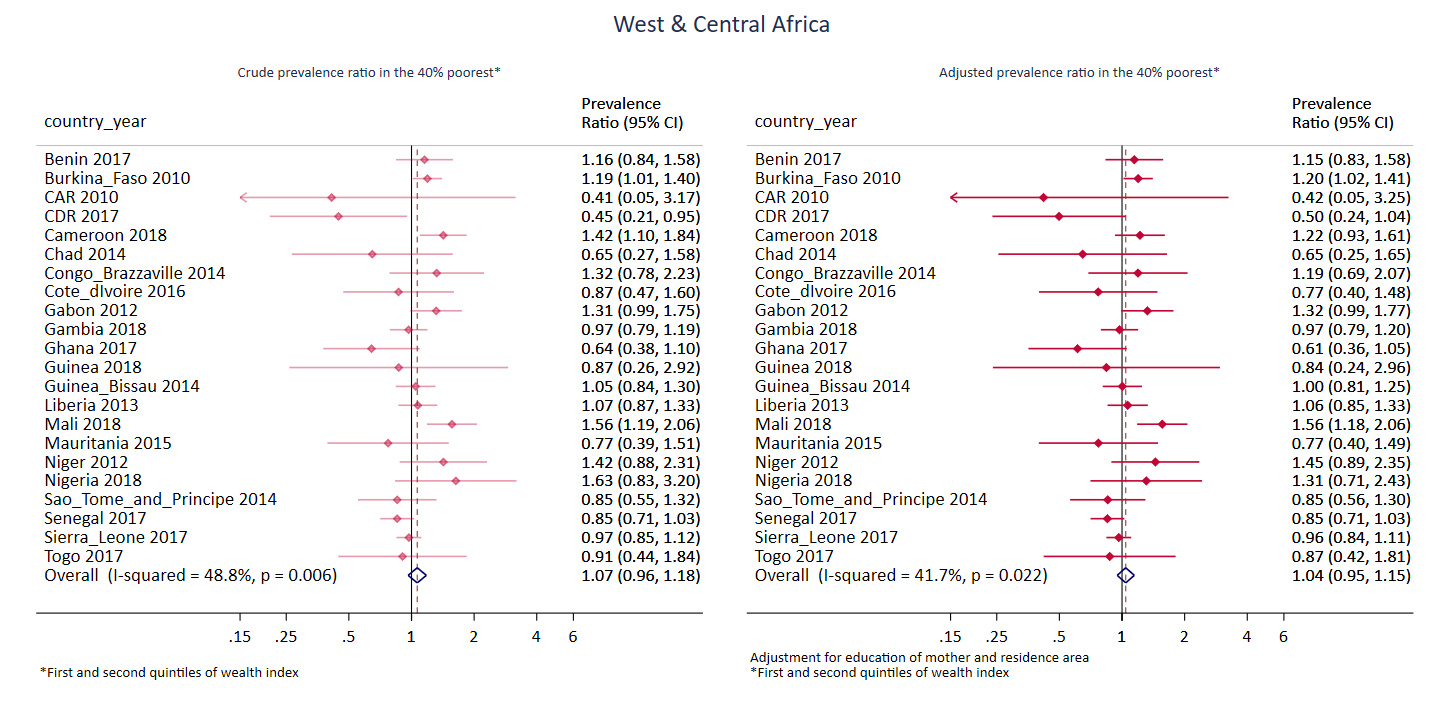


**Figure A16. Crude and adjusted prevalence ratio for full immunization in FHH (any male) in West & Central Africa. Analysis restricted to 40% poorest in each country.**


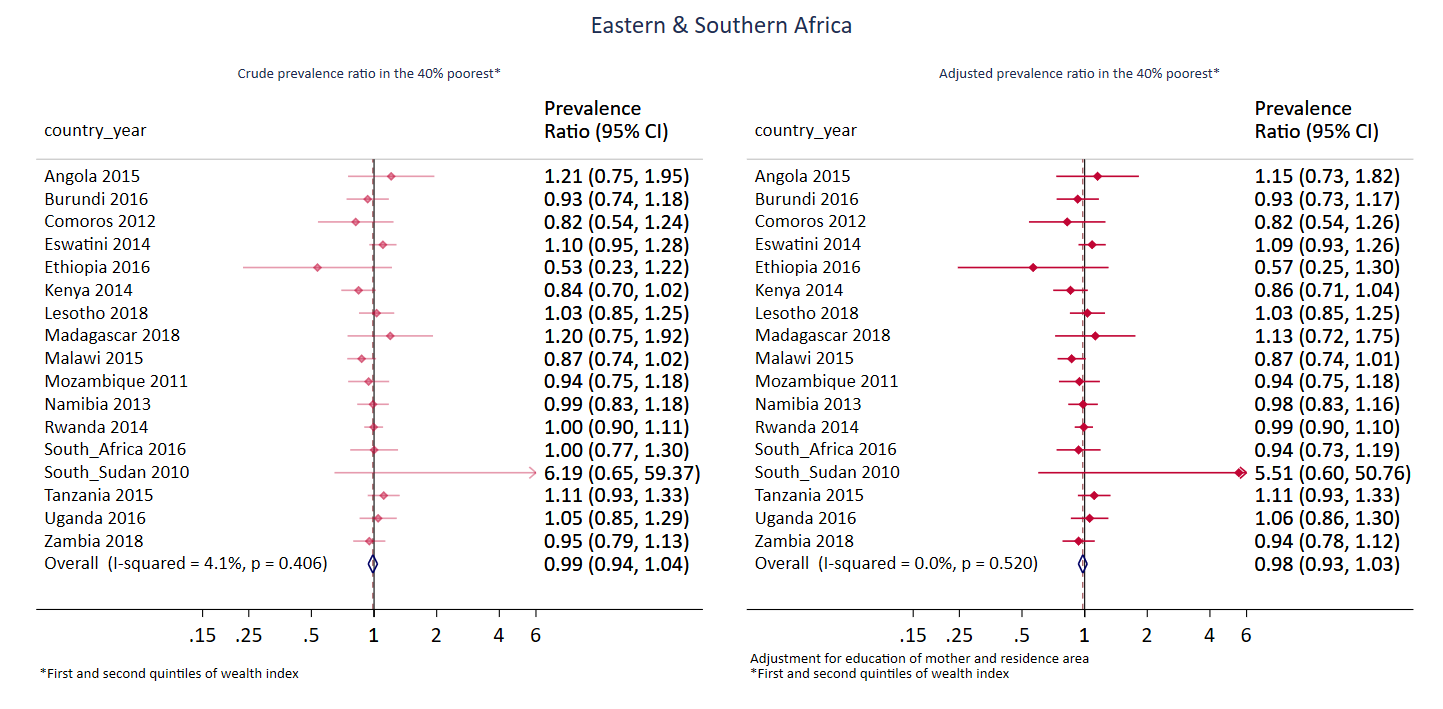


**Figure A17. Crude and adjusted prevalence ratio for full immunization in FHH (any male) in Easter & Southern Africa. Analysis restricted to 40% poorest in each country.**


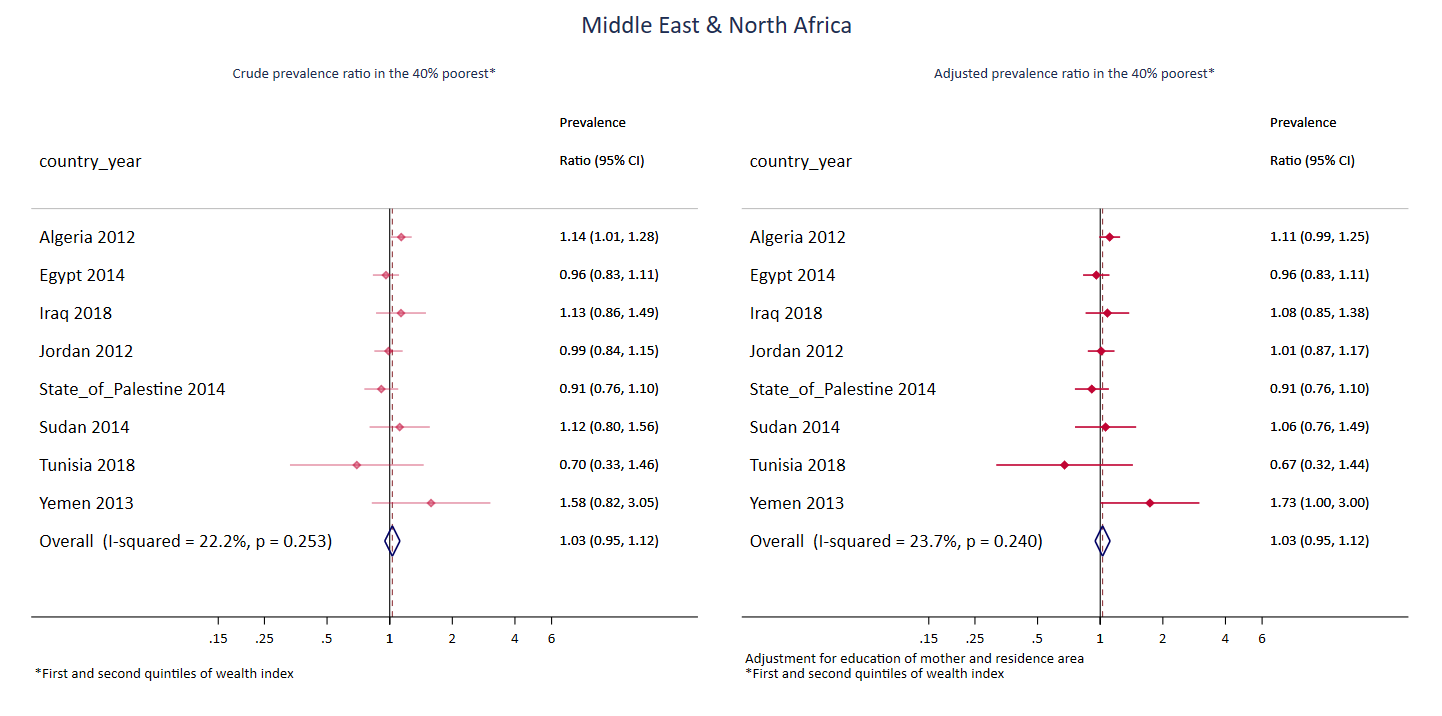


**Figure A18. Crude and adjusted prevalence ratio for full immunization in FHH (any male) in Middle East & North Africa. Analysis restricted to 40% poorest in each country.**


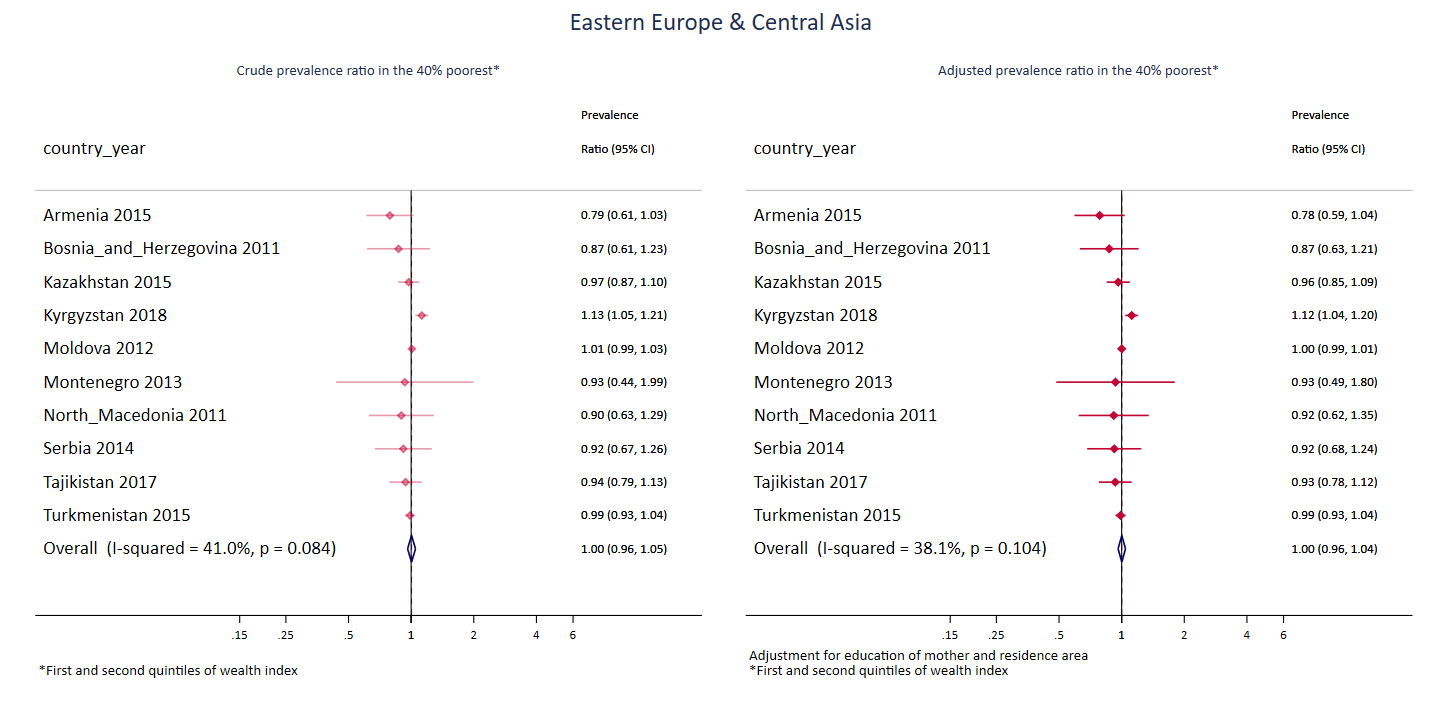


**Figure A19. Crude and adjusted prevalence ratio for full immunization in FHH (any male) in Eastern Europe & Central Asia. Analysis restricted to 40% poorest in each country.**


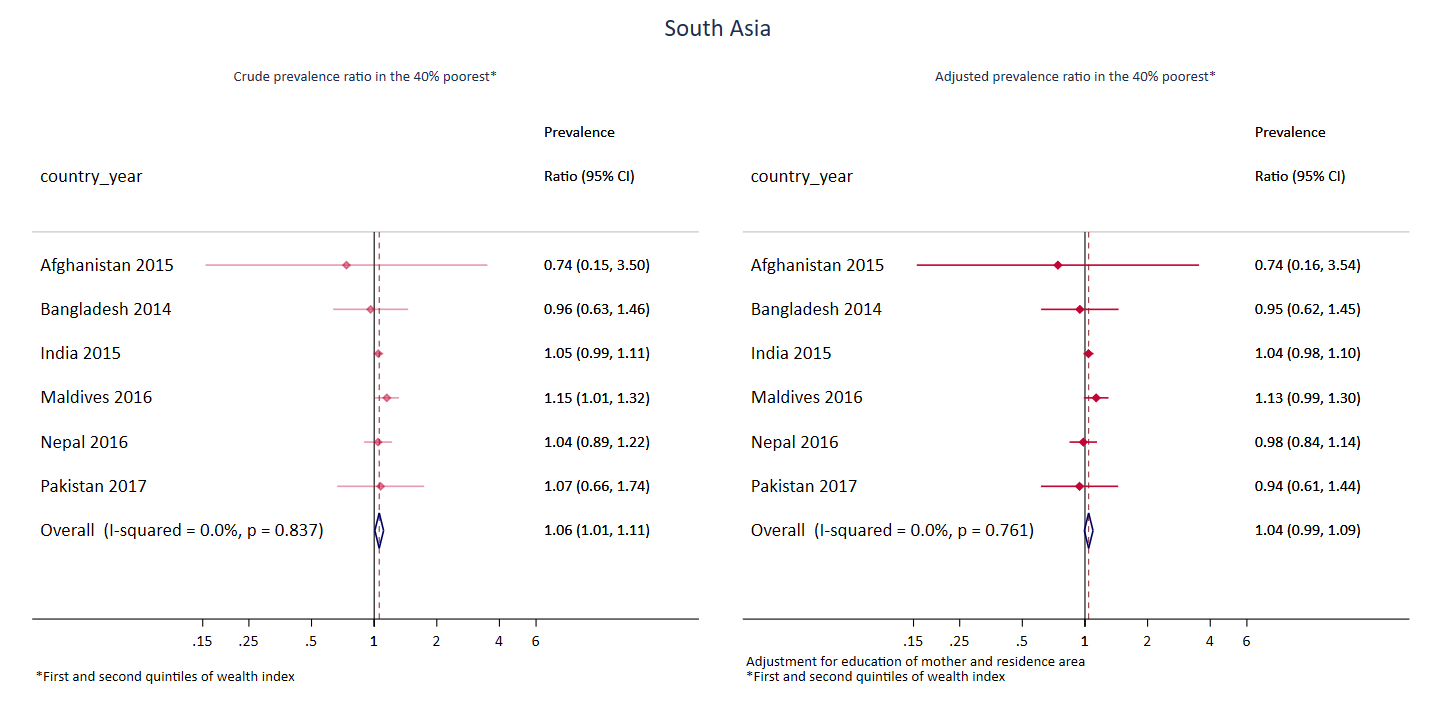


**Figure A20. Crude and adjusted prevalence ratio for full immunization in FHH (any male) in South Asia. Analysis restricted to 40% poorest in each country.**


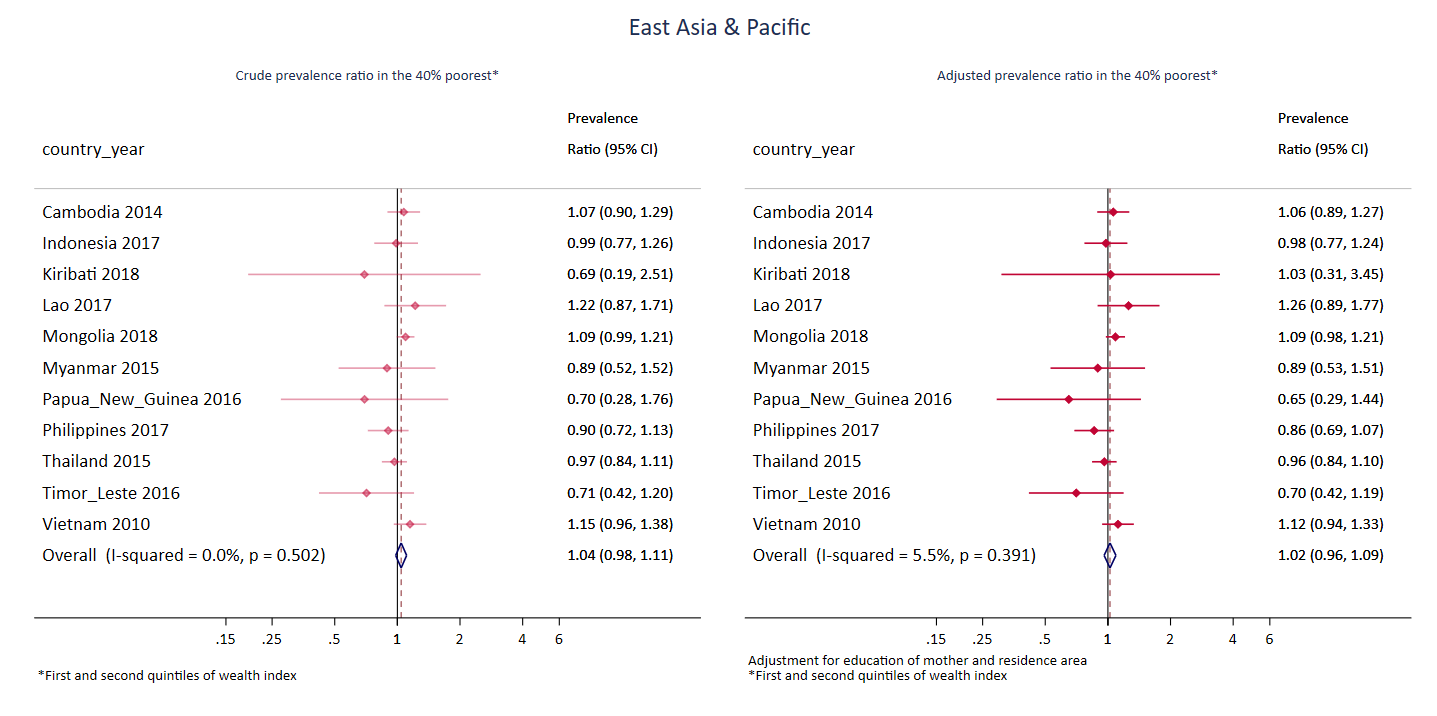


**Figure A21. Crude and adjusted prevalence ratio for full immunization in FHH (any male) in East Asia & Pacific. Analysis restricted to 40% poorest in each country.**


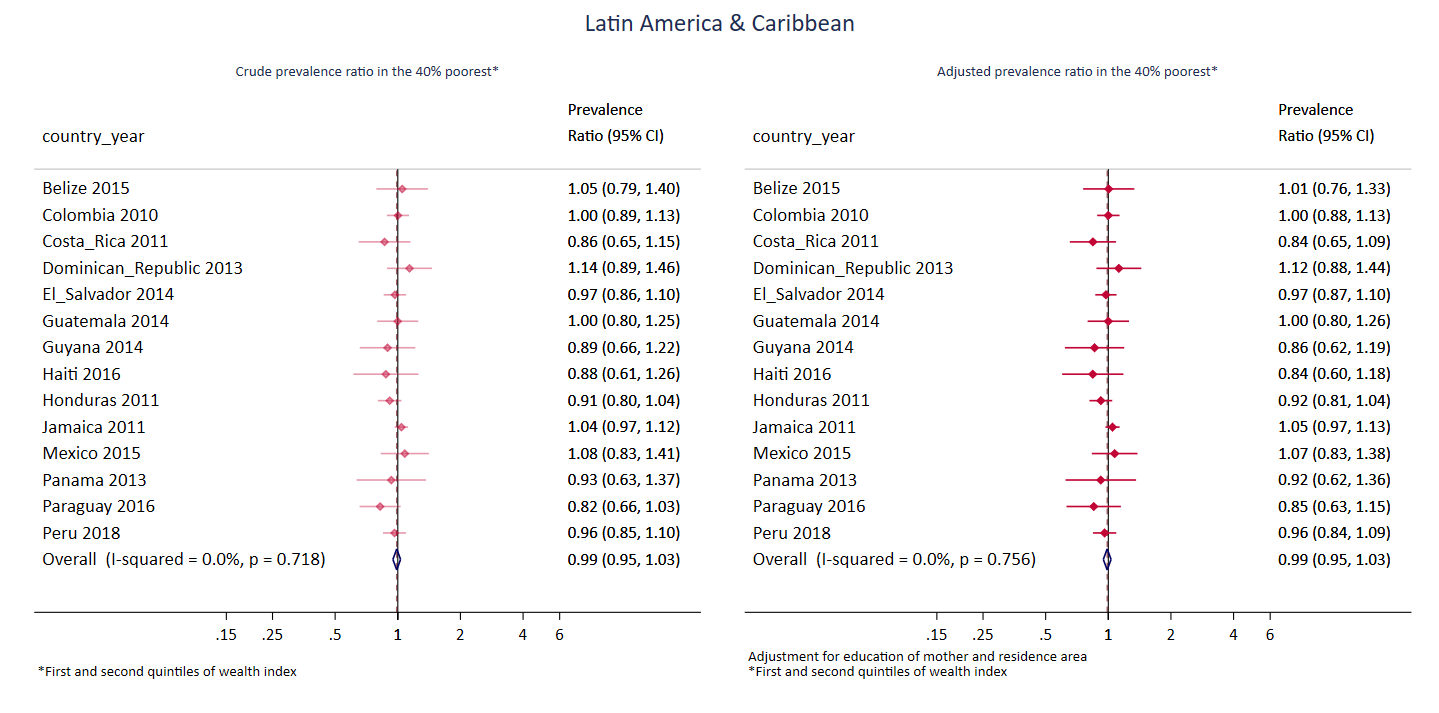


**Figure A22. Crude and adjusted prevalence ratio for full immunization in FHH (any male) in Latin America & Caribbean. Analysis restricted to 40% poorest in each country.**


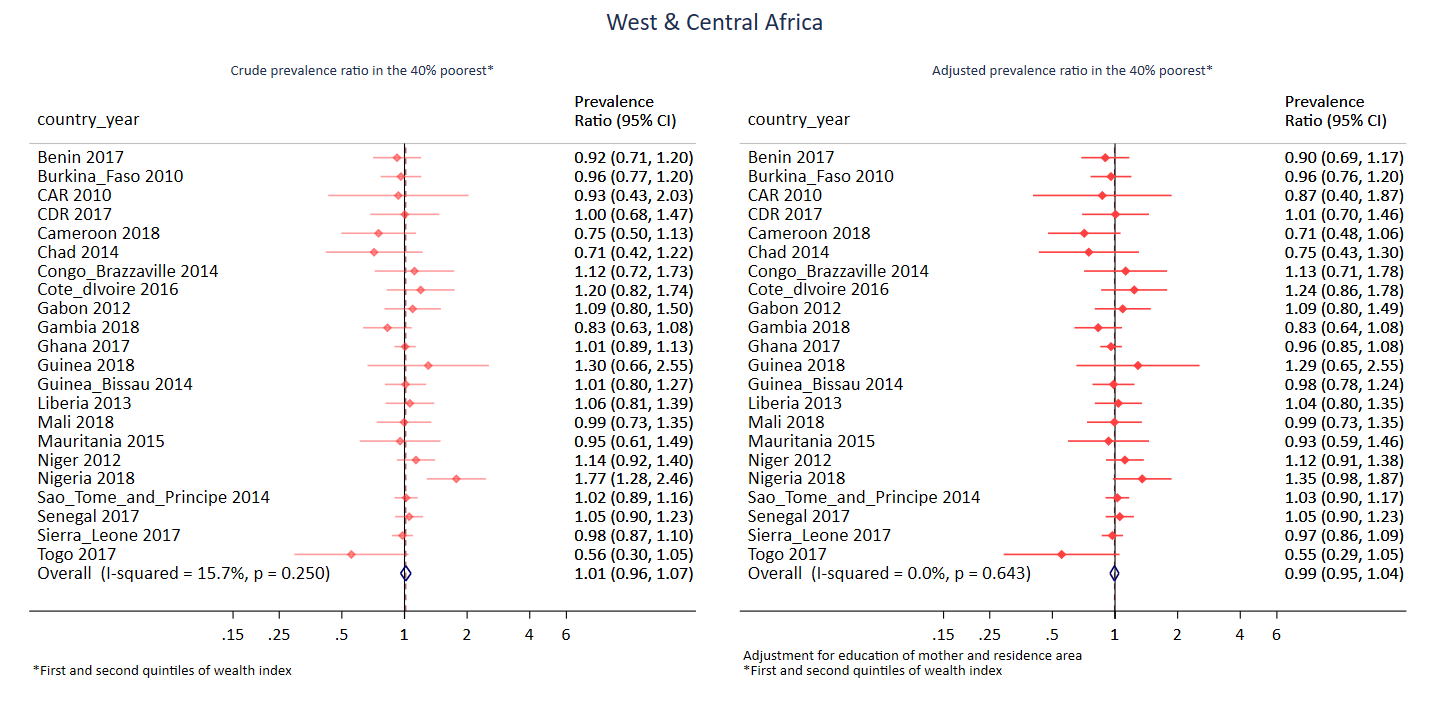


**Figure A23. Crude and adjusted prevalence ratio for full immunization in FHH (no male) in West & Central Africa. Analysis restricted to 40% poorest in each country.**


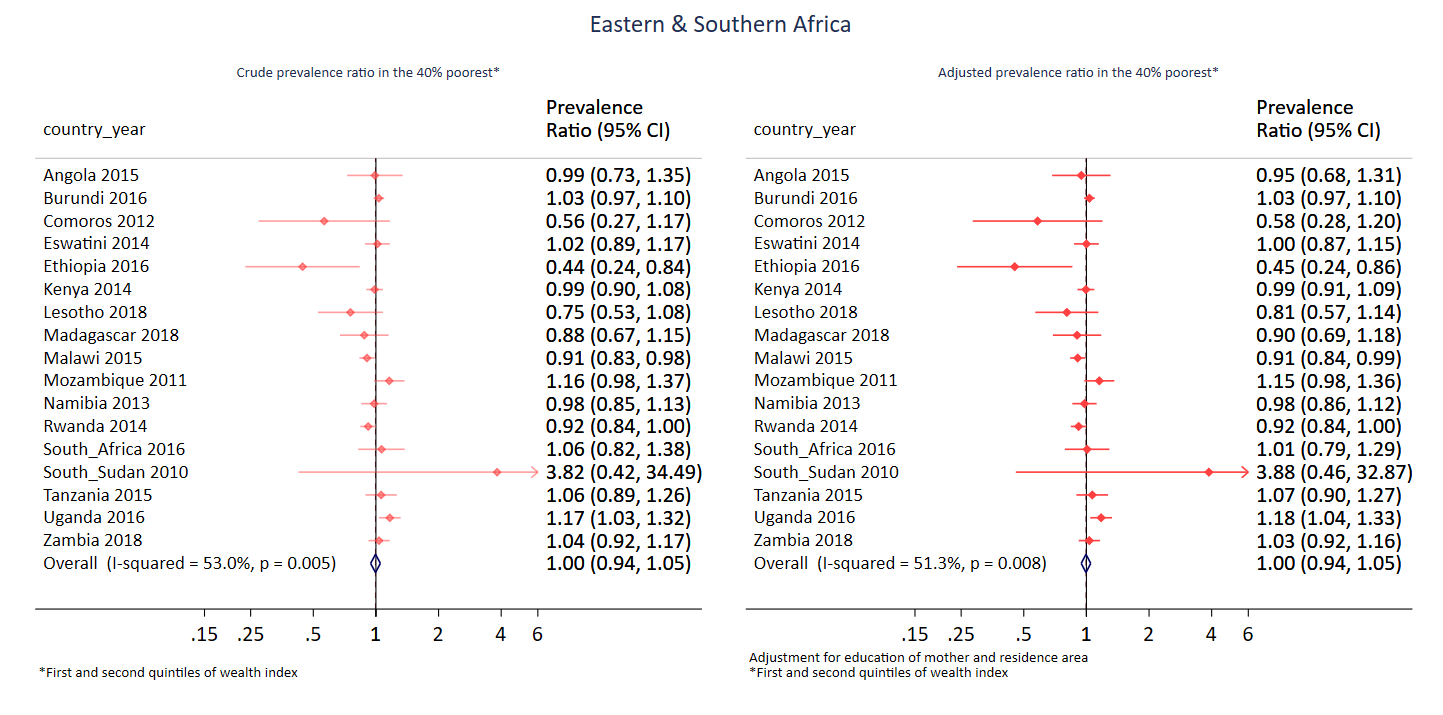


**Figure A24. Crude and adjusted prevalence ratio for full immunization in FHH (no male) in Easter & Southern Africa. Analysis restricted to 40% poorest in each country.**


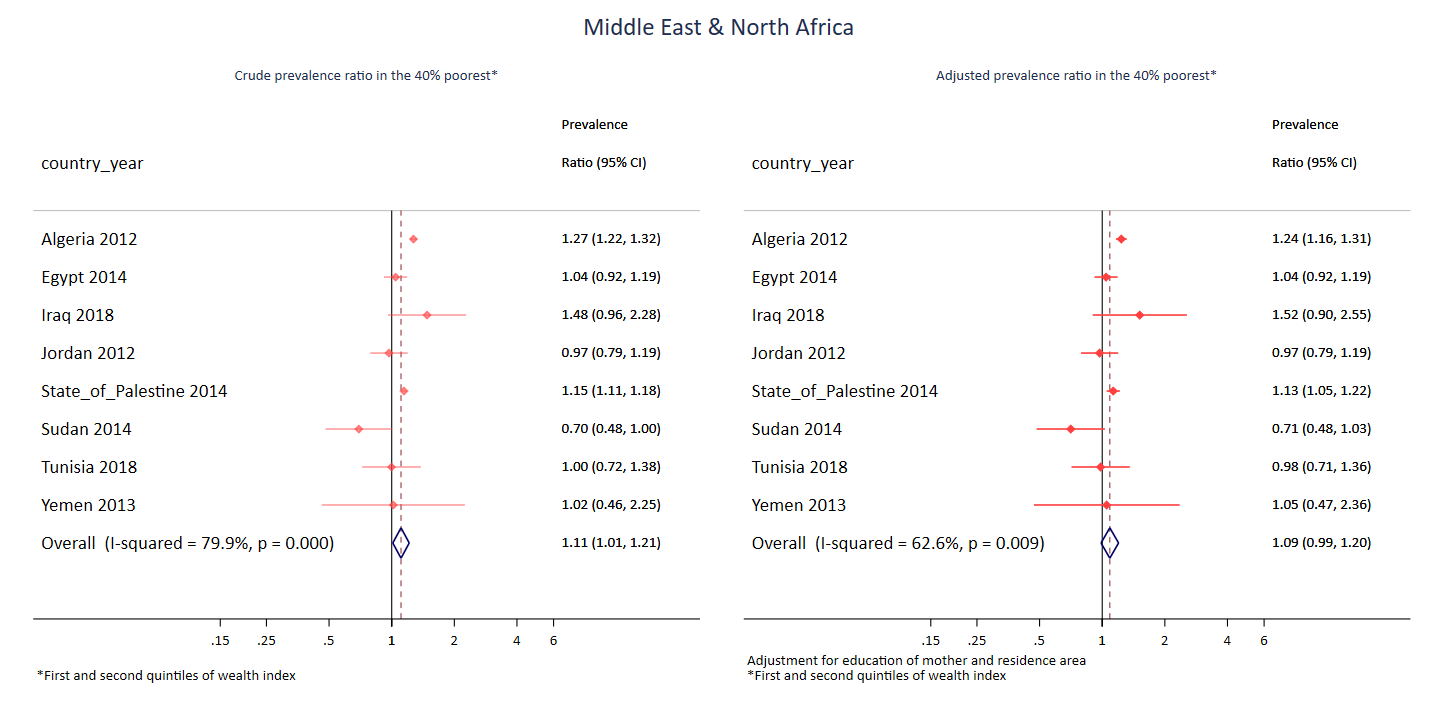


**Figure A25. Crude and adjusted prevalence ratio for full immunization in FHH (no male) in Middle East & North Africa. Analysis restricted to 40% poorest in each country.**


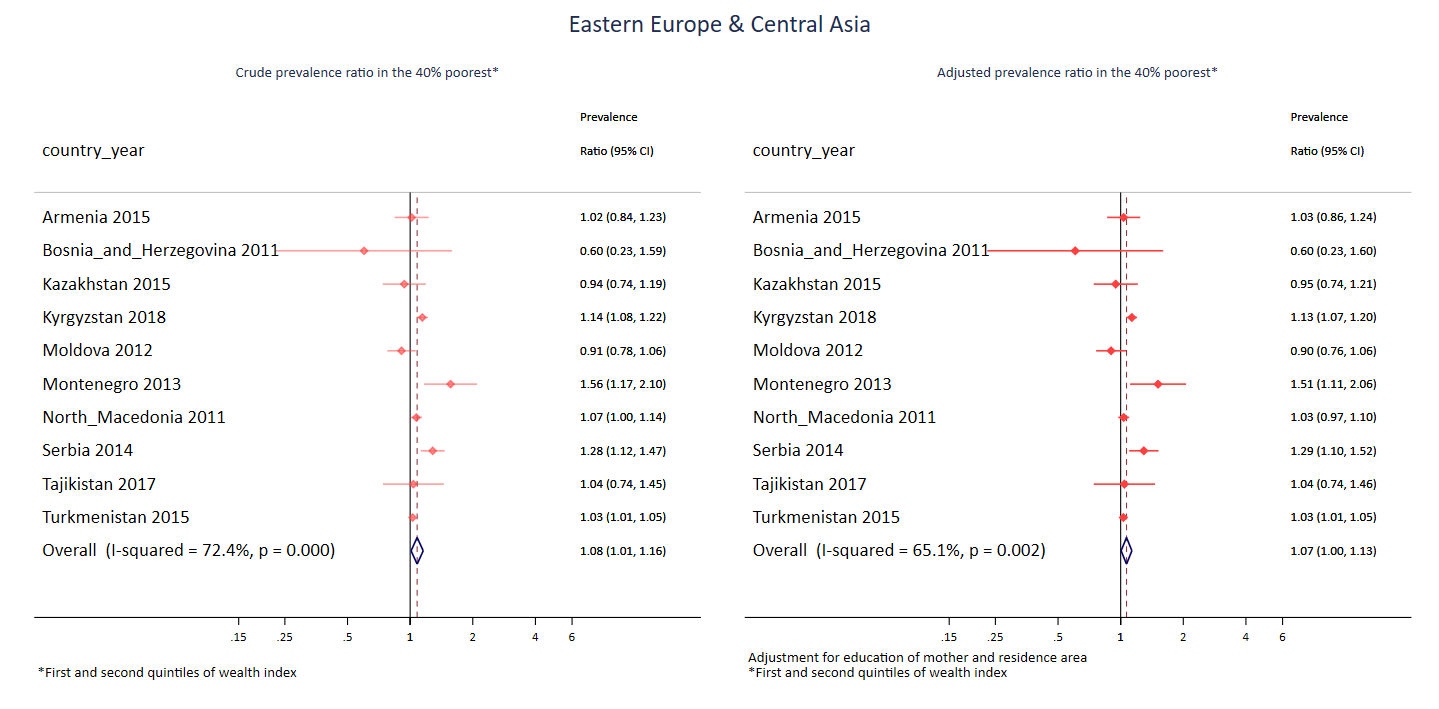


**Figure A26. Crude and adjusted prevalence ratio for full immunization in FHH (no male) in Eastern Europe & Central Asia. Analysis restricted to 40% poorest in each country.**


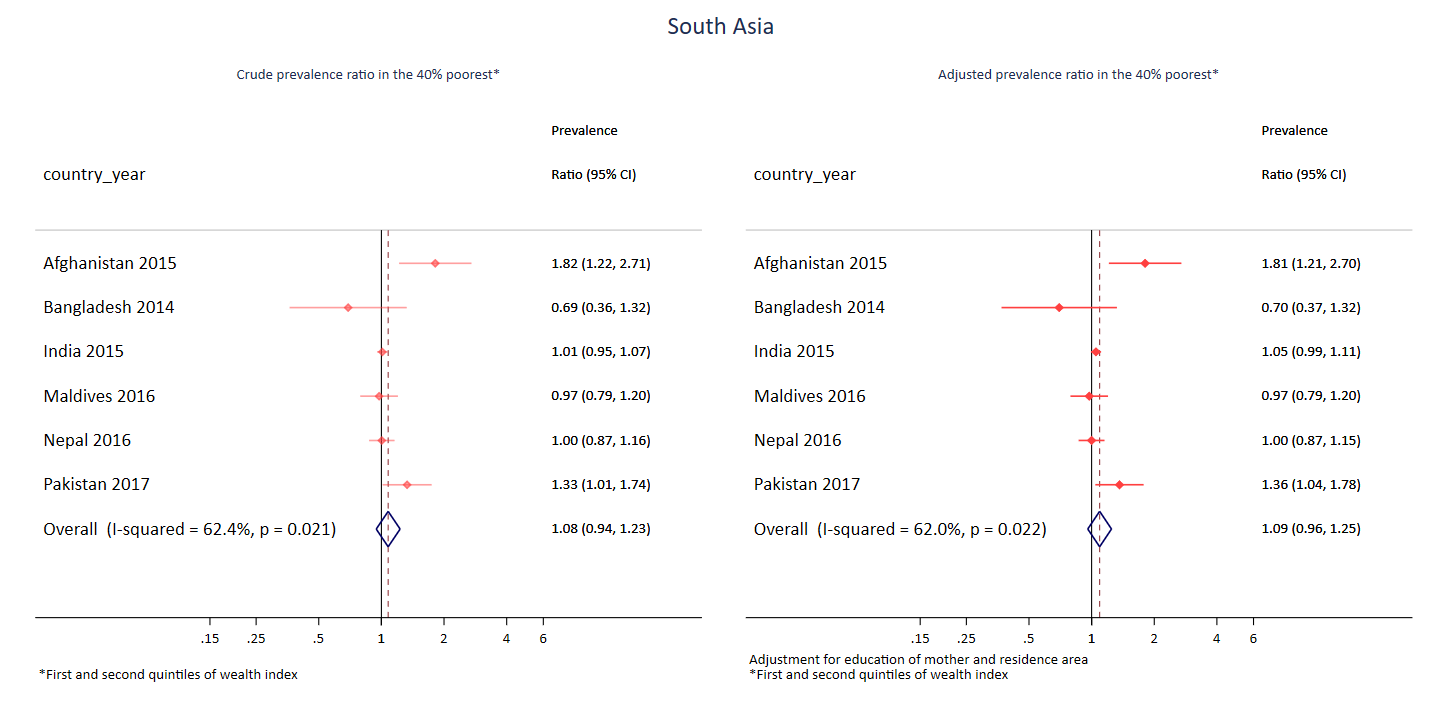


**Figure A27. Crude and adjusted prevalence ratio for full immunization in FHH (no male) in South Asia. Analysis restricted to 40% poorest in each country.**


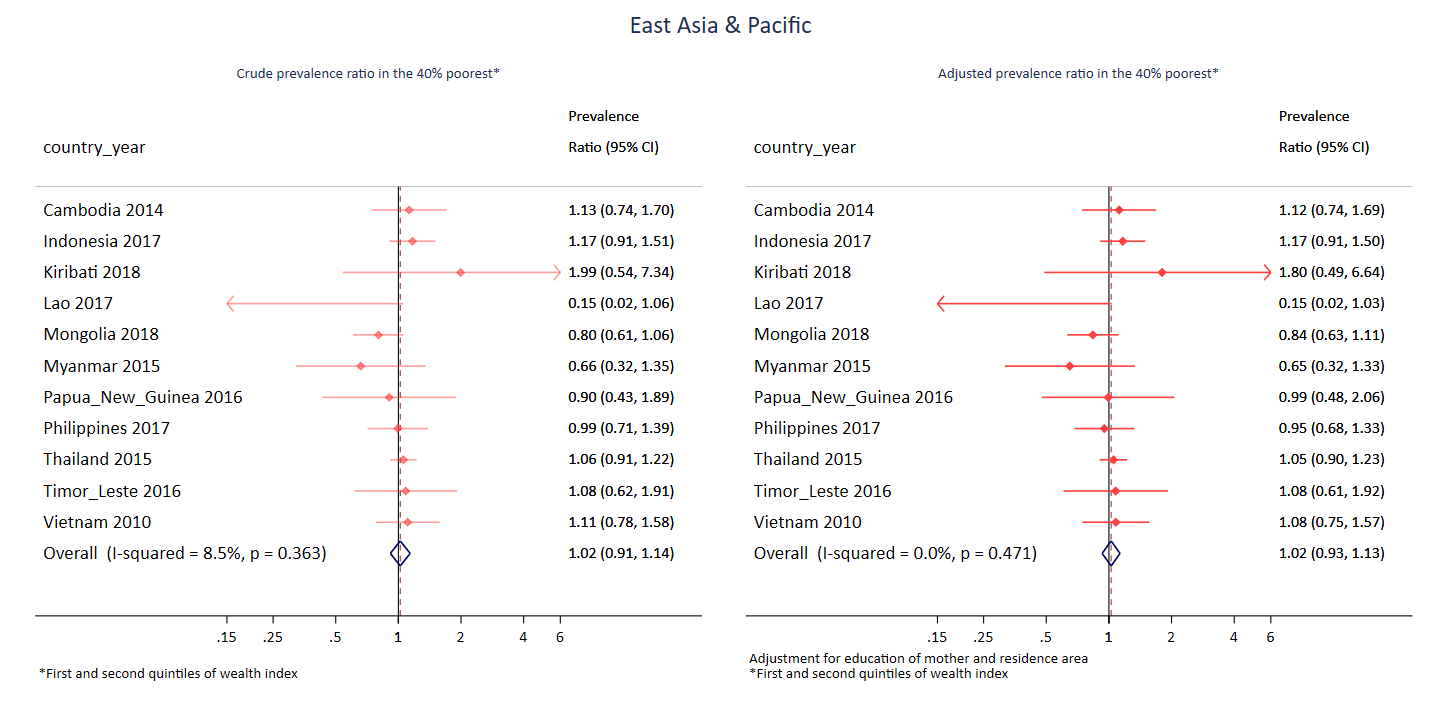


**Figure A28. Crude and adjusted prevalence ratio for full immunization in FHH (no male) in East Asia & Pacific. Analysis restricted to 40% poorest in each country.**


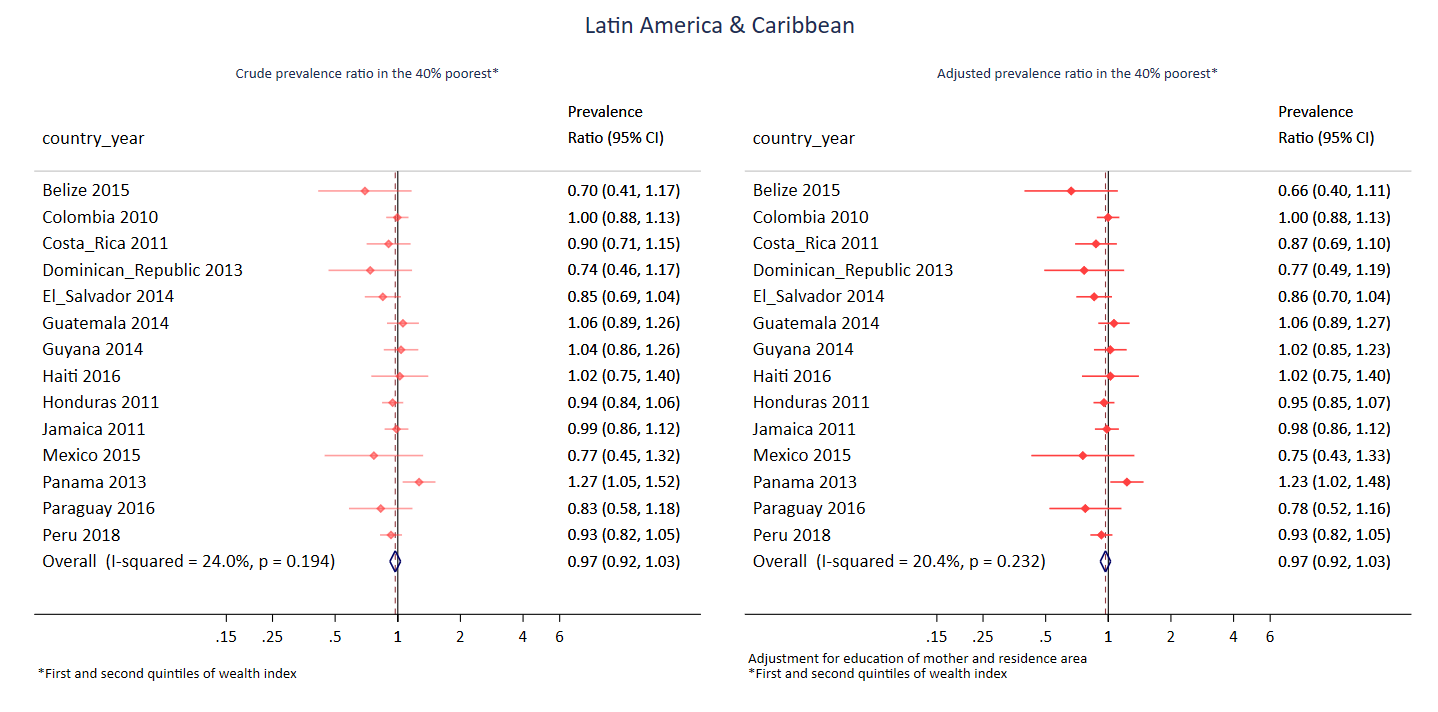


**Figure A29. Crude and adjusted prevalence ratio for full immunization in FHH (no male) in Latin America & Caribbean. Analysis restricted to 40% poorest in each country.**

#
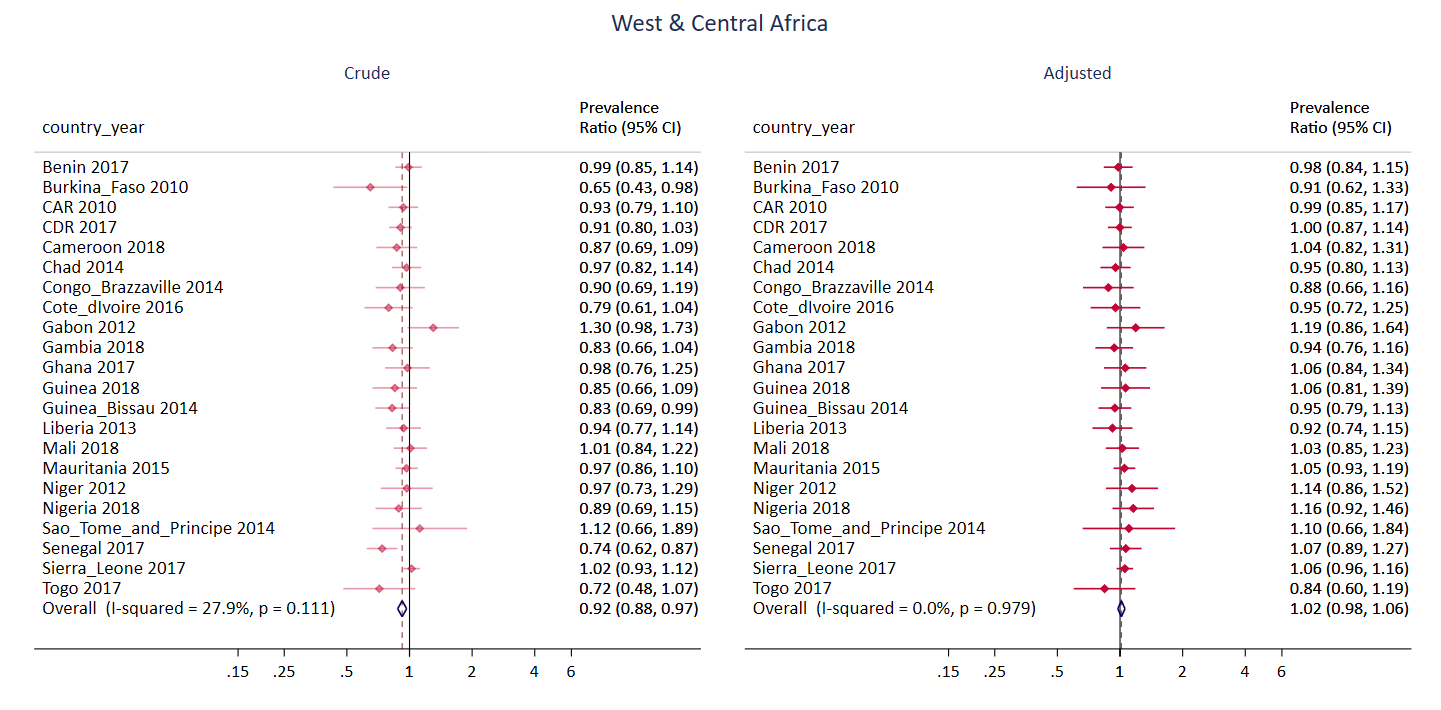


**Figure A30. Crude and adjusted prevalence ratio for stunting in FHH (any male) in West & Central Africa**


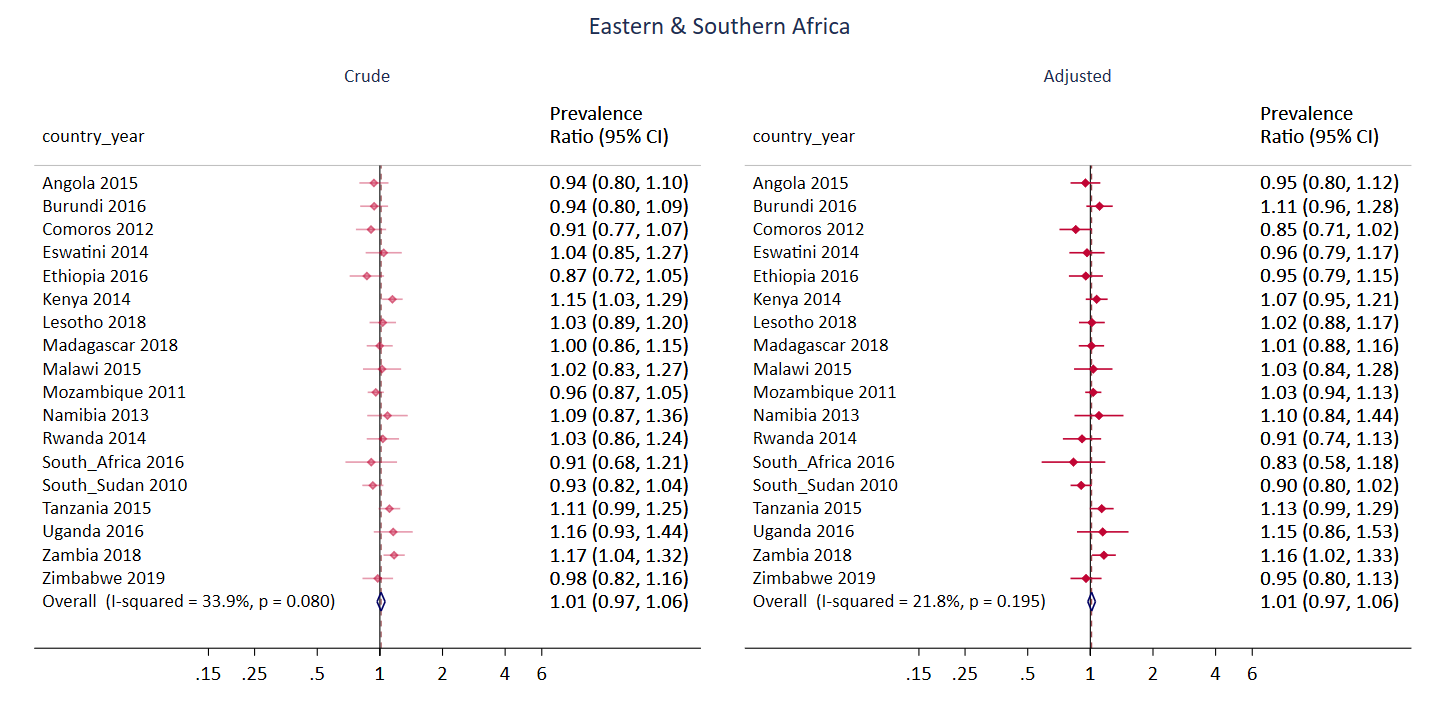


**Figure A31. Crude and adjusted prevalence ratio for stunting in FHH (any male) in Easter & Southern Africa**


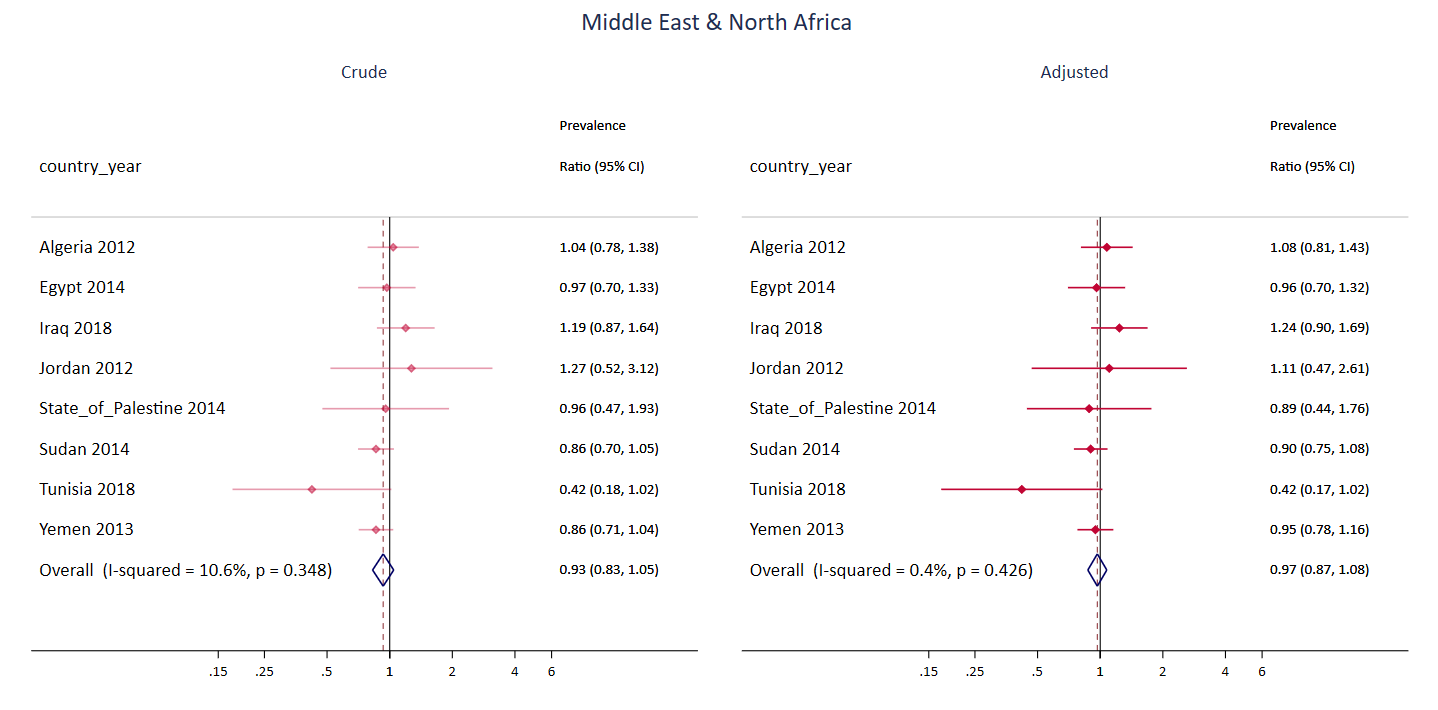


**Figure A32. Crude and adjusted prevalence ratio for stunting in FHH (any male) in Middle East & North Africa**


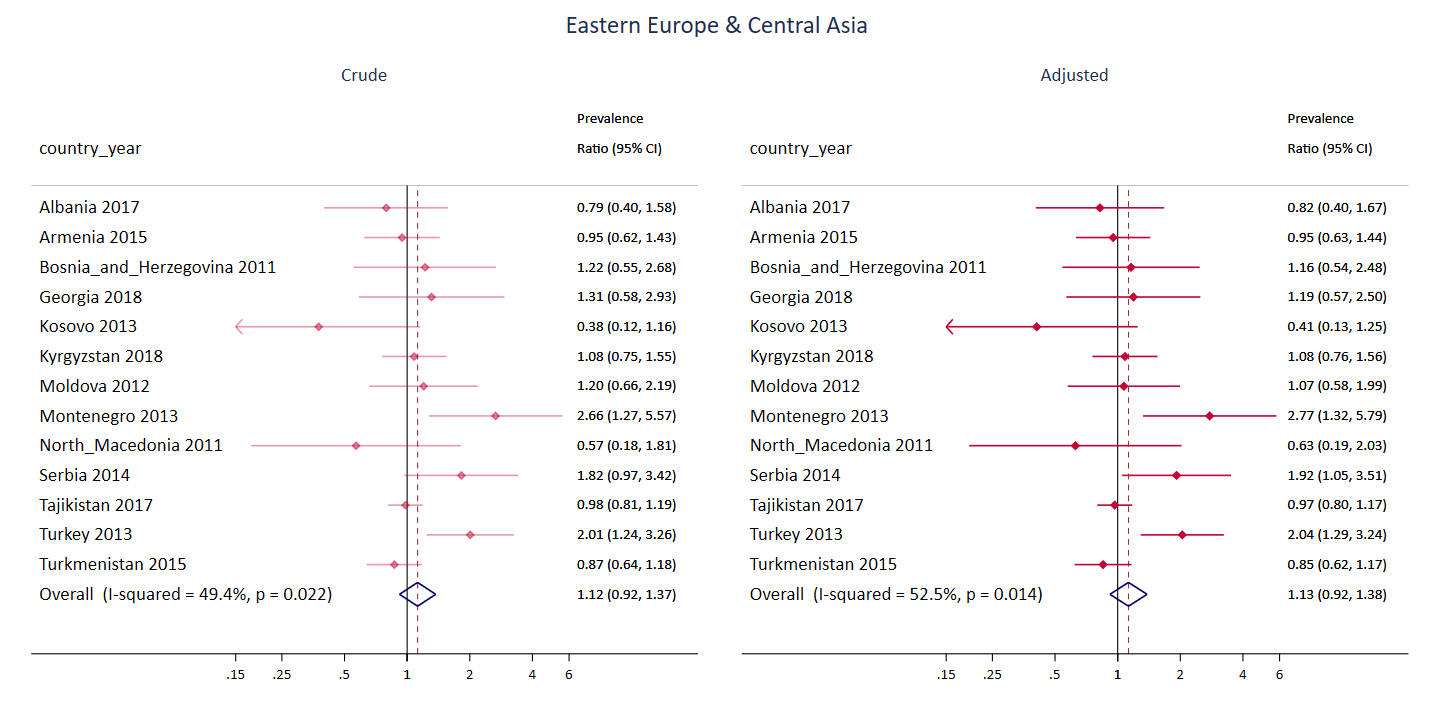


**Figure A33. Crude and adjusted prevalence ratio for stunting in FHH (any male) in Eastern Europe & Central Asia**


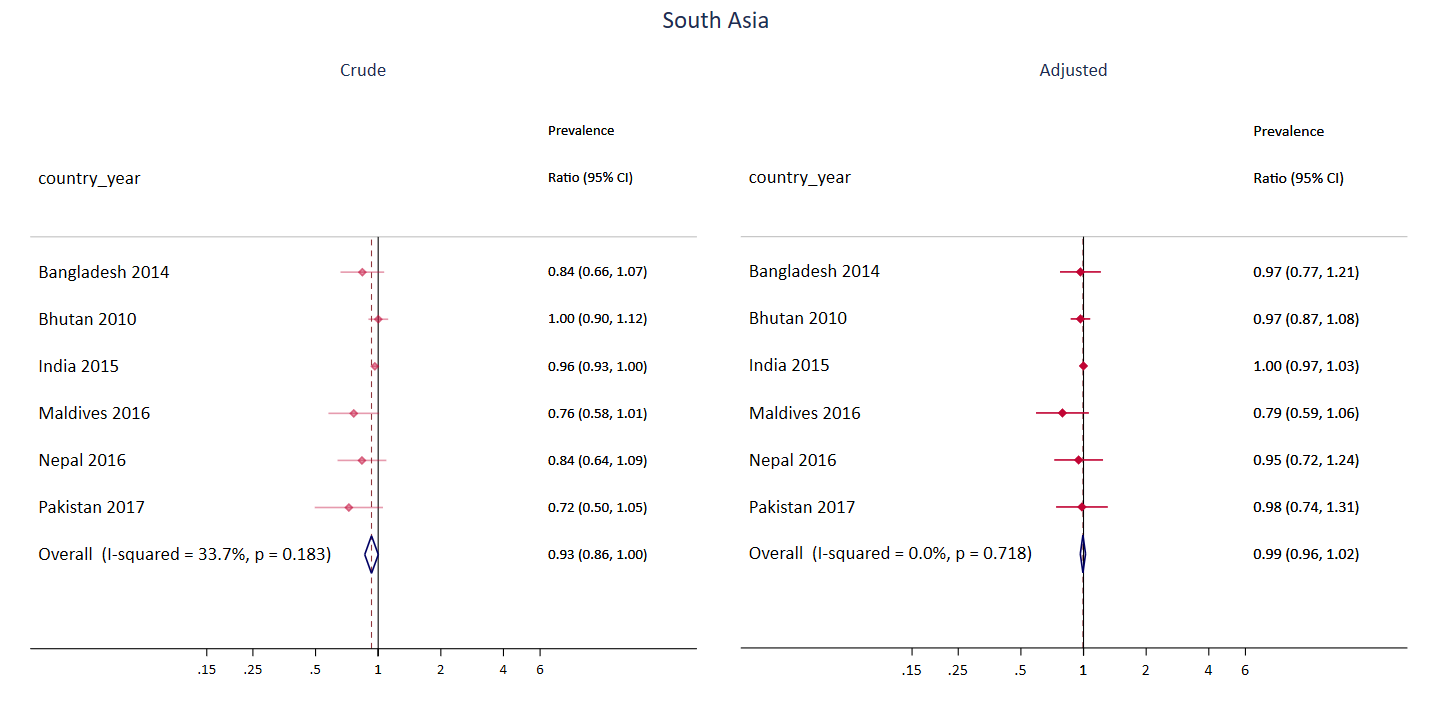


**Figure A34. Crude and adjusted prevalence ratio for stunting in FHH (any male) in South Asia**


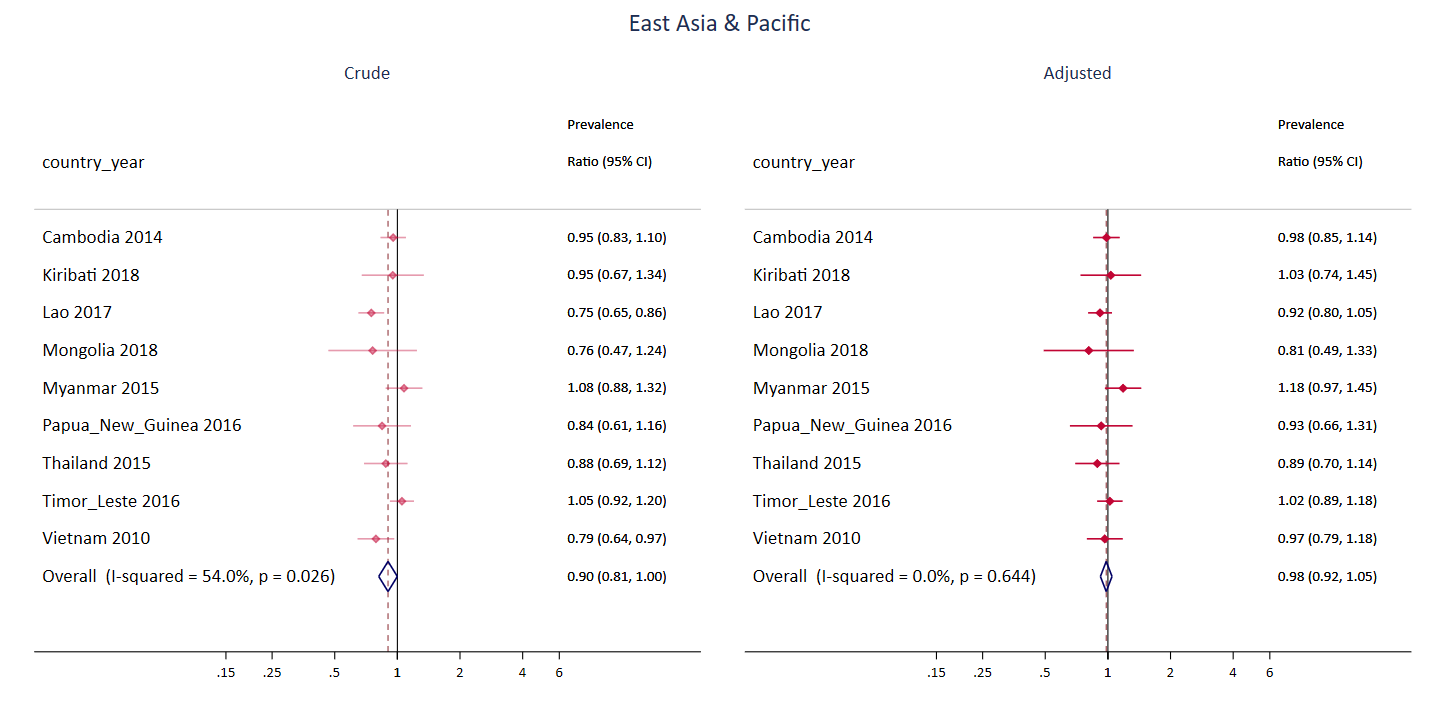


**Figure A35. Crude and adjusted prevalence ratio for stunting in FHH (any male) in East Asia & Pacific**


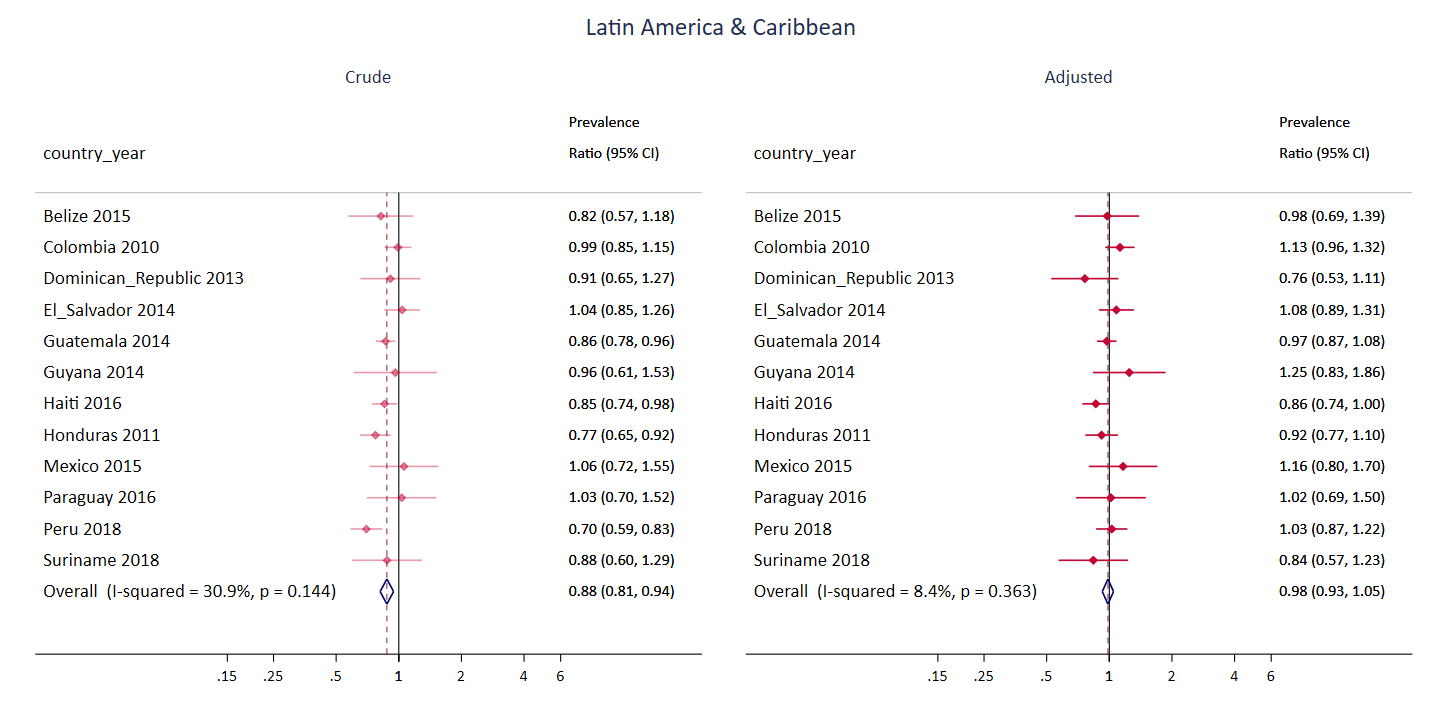


**Figure A36. Crude and adjusted prevalence ratio for stunting in FHH (any male) in Latin America & Caribbean**


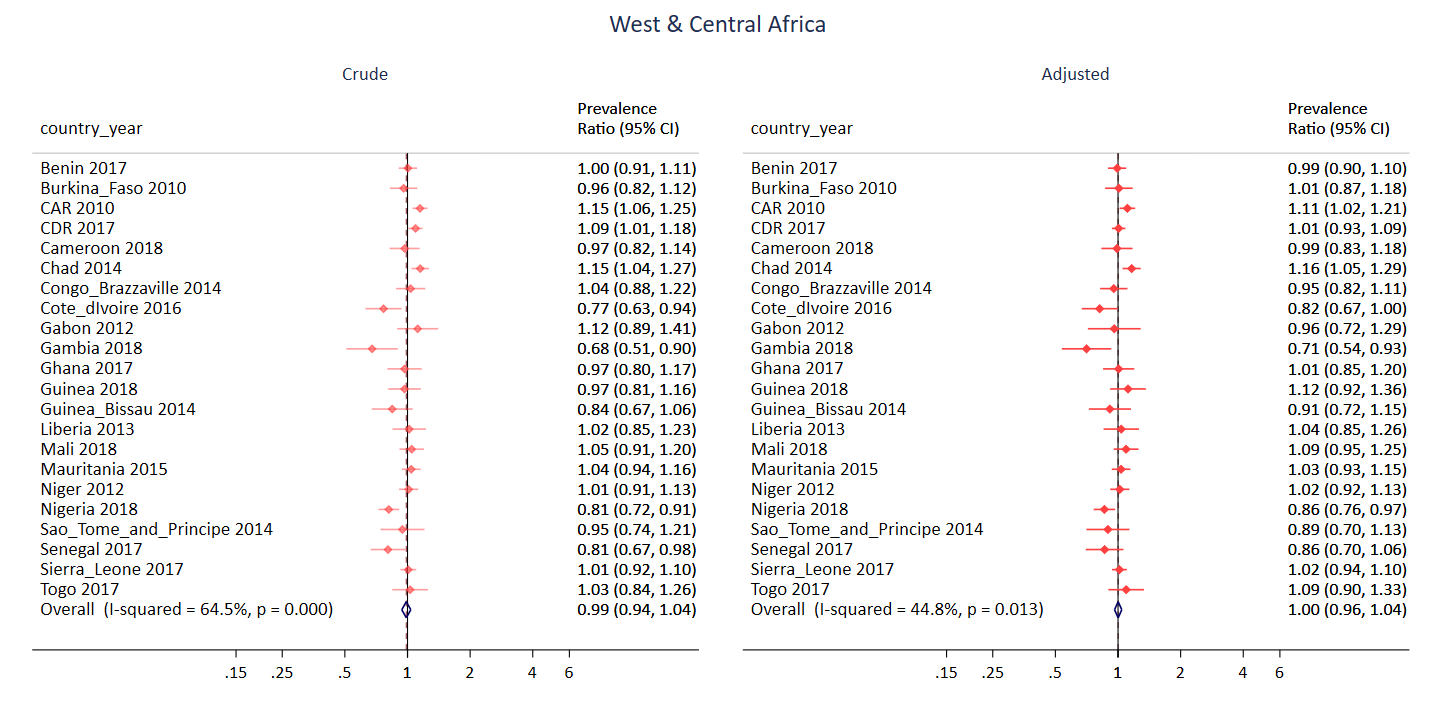


**Figure A37. Crude and adjusted prevalence ratio for stunting in FHH (no male) in West & Central Africa**


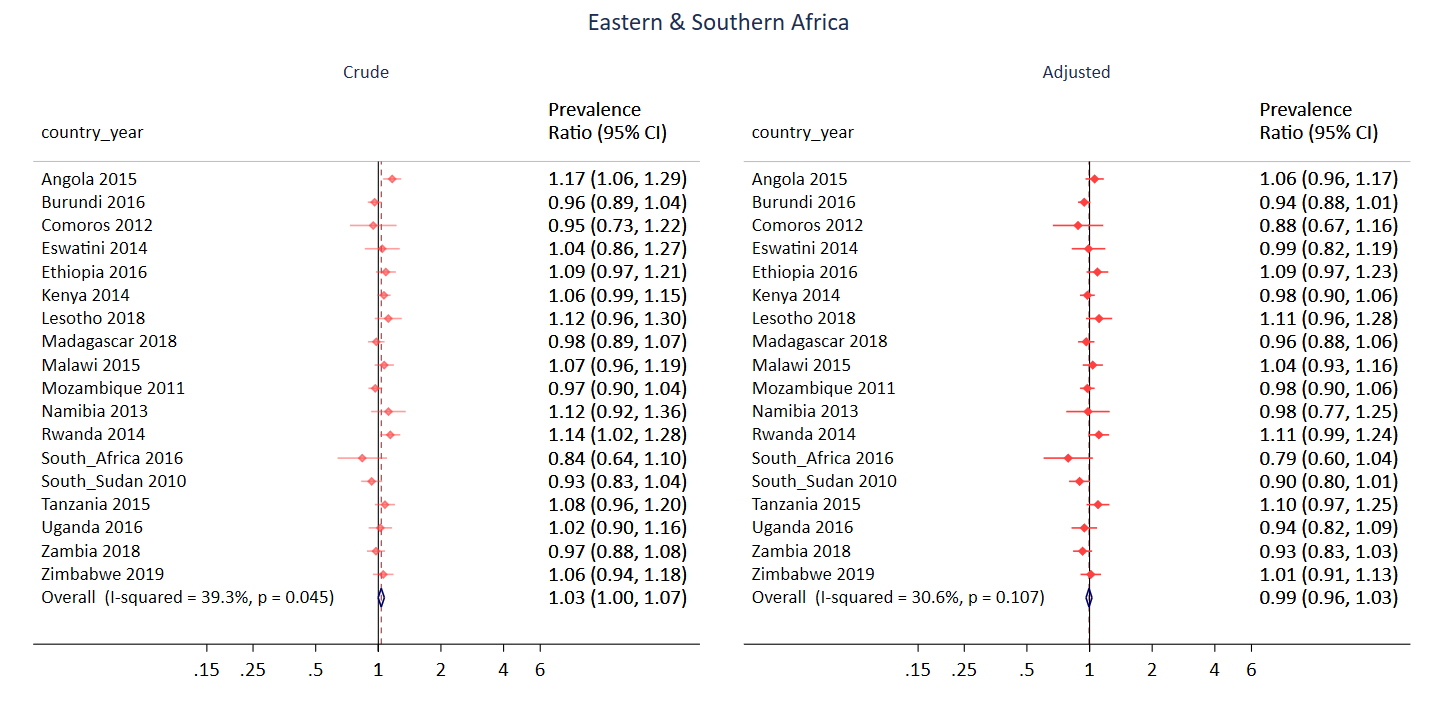


**Figure A38. Crude and adjusted prevalence ratio for stunting in FHH (no male) in Easter & Southern Africa**


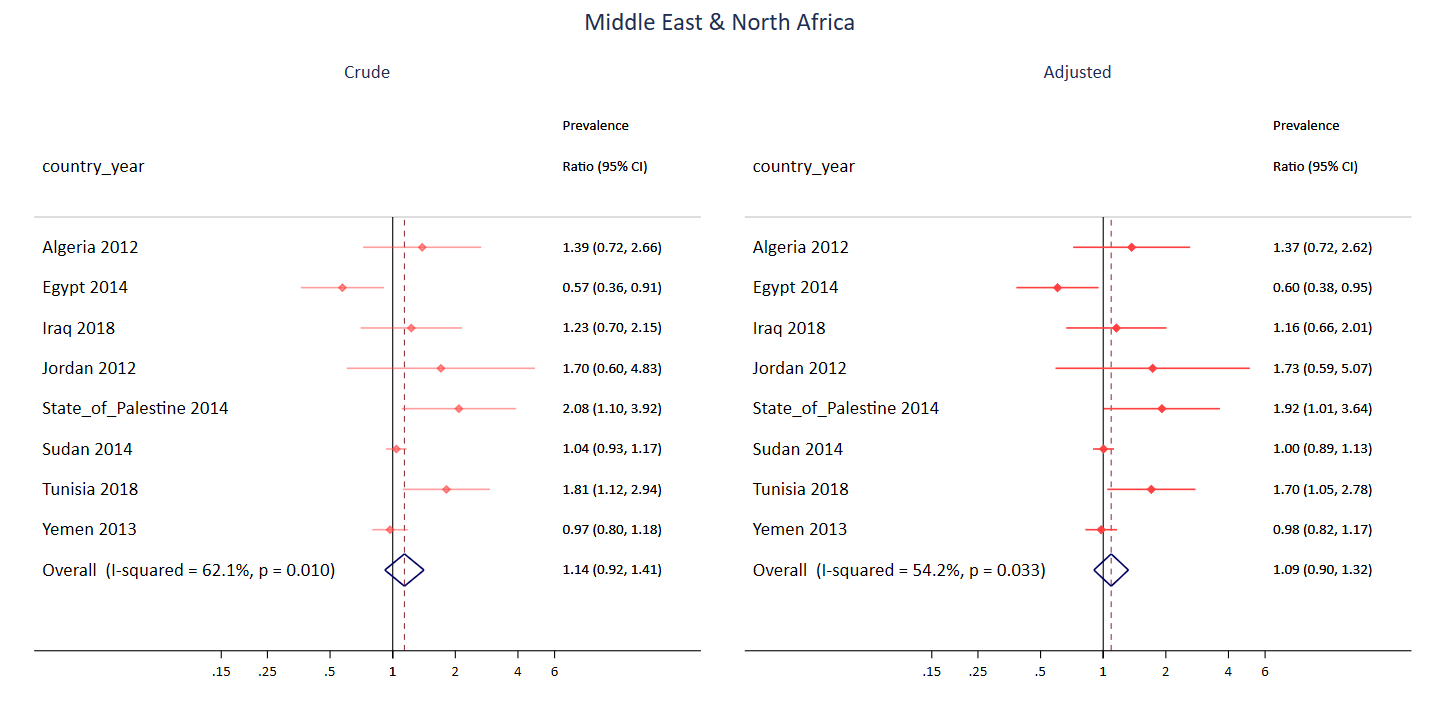


**Figure A39. Crude and adjusted prevalence ratio for stunting in FHH (no male) in Middle East & North Africa**


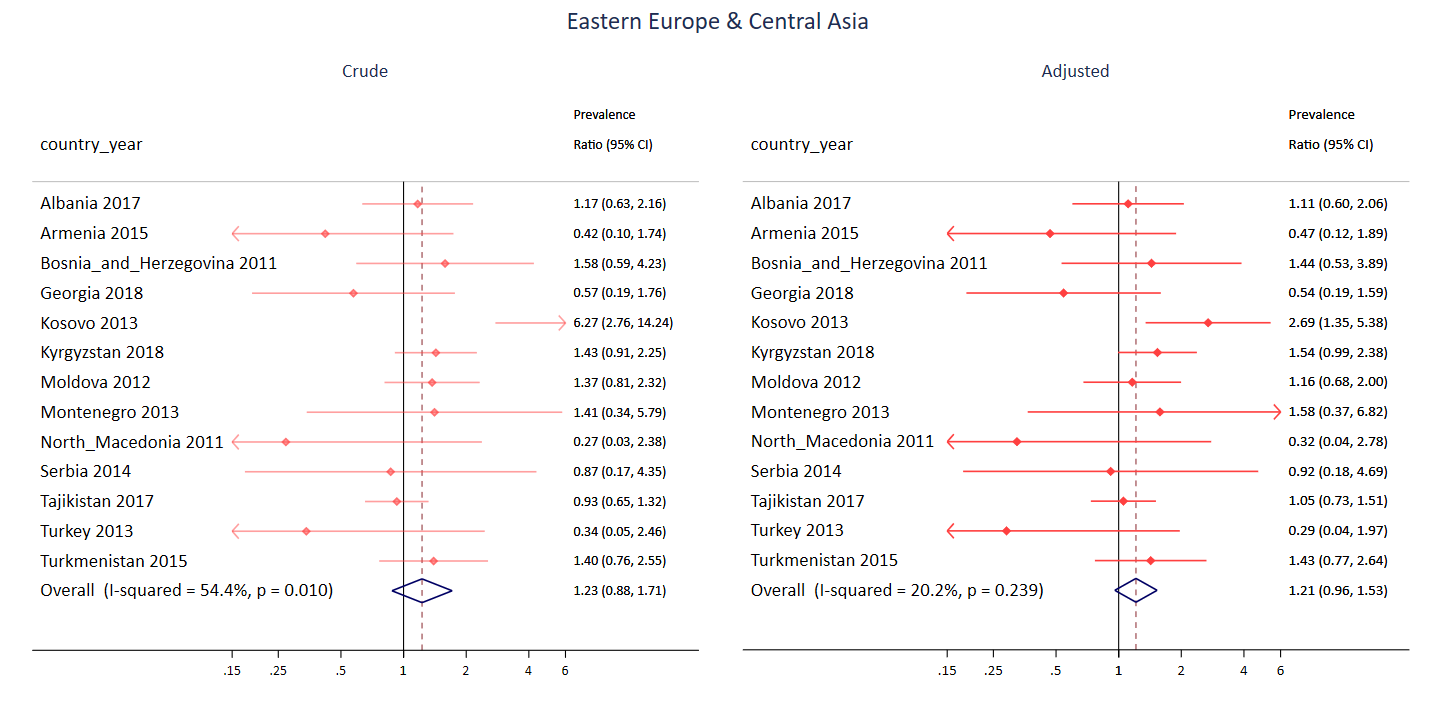


**Figure A40. Crude and adjusted prevalence ratio for stunting in FHH (no male) in Eastern Europe & Central Asia**

**
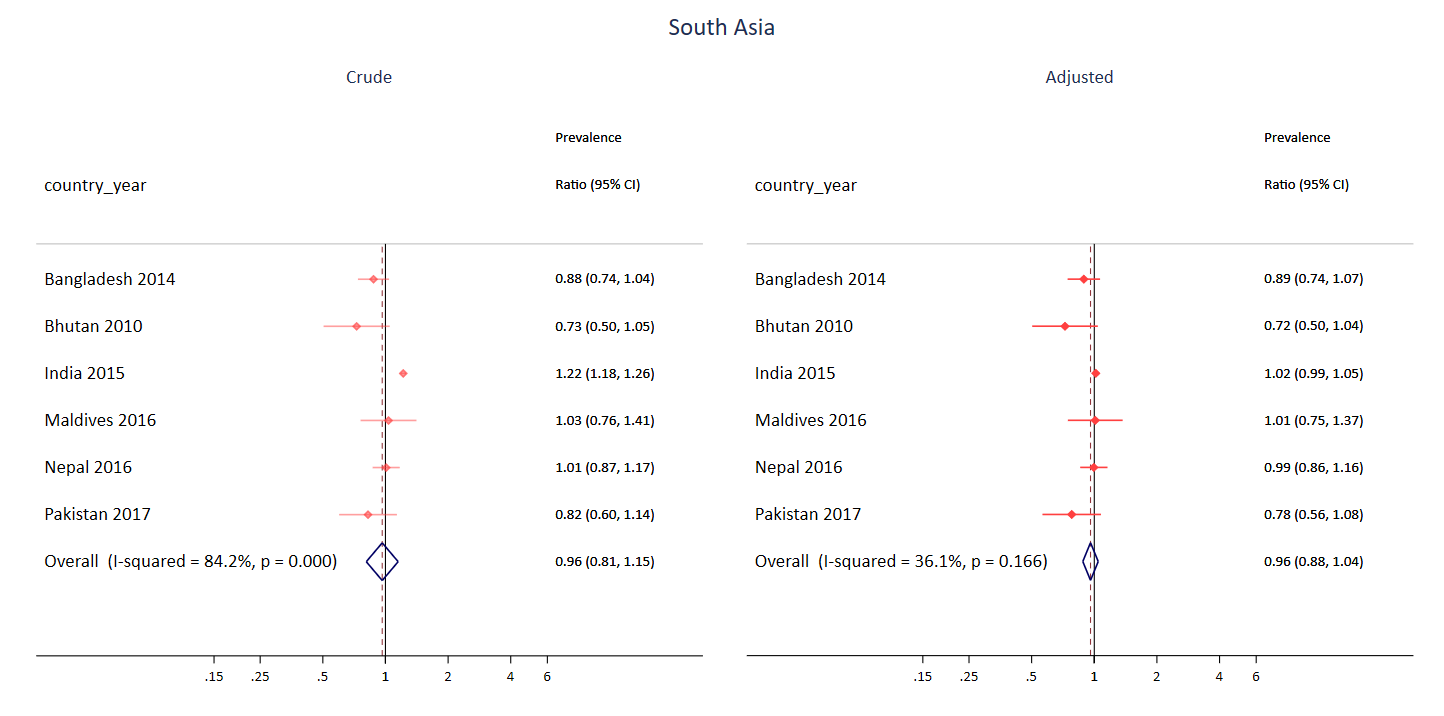
**

**Figure A41. Crude and adjusted prevalence ratio for stunting in FHH (no male) in South Asia**


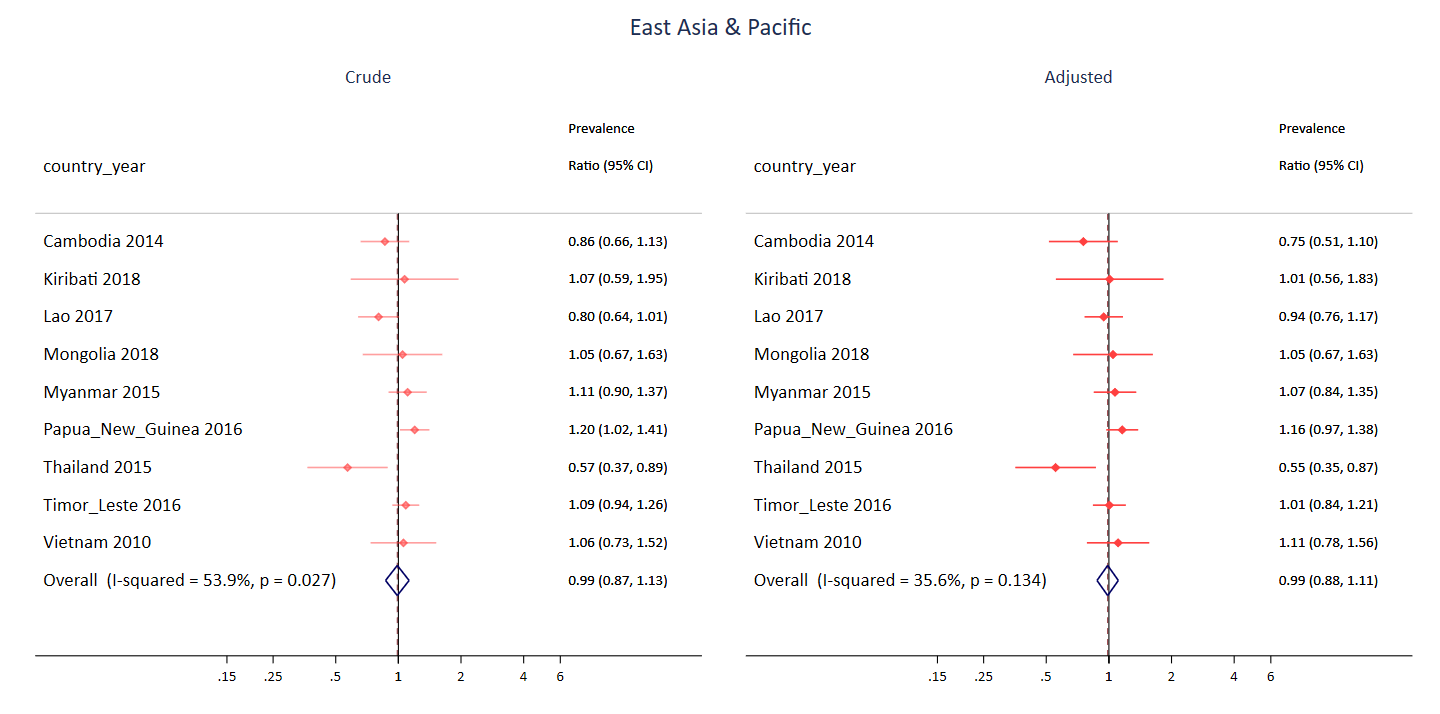


**Figure A42. Crude and adjusted prevalence ratio for stunting in FHH (no male) in East Asia & Pacific**

**
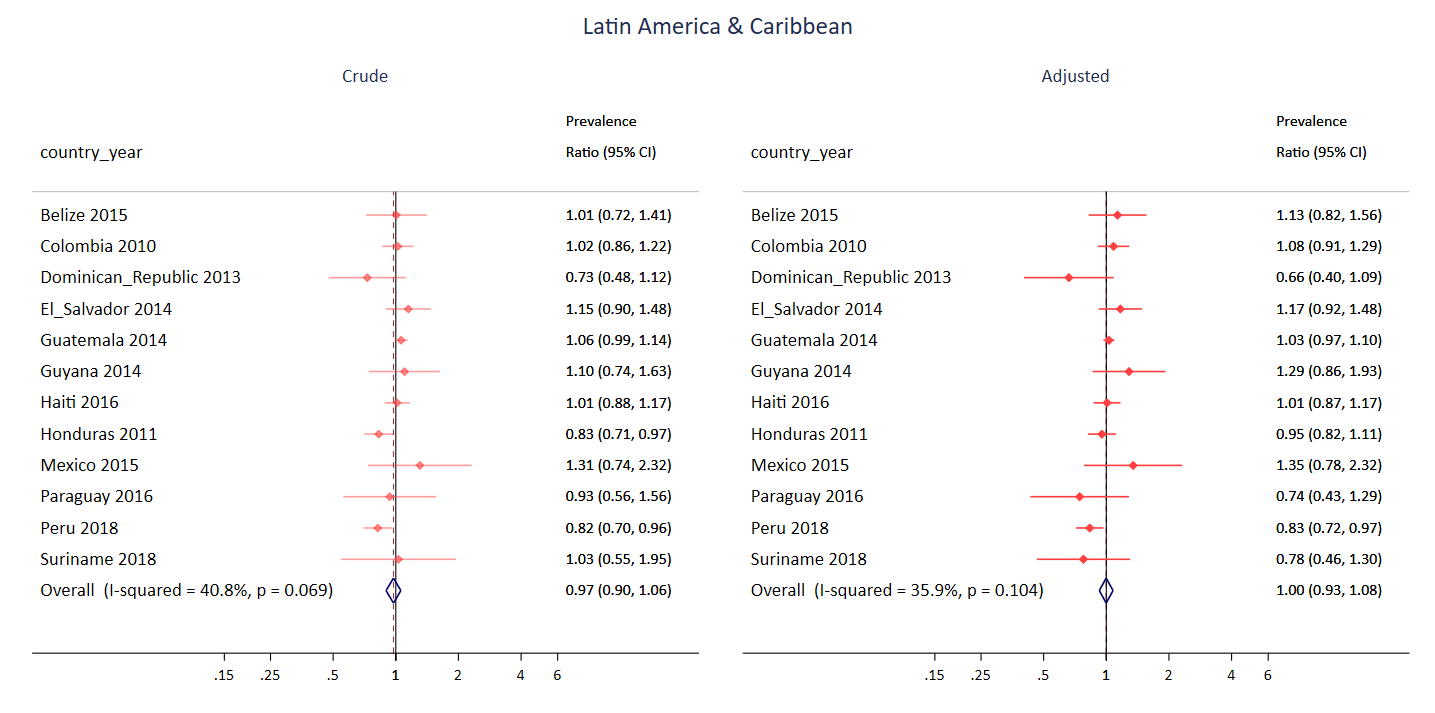
**

**Figure A43. Crude and adjusted prevalence ratio for stunting in FHH (no male) in Latin America & Caribbean**

**
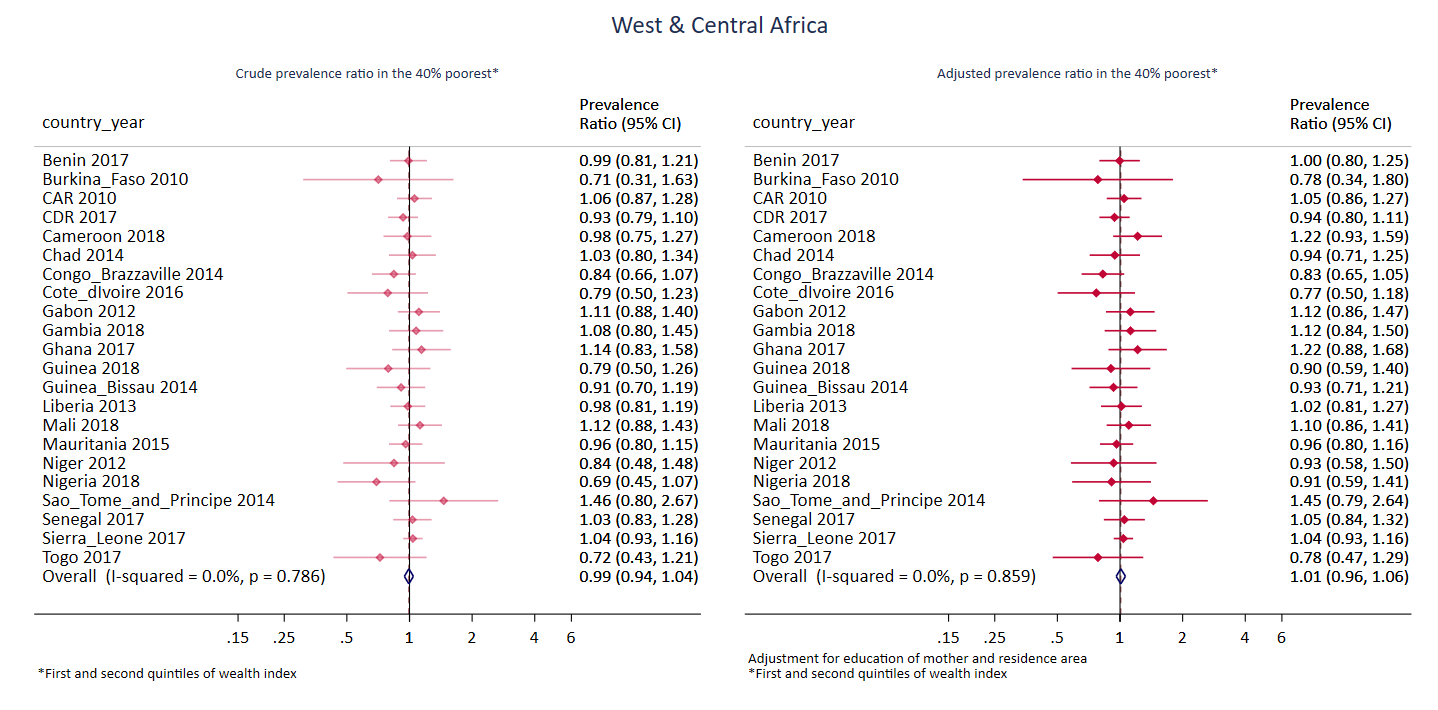
**

**Figure A44. Crude and adjusted prevalence ratio for stunting in FHH (any male) in West & Central Africa. Analysis restricted to 40% poorest in each country.**


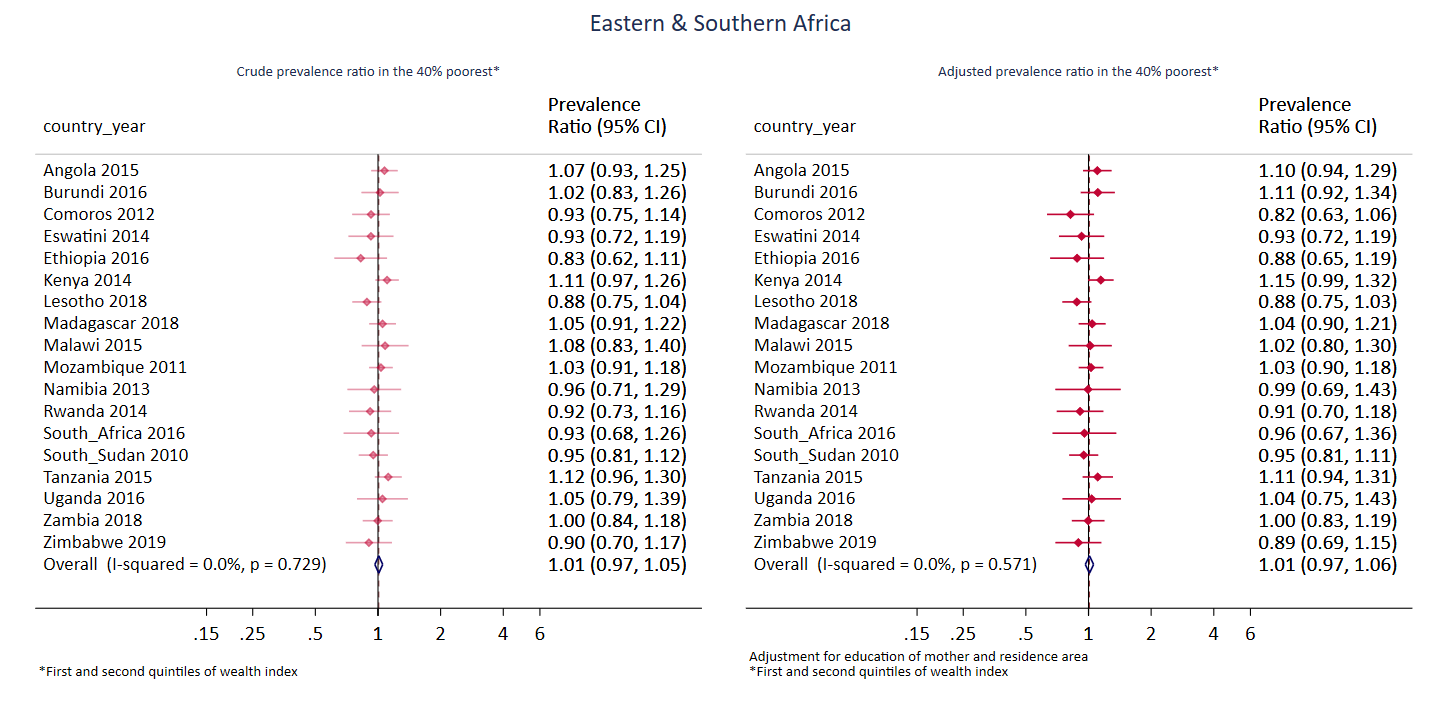


**Figure A45. Crude and adjusted prevalence ratio for stunting in FHH (any male) in Easter & Southern Africa. Analysis restricted to 40% poorest in each country.**

**
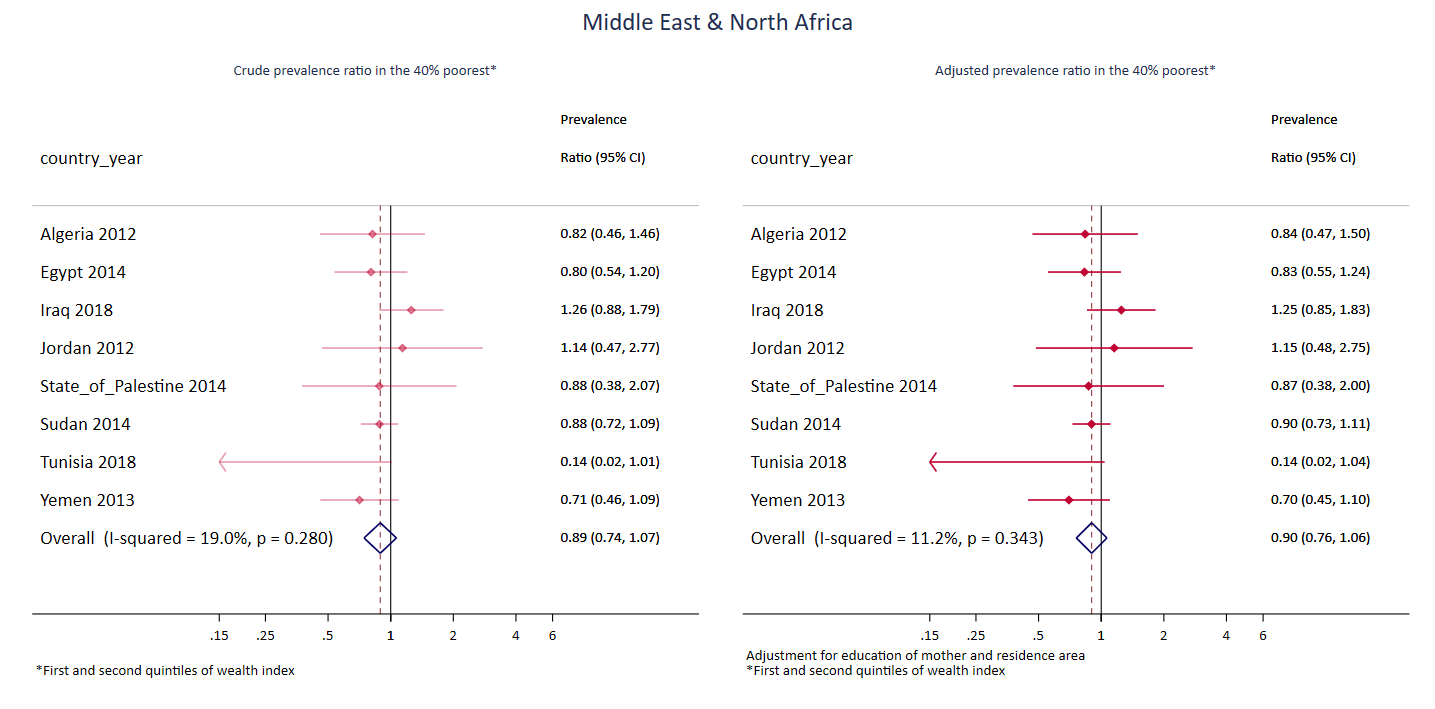
**

**Figure A46. Crude and adjusted prevalence ratio for stunting in FHH (any male) in Middle East & North Africa. Analysis restricted to 40% poorest in each country.**

**
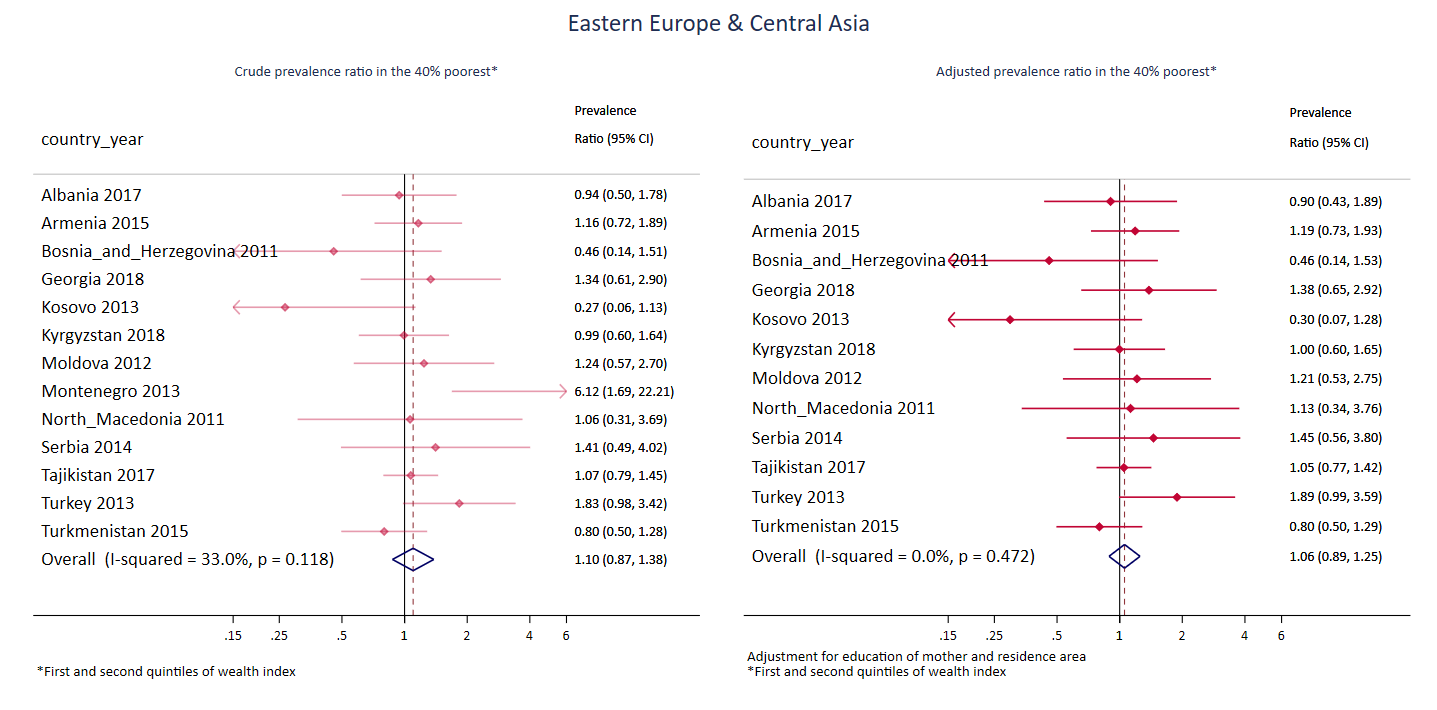
**

**Figure A47. Crude and adjusted prevalence ratio for stunting in FHH (any male) in Eastern Europe & Central Asia. Analysis restricted to 40% poorest in each country.**

**
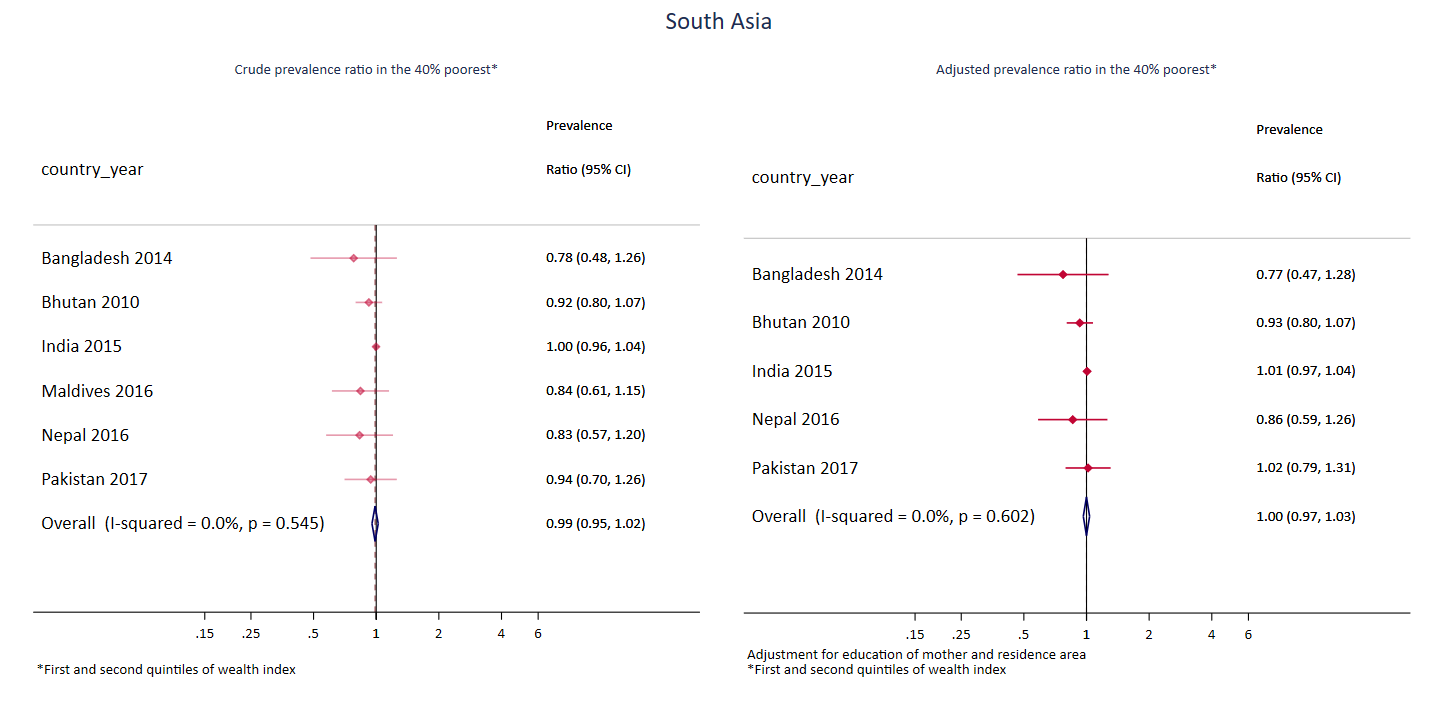
**

**Figure A48. Crude and adjusted prevalence ratio for stunting in FHH (any male) in South Asia. Analysis restricted to 40% poorest in each country.**

**
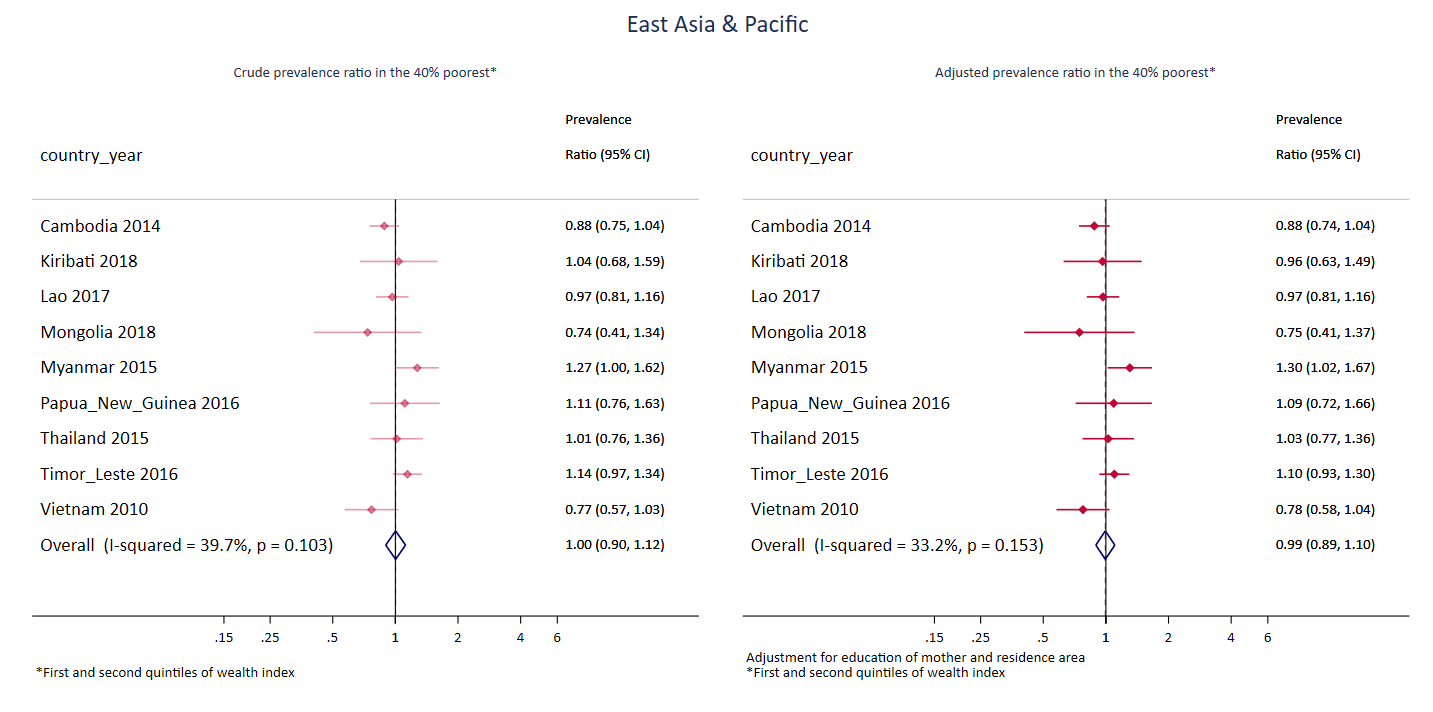
**

**Figure A49. Crude and adjusted prevalence ratio for stunting in FHH (any male) in East Asia & Pacific. Analysis restricted to 40% poorest in each country.**

**
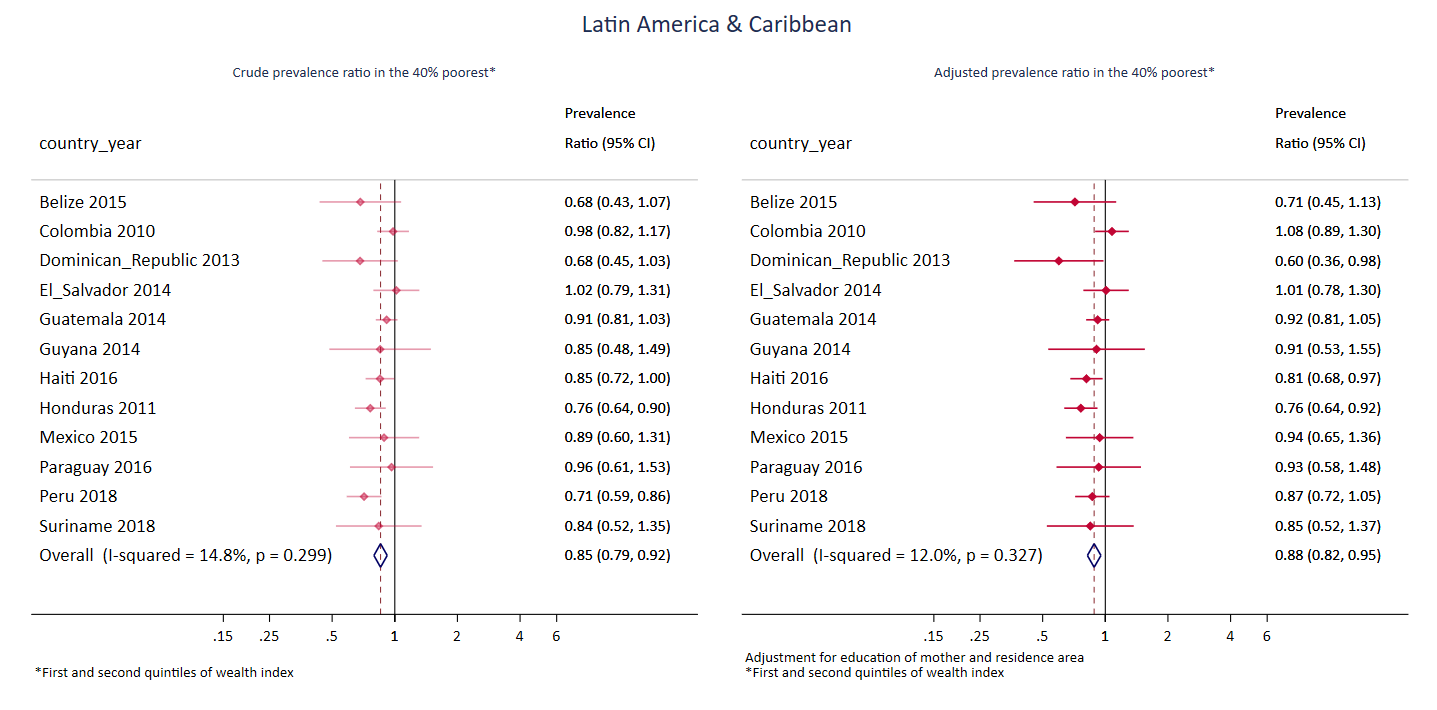
**

**Figure A50. Crude and adjusted prevalence ratio for stunting in FHH (any male) in Latin America & Caribbean. Analysis restricted to 40% poorest in each country.**

**
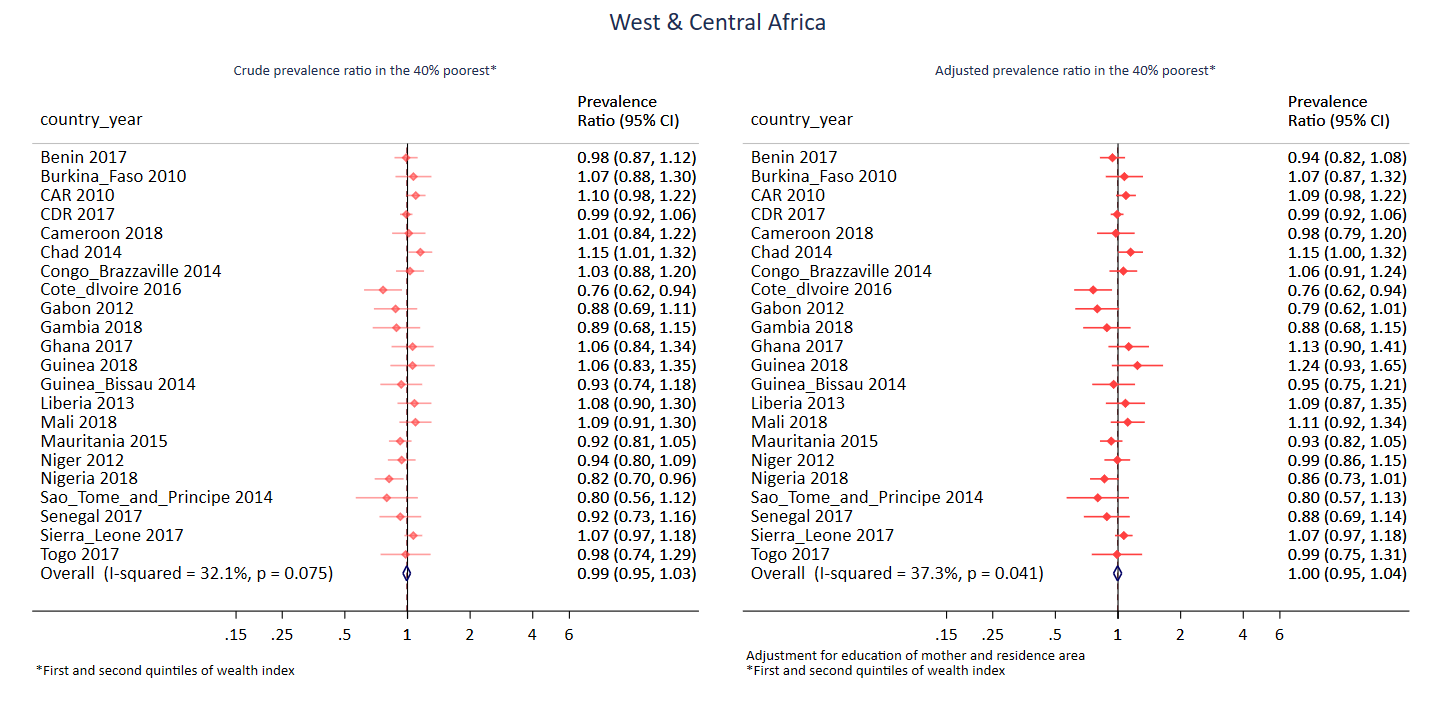
**

**Figure A51. Crude and adjusted prevalence ratio for stunting in FHH (no male) in West & Central Africa. Analysis restricted to 40% poorest in each country.**

**
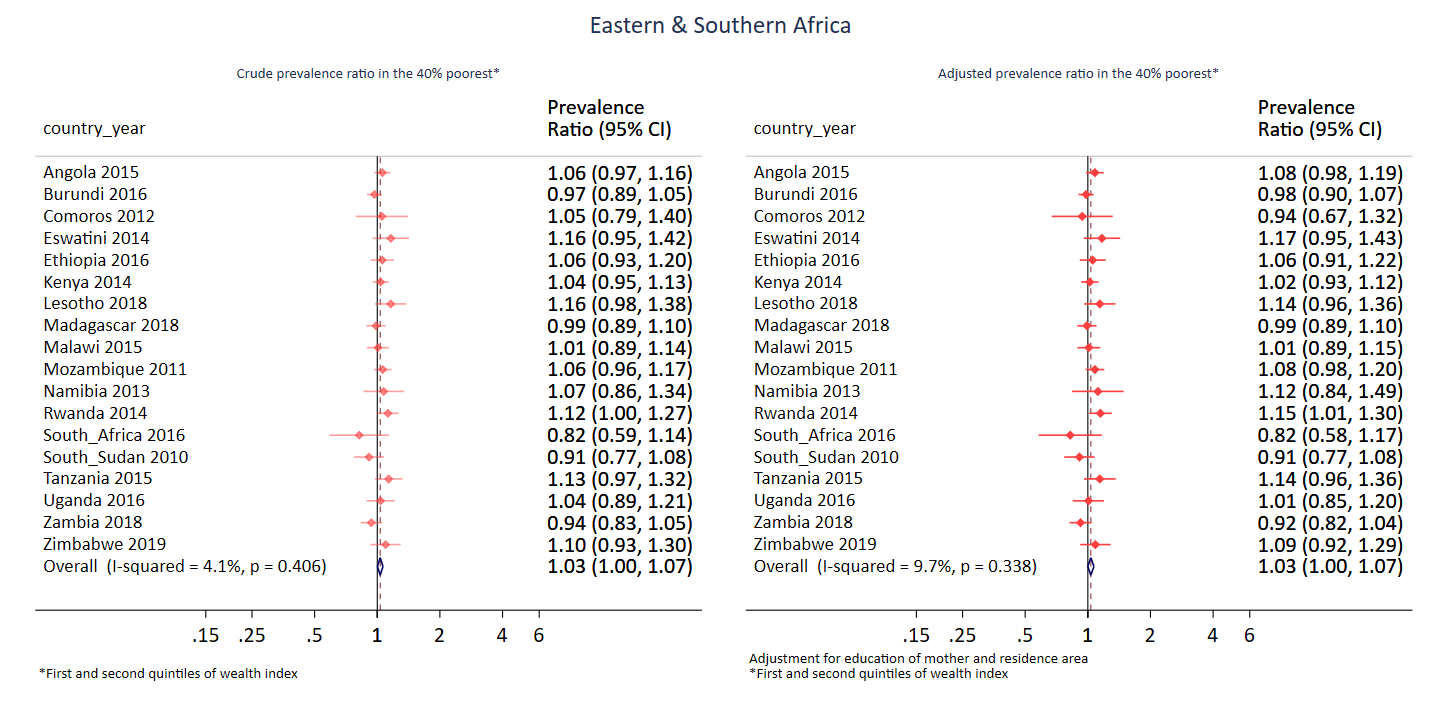
**

**Figure A52. Crude and adjusted prevalence ratio for stunting in FHH (no male) in Easter & Southern Africa. Analysis restricted to 40% poorest in each country.**

**
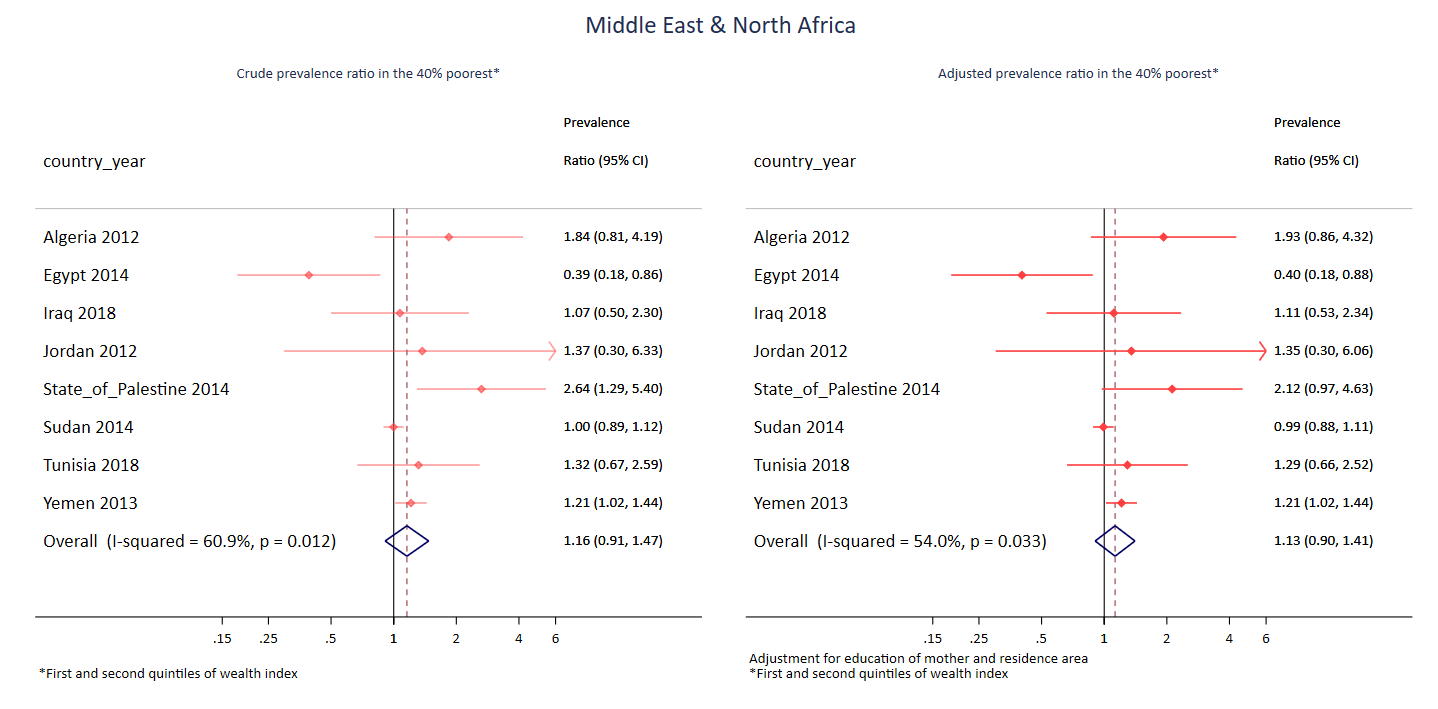
**

**Figure A53. Crude and adjusted prevalence ratio for stunting in FHH (no male) in Middle East & North Africa. Analysis restricted to 40% poorest in each country.**

**
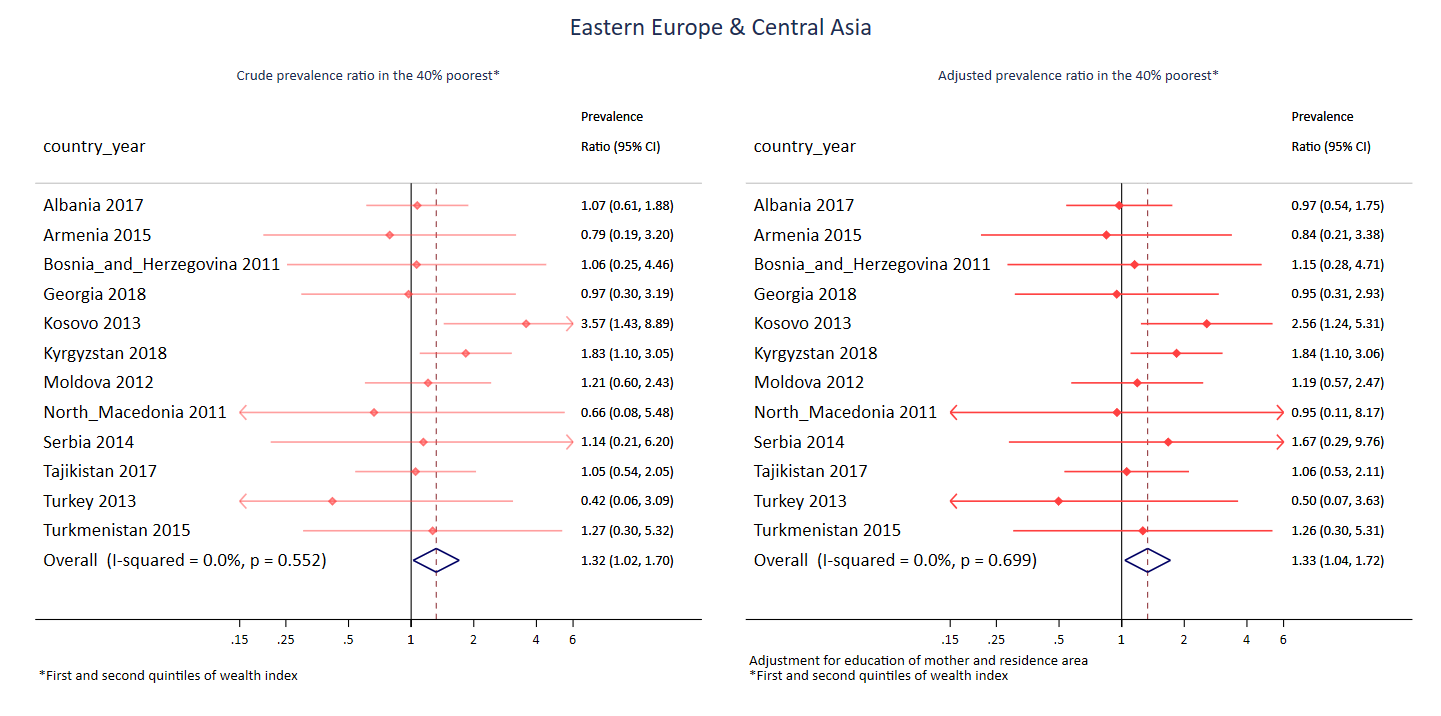
**

**Figure A54. Crude and adjusted prevalence ratio for stunting in FHH (no male) in Eastern Europe & Central Asia. Analysis restricted to 40% poorest in each country.**

**
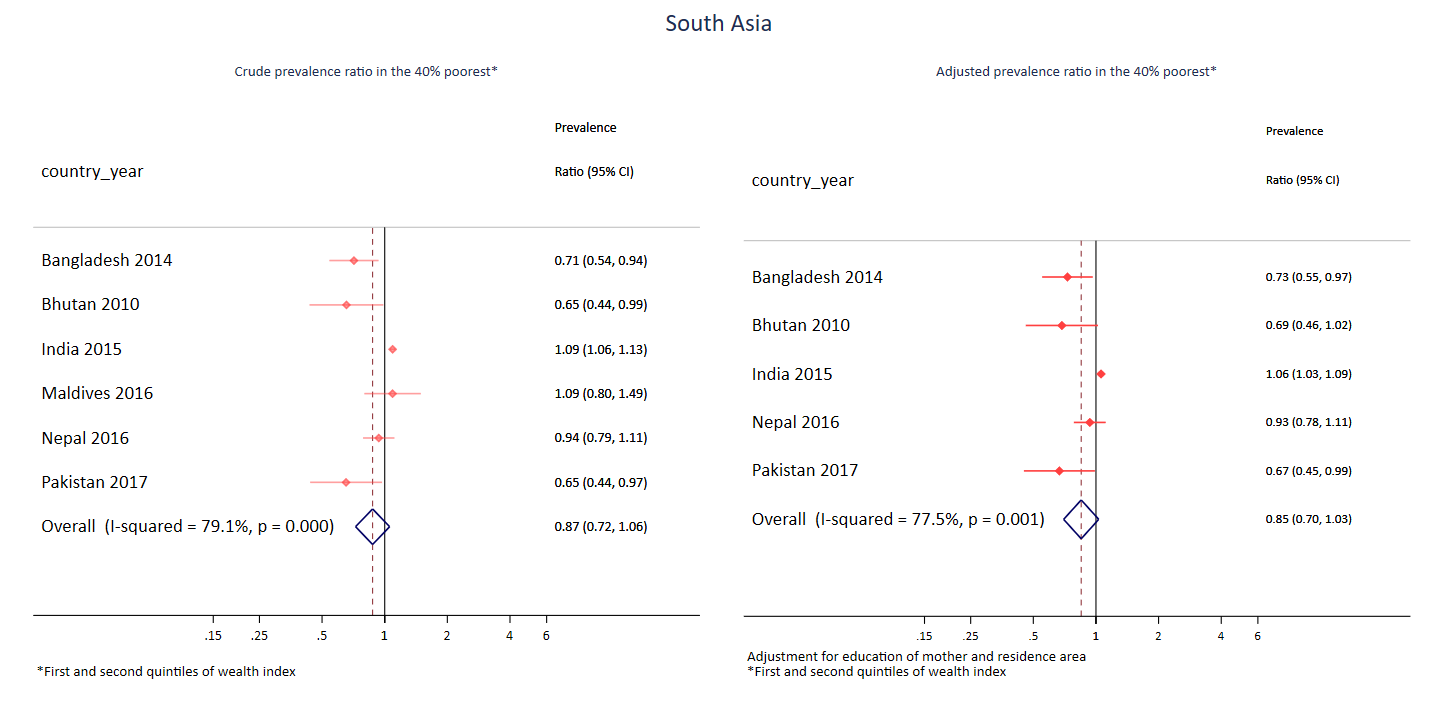
**

**Figure A55. Crude and adjusted prevalence ratio for stunting in FHH (no male) in South Asia. Analysis restricted to 40% poorest in each country.**


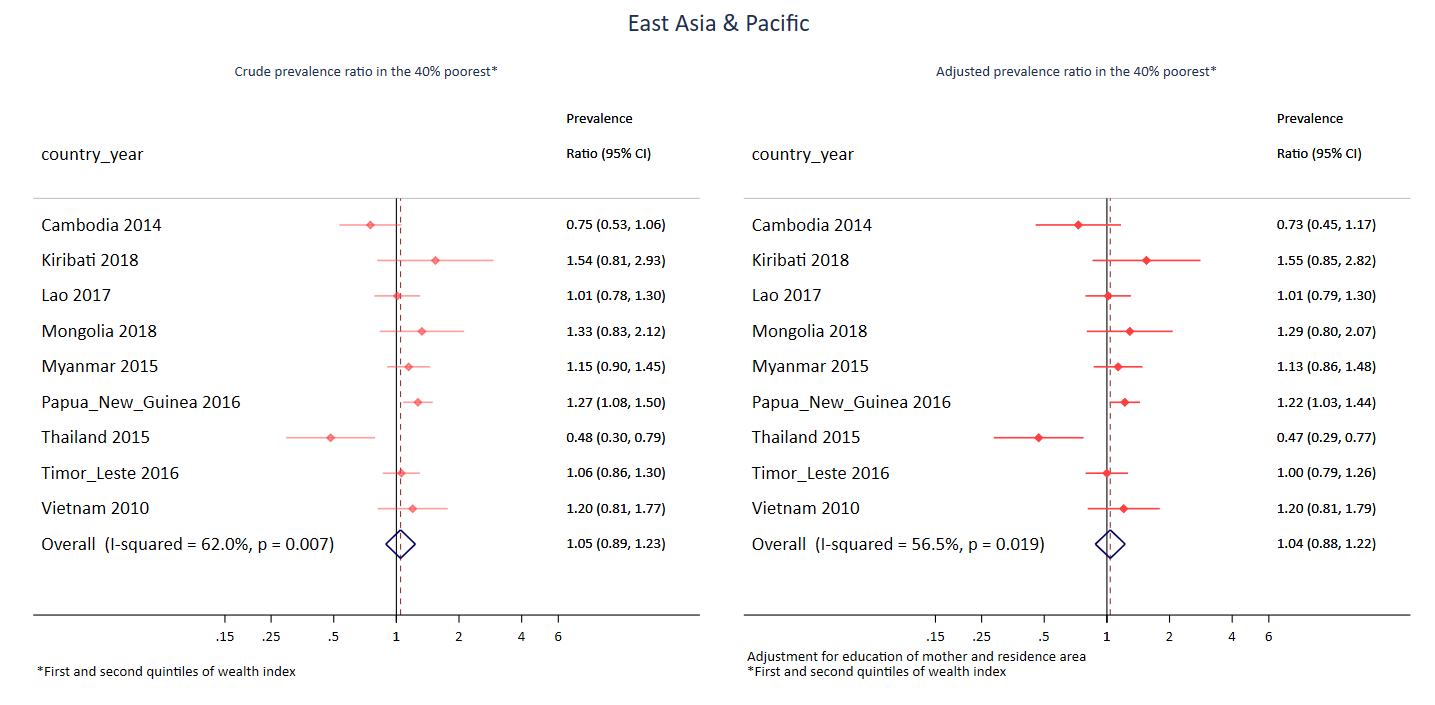


**Figure A56. Crude and adjusted prevalence ratio for stunting in FHH (no male) in East Asia & Pacific. Analysis restricted to 40% poorest in each country.**

**
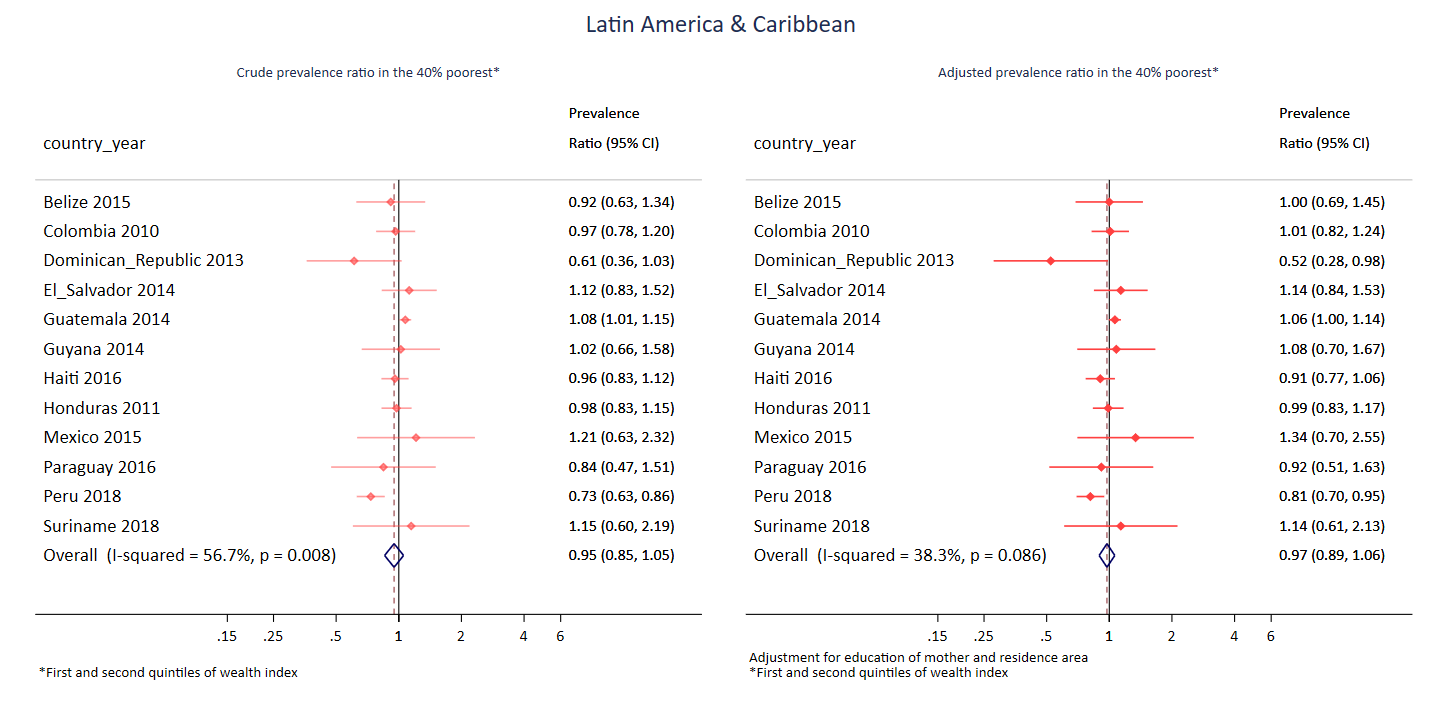
**

**Figure A57. Crude and adjusted prevalence ratio for stunting in FHH (no male) in Latin America & Caribbean. Analysis restricted to 40% poorest in each country.**

**Table A4. Prevalence ratios of full immunization adjusted for wealth index, maternal education, area of residence and mother living in polygynous union.**

|  |  | **FHH any male** | | **FHH no male** | |
| --- | --- | --- | --- | --- | --- |
| **Country** | **Year** | **PR** | **95% CI** | **RP** | **95% CI** |
| Afghanistan | 2015 | 1.11 | (0.69 - 1.79) | 1.42 | (0.91 - 2.21) |
| Algeria | 2012 | 1.03 | (0.93 - 1.13) | 1.26 | (1.18 - 1.34) |
| Angola | 2015 | 1.06 | (0.84 - 1.33) | 1.02 | (0.84 - 1.23) |
| Benin | 2017 | 1.15 | (0.98 - 1.33) | 0.89 | (0.78 - 1.03) |
| Burkina_Faso | 2010 | 1.03 | (0.89 - 1.19) | 0.92 | (0.81 - 1.04) |
| Burundi | 2016 | 0.92 | (0.82 - 1.04) | 1.00 | (0.95 - 1.05) |
| CAR | 2010 | 1.75 | (1.07 - 2.86) | 1.36 | (0.87 - 2.11) |
| Cambodia | 2014 | 0.96 | (0.87 - 1.06) | 1.18 | (0.97 - 1.43) |
| Cameroon | 2018 | 1.16 | (1.01 - 1.34) | 0.93 | (0.80 - 1.10) |
| Chad | 2014 | 1.00 | (0.71 - 1.42) | 0.66 | (0.46 - 0.94) |
| Comoros | 2012 | 0.90 | (0.77 - 1.06) | 0.68 | (0.45 - 1.01) |
| Congo_Brazzaville | 2014 | 0.82 | (0.46 - 1.47) | 0.91 | (0.61 - 1.37) |
| CDR | 2017 | 0.55 | (0.34 - 0.90) | 0.98 | (0.77 - 1.25) |
| Cote_dIvoire | 2016 | 0.97 | (0.71 - 1.33) | 1.28 | (1.03 - 1.61) |
| Egypt | 2014 | 1.02 | (0.94 - 1.11) | 0.95 | (0.80 - 1.12) |
| El_Salvador | 2014 | 1.01 | (0.93 - 1.10) | 0.89 | (0.77 - 1.03) |
| Eswatini | 2014 | 1.04 | (0.91 - 1.18) | 1.06 | (0.95 - 1.17) |
| Ethiopia | 2016 | 0.77 | (0.57 - 1.05) | 0.73 | (0.55 - 0.98) |
| Gabon | 2012 | 1.34 | (0.97 - 1.86) | 1.13 | (0.85 - 1.52) |
| Gambia | 2018 | 1.03 | (0.93 - 1.14) | 0.93 | (0.81 - 1.06) |
| Ghana | 2017 | 0.81 | (0.65 - 1.01) | 0.91 | (0.78 - 1.05) |
| Guinea | 2018 | 0.90 | (0.59 - 1.38) | 1.24 | (0.84 - 1.83) |
| Guinea_Bissau | 2014 | 1.00 | (0.87 - 1.16) | 0.87 | (0.70 - 1.09) |
| Guyana | 2014 | 0.92 | (0.74 - 1.15) | 0.90 | (0.72 - 1.14) |
| Haiti | 2016 | 0.96 | (0.80 - 1.15) | 0.99 | (0.81 - 1.21) |
| India | 2015 | 1.04 | (1.01 - 1.08) | 1.02 | (0.97 - 1.07) |
| Iraq | 2018 | 1.10 | (0.97 - 1.24) | 1.19 | (0.69 - 2.04) |
| Jordan | 2012 | 1.00 | (0.88 - 1.14) | 1.03 | (0.96 - 1.11) |
| Kenya | 2014 | 0.82 | (0.71 - 0.95) | 0.99 | (0.93 - 1.06) |
| Kiribati | 2018 | 1.36 | (0.80 - 2.30) | 0.70 | (0.17 - 2.92) |
| Lao | 2017 | 0.98 | (0.81 - 1.19) | 0.55 | (0.28 - 1.07) |
| Lesotho | 2018 | 0.97 | (0.83 - 1.14) | 0.71 | (0.54 - 0.95) |
| Liberia | 2013 | 0.88 | (0.74 - 1.06) | 1.01 | (0.84 - 1.21) |
| Madagascar | 2018 | 0.86 | (0.62 - 1.20) | 0.88 | (0.72 - 1.07) |
| Malawi | 2015 | 0.94 | (0.85 - 1.03) | 0.94 | (0.88 – 1.00) |
| Maldives | 2016 | 0.94 | (0.82 - 1.08) | 0.97 | (0.82 - 1.15) |
| Mali | 2018 | 1.34 | (1.09 - 1.65) | 0.87 | (0.69 - 1.10) |
| Mauritania | 2015 | 0.96 | (0.70 - 1.32) | 0.97 | (0.74 - 1.27) |
| Mozambique | 2011 | 0.95 | (0.84 - 1.06) | 1.04 | (0.96 - 1.14) |
| Myanmar | 2015 | 1.12 | (0.92 - 1.35) | 0.87 | (0.61 - 1.22) |
| Namibia | 2013 | 1.05 | (0.94 - 1.18) | 0.94 | (0.83 - 1.06) |
| Nepal | 2016 | 1.08 | (0.98 - 1.19) | 1.04 | (0.95 - 1.14) |
| Niger | 2012 | 0.74 | (0.43 - 1.28) | 1.11 | (0.97 - 1.28) |
| Nigeria | 2018 | 1.03 | (0.81 - 1.30) | 1.07 | (0.93 - 1.22) |
| Pakistan | 2017 | 1.18 | (1.05 - 1.33) | 1.21 | (1.03 - 1.43) |
| Panama | 2013 | 0.86 | (0.64 - 1.15) | 1.25 | (1.07 - 1.47) |
| Papua_New_Guinea | 2016 | 0.73 | (0.54 - 1.01) | 0.69 | (0.38 - 1.26) |
| Philippines | 2017 | 0.89 | (0.76 - 1.04) | 0.95 | (0.74 - 1.22) |
| Rwanda | 2014 | 0.96 | (0.90 - 1.04) | 0.94 | (0.88 – 1.00) |
| Sao_Tome_and_Principe | 2014 | 0.96 | (0.78 - 1.19) | 1.10 | (0.99 - 1.23) |
| Senegal | 2017 | 0.92 | (0.84 – 1.00) | 1.02 | (0.93 - 1.12) |
| Sierra_Leone | 2017 | 1.00 | (0.89 - 1.11) | 0.90 | (0.81 – 1.00) |
| South_Africa | 2016 | 0.99 | (0.80 - 1.22) | 1.02 | (0.86 - 1.22) |
| South_Sudan | 2010 | 0.61 | (0.26 - 1.43) | 0.85 | (0.41 - 1.75) |
| State_of_Palestine | 2014 | 0.92 | (0.77 - 1.09) | 1.00 | (0.70 - 1.44) |
| Sudan | 2014 | 1.10 | (0.98 - 1.23) | 0.93 | (0.79 - 1.09) |
| Tajikistan | 2017 | 1.02 | (0.94 - 1.12) | 0.96 | (0.68 - 1.35) |
| Tanzania | 2015 | 0.98 | (0.86 - 1.12) | 1.00 | (0.90 - 1.11) |
| Thailand | 2015 | 0.94 | (0.81 - 1.09) | 1.04 | (0.90 - 1.20) |
| Timor_Leste | 2016 | 0.99 | (0.80 - 1.23) | 0.94 | (0.66 - 1.34) |
| Togo | 2017 | 0.92 | (0.64 - 1.30) | 0.91 | (0.71 - 1.17) |
| Uganda | 2016 | 0.93 | (0.79 - 1.11) | 1.00 | (0.91 - 1.10) |
| Vietnam | 2010 | 1.05 | (0.97 - 1.15) | 1.03 | (0.83 - 1.28) |
| Yemen | 2013 | 0.92 | (0.65 - 1.29) | 0.94 | (0.63 - 1.41) |
| Zambia | 2018 | 0.97 | (0.85 - 1.10) | 0.96 | (0.87 - 1.06) |
| Zimbabwe | 2019 | 1.04 | (0.97 - 1.11) | 0.95 | (0.87 - 1.03) |

**Table A5. Prevalence ratios of stunting adjusted for wealth index, maternal education, area of residence and mother living in polygynous union.**

|  |  | **FHH any male** | | **FHH no male** | |
| --- | --- | --- | --- | --- | --- |
| **Country** | **Year** | **PR** | **95% CI** | **RP** | **95% CI** |
| Algeria | 2012 | 1.06 | (0.79 - 1.43) | 1.49 | (0.73 - 3.04) |
| Angola | 2015 | 0.94 | (0.80 - 1.12) | 1.06 | (0.96 - 1.17) |
| Barbados | 2012 | 2.07 | (0.78 - 5.47) | 1.62 | (0.57 - 4.62) |
| Benin | 2017 | 0.98 | (0.83 - 1.15) | 0.98 | (0.89 - 1.09) |
| Bhutan | 2010 | 0.97 | (0.87 - 1.08) | 0.72 | (0.49 - 1.06) |
| Burkina_Faso | 2010 | 0.91 | (0.62 - 1.34) | 1.02 | (0.88 - 1.19) |
| Burundi | 2016 | 1.11 | (0.96 - 1.28) | 0.94 | (0.88 - 1.01) |
| CAR | 2010 | 0.98 | (0.84 - 1.15) | 1.12 | (1.02 - 1.22) |
| Cambodia | 2014 | 0.98 | (0.85 - 1.14) | 0.75 | (0.51 - 1.10) |
| Cameroon | 2018 | 1.06 | (0.84 - 1.34) | 1.01 | (0.85 - 1.20) |
| Chad | 2014 | 0.95 | (0.81 - 1.13) | 1.16 | (1.05 - 1.29) |
| Comoros | 2012 | 0.85 | (0.71 - 1.02) | 0.89 | (0.68 - 1.18) |
| Congo_Brazzaville | 2014 | 0.97 | (0.73 - 1.29) | 0.93 | (0.79 - 1.09) |
| CDR | 2017 | 0.98 | (0.85 - 1.15) | 1.00 | (0.92 - 1.08) |
| Cote_dIvoire | 2016 | 0.96 | (0.72 - 1.29) | 0.82 | (0.65 - 1.02) |
| Egypt | 2014 | 0.96 | (0.70 - 1.32) | 0.59 | (0.38 - 0.93) |
| El_Salvador | 2014 | 1.10 | (0.90 - 1.34) | 1.17 | (0.92 - 1.49) |
| Eswatini | 2014 | 0.86 | (0.68 - 1.09) | 1.00 | (0.81 - 1.24) |
| Ethiopia | 2016 | 0.96 | (0.80 - 1.16) | 1.11 | (0.98 - 1.25) |
| Gabon | 2012 | 1.19 | (0.86 - 1.63) | 0.96 | (0.72 - 1.30) |
| Gambia | 2018 | 0.92 | (0.73 - 1.15) | 0.74 | (0.56 - 0.99) |
| Ghana | 2017 | 1.04 | (0.80 - 1.34) | 1.02 | (0.86 - 1.23) |
| Guinea | 2018 | 1.08 | (0.82 - 1.41) | 1.13 | (0.93 - 1.37) |
| Guinea_Bissau | 2014 | 1.02 | (0.84 - 1.24) | 0.97 | (0.76 - 1.25) |
| Guyana | 2014 | 1.11 | (0.76 - 1.63) | 1.32 | (0.84 - 2.08) |
| Haiti | 2016 | 0.86 | (0.74 – 1.00) | 1.00 | (0.85 - 1.17) |
| India | 2015 | 1.00 | (0.97 - 1.03) | 1.02 | (0.99 - 1.05) |
| Iraq | 2018 | 1.22 | (0.89 - 1.67) | 1.26 | (0.73 - 2.15) |
| Jordan | 2012 | 1.09 | (0.48 - 2.49) | 1.73 | (0.59 - 5.07) |
| Kenya | 2014 | 1.08 | (0.95 - 1.21) | 0.98 | (0.90 - 1.06) |
| Kiribati | 2018 | 1.00 | (0.70 - 1.42) | 0.88 | (0.47 - 1.64) |
| Kosovo | 2013 | 0.41 | (0.13 - 1.26) | 2.66 | (1.34 - 5.27) |
| Lao | 2017 | 0.93 | (0.82 - 1.07) | 0.95 | (0.76 - 1.20) |
| Lesotho | 2018 | 1.00 | (0.85 - 1.19) | 1.13 | (0.95 - 1.34) |
| Liberia | 2013 | 0.92 | (0.74 - 1.15) | 1.02 | (0.84 - 1.25) |
| Madagascar | 2018 | 1.03 | (0.89 - 1.20) | 0.96 | (0.88 - 1.06) |
| Malawi | 2015 | 1.01 | (0.82 - 1.24) | 1.02 | (0.91 - 1.14) |
| Maldives | 2016 | 0.79 | (0.58 - 1.06) | 0.99 | (0.72 - 1.36) |
| Mali | 2018 | 1.03 | (0.85 - 1.23) | 1.09 | (0.95 - 1.25) |
| Mauritania | 2015 | 1.03 | (0.91 - 1.17) | 1.07 | (0.96 - 1.20) |
| Mozambique | 2011 | 1.04 | (0.95 - 1.13) | 0.98 | (0.90 - 1.06) |
| Myanmar | 2015 | 1.18 | (0.96 - 1.45) | 1.07 | (0.84 - 1.36) |
| Namibia | 2013 | 1.10 | (0.84 - 1.44) | 0.98 | (0.77 - 1.25) |
| Nepal | 2016 | 0.95 | (0.72 - 1.24) | 0.99 | (0.85 - 1.16) |
| Niger | 2012 | 1.16 | (0.87 - 1.55) | 1.03 | (0.92 - 1.14) |
| Nigeria | 2018 | 1.17 | (0.92 - 1.47) | 0.87 | (0.77 - 0.98) |
| Pakistan | 2017 | 0.99 | (0.74 - 1.31) | 0.78 | (0.56 - 1.08) |
| Papua_New_Guinea | 2016 | 0.93 | (0.66 - 1.30) | 1.15 | (0.96 - 1.38) |
| Rwanda | 2014 | 0.91 | (0.73 - 1.12) | 1.10 | (0.99 - 1.24) |
| Sao_Tome_and_Principe | 2014 | 1.18 | (0.70 – 2.00) | 0.88 | (0.68 - 1.13) |
| Senegal | 2017 | 1.07 | (0.90 - 1.27) | 0.86 | (0.70 - 1.06) |
| Sierra_Leone | 2017 | 1.04 | (0.94 - 1.15) | 1.02 | (0.93 - 1.12) |
| South_Africa | 2016 | 0.83 | (0.58 - 1.18) | 0.79 | (0.60 - 1.03) |
| South_Sudan | 2010 | 0.89 | (0.78 – 1.00) | 0.90 | (0.80 - 1.02) |
| State_of_Palestine | 2014 | 0.82 | (0.39 - 1.74) | 2.10 | (0.98 - 4.47) |
| Sudan | 2014 | 0.85 | (0.70 - 1.04) | 1.02 | (0.90 - 1.15) |
| Suriname | 2018 | 0.92 | (0.62 - 1.37) | 0.89 | (0.52 - 1.53) |
| Tajikistan | 2017 | 0.97 | (0.08 - 1.18) | 1.07 | (0.75 - 1.54) |
| Tanzania | 2015 | 1.14 | (1.00 - 1.30) | 1.10 | (0.97 - 1.25) |
| Thailand | 2015 | 0.92 | (0.71 - 1.20) | 0.55 | (0.32 - 0.92) |
| Timor_Leste | 2016 | 1.02 | (0.89 - 1.18) | 1.00 | (0.84 - 1.21) |
| Togo | 2017 | 0.95 | (0.68 - 1.32) | 1.11 | (0.89 - 1.39) |
| Uganda | 2016 | 1.15 | (0.86 - 1.53) | 0.94 | (0.82 - 1.09) |
| Vietnam | 2010 | 0.96 | (0.78 - 1.19) | 1.22 | (0.86 - 1.73) |
| Yemen | 2013 | 0.95 | (0.78 - 1.16) | 0.98 | (0.82 - 1.17) |
| Zambia | 2018 | 1.17 | (1.02 - 1.33) | 0.94 | (0.85 - 1.04) |
| Zimbabwe | 2019 | 0.90 | (0.75 - 1.08) | 0.98 | (0.87 - 1.11) |
